# Relationship between dietary intake and growth and development in Chinese pupils

**DOI:** 10.3389/fpubh.2025.1454129

**Published:** 2025-02-12

**Authors:** Wen Fang, Ye Fu, Qin Li, Menghan Cheng, Miao Zhang, Yu Lu

**Affiliations:** ^1^Center for Environment and Health in Water Source Area of South-to-North Water Diversion, School of Public Health, Hubei University of Medicine, Shiyan, China; ^2^State Key Laboratory of Environmental Health (Incubating), Department of Occupational and Environmental Health, School of Public Health, Tongji Medical College, Huazhong University of Science and Technology, Wuhan, China

**Keywords:** dietary intake, growth and development, Chinese pupils, dietary diversity score, food intake frequency

## Abstract

**Background:**

A nutritional diet is essential for children’s growth and development. This study aimed to investigate the relationship between dietary intake and growth and development of pupils to provide more specific nutritional recommendations for their healthy growth.

**Methods:**

This cross-sectional study included 592 pupils, and standardized questionnaires were used to collect information on students’ sociodemographic characteristics, lifestyle habits and dietary intake. Growth and development assessment indicators were measured according to standard protocols. The multivariate generalized linear regression models adjusted for covariates were used to investigate the association between dietary intake and growth and development of pupils.

**Results:**

The generalized linear regression model showed that standing long jump of all pupils (*β* = −6.735, 95% *CI*: −12.064, −1.406) and body fat rate (*β* = −2.650, 95% *CI*: −4.794, −0.507), fat weight (*β* = −1.283, 95% *CI*: −2.286, −0.280) and chest circumference (*β* = −1.456, 95% *CI*: −2.789, −0.123) of boys were negatively correlated with dietary diversity score. Among all pupils, meat intake was positively correlated with chest circumference (*β* = 0.420, 95% *CI*: 0.119, 0.721) and negatively correlated with standing long jump(*β* = −1.991, 95% *CI*: −3.902, −0.080); milk intake was positively correlated with waist circumference (*β* = 0.470, 95% *CI*: 0.007, 0.932); soybean intake was negatively correlated with body fat rate (*β* = −0.583, 95% *CI*: −1.125, −0.042), fat weight (*β* = −0.262, 95% *CI*: −0.517, −0.006), and waist circumference (*β* = −0.607, 95% *CI*: −1.050, −0.164); and vegetable intake was negatively correlated with height (*β* = −0.290, 95% *CI*: −0.496, −0.084), and positively correlated with stature-sitting height index and waist circumference (*p* < 0.05). Certain associations retained significance even after stratified analysis based on gender and frequency of dietary intake.

**Conclusion:**

Dietary diversity score and their respective food groups such as meat, milk, coarse grain, soybean and vegetable will impact growth and development indicators, requiring purposefully controlled dietary intake.

## 1 Introduction

Childhood is a critical period for rapid physical growth and sustained development. The growth and development during this period not only affects adult height and weight (1), but also leads to developmental problems such as psychological disorders (2), cognitive dysfunction, impaired exercise capacity (3), and increases the risk of developing diabetes and cardiovascular disease (4, 5). Therefore, it is necessary to pay more attention to the growth and development of children.

Nutrition is one of the most important factors influencing children’s healthy growth and development. Different foods provide different nutrients that play a major role in body composition and physical fitness. A study on urban school-age children in India found that lower child height was associated with lower dietary energy and protein intake, reduced nutrient intake and high fat intake can lead to increased body fat percentage and malnutrition (6).

Another study about Chinese children and adolescents showed that higher protein intake was associated with shorter linear growth and a higher prevalence of stunting (7). However, there is currently no consensus on the relationship between dietary nutrition and growth and development. Moreover, according to the recommendations of the “Dietary Guidelines for Chinese School-aged Children (2022),” children need to consume soybeans, milk and dairy products, grains, vegetables, fruits, eggs, meat, and fish every day. Interestingly, the study found that the children’s diets have higher intakes of fat, protein, and cholesterol, but lower intakes of micronutrients, indicating that the actual intake of many students has not reached these recommendations (8).

Previous studies have explored the relationship between dietary nutrition intake and children’s growth and development by introducing dietary diversity scores (DDS). Still, the conclusions are inconsistent (9–11), and more focus has been on preschool children (12, 13), with fewer studies targeting pupils. Based on this, we conducted a cross-sectional study to investigate the relationship between dietary intake and growth and development of pupils to provide more specific nutritional recommendations for the healthy growth of pupils.

## 2 Materials and methods

### 2.1 Participants

In this cross-sectional study, all participants were pupils from Shiyan City, Hubei Province, who were surveyed in July 2022. Inclusion criteria were as follows: able to cooperate with physical examination and questionnaire filling and have signed informed consent; aged 7–13 years old. Exclusion criteria were as follows: suffering from major diseases or diseases known to affect growth; recently taking medications that may affect their dietary intake. A total of 653 pupils were recruited, and 592 pupils were finally included in the analysis after excluding those with incomplete questionnaires and missing physical examination indicators.

### 2.2 Questionnaire

Using a standardized questionnaire, data on the social-demographic characteristics, caregivers, lifestyle habits, residential information and dietary intake was collected. All questionnaires were filled out under the guidance of investigators, who had received training before the investigation. Age, gender, grade, main caregivers, caregivers’ current smoking status, dietary intake (fruit, vegetable, coarse grain, sugary beverage, salty and peppery, etc.) and lifestyle habits were all included in the data collection. Caregivers’ current smoking status was recorded as yes, no, quit smoking for more than 1 year, or quit smoking for less than 1 year. Lifestyle habits included weekday sleep time, weekend sleep time, midday nap time, daily homework time, and daily physical activity (PA) time in the past 3 months, etc. PA time per day was recorded as less than 30 min, 30 min to 1 h, and greater than or equal to 1 h. The average weekly nocturnal sleep time was calculated by multiplying the weekday sleep time by 5, the weekend sleep time multiplied by 2, and then dividing the sum by 7. Additionally, written informed consent was obtained from all subjects.

### 2.3 Assessment of growth and development

Trained surveyors measured growth and development indicators according to the methods described in the Chinese Students’ Physical Fitness and Health Research Report 2010 (14) and the 2014 National Student Physical Fitness and Health Survey Manual (15). The height and sitting height are measured by the instrument of height and sitting height meter (Jiangsu Suhong Medical Equipment Limited Company, China). The subjects are required to took off their shoes and hats, keep their heads upright, and keep their eyes level. The weight, body fat rate, and fat weight were measured by an smart electronic scale (Xiaomi Company, China). During the measurements, subjects were asked to wear loose clothing and remove their shoes and socks. Body mass index (BMI) was calculated as weight divided by height squared (kg/m^2^). The stature-sitting height index was calculated as the ratio between sitting height and body height. These measurements were recorded in two decimal places. When measuring waist circumference and chest circumference, subjects were asked to stand naturally, with their feet shoulder-width apart, shoulders relaxed, and to breathe gently without tightening their abdomen or holding their breath. The waist circumference was obtained by wrapping a measuring tape around the upper edge of the belly button at a level of 1 cm with a bare waist and abdomen. The chest circumference was measured using a tape measure with the arms hanging naturally. Both measured values were recorded in one decimal place. The standing long jump is an indicator that reflects lower limb explosive power and physical coordination ability (16), which can indirectly evaluate overall physical fitness and growth and development status. It needs to be tested on a non slip hard surface. The subjects were asked to jump the furthest possible distance from the take-off line while swinging both arms. The surveyor measured the horizontal distance between the closest landing site’s trailing edge and the take-off line’s trailing edge using a tape measure. Each subject was given three chances, and the best score was recorded in centimeters, no decimal places included. Handgrip strength was measured using a handgrip dynamometer while the subject was standing, with their feet placed half a step apart and their arm hanging naturally. Each subject was required to measure three times with both hands, and the largest reading was recorded as grip strength in kilograms, rounded to one decimal place.

### 2.4 Assessment of dietary intake

The dietary intake data was collected through a specially designed food frequency questionnaire, which was completed independently by the pupils after investigator instruction. According to the recommendations of the “Dietary Guidelines for Chinese School-aged Children (2022),” dietary intake included the weekly frequency of breakfast and weekly intake frequency of vegetables, fruits, meat, coarse grains, milk, eggs, fish, and soybeans in the past 3 months. Food intake frequency is typically classified as less than once a week, 1 to 2 times a week, 3 to 5 times a week, and more than 5 times a week.

The dietary diversity score is one of the indicators used to assess nutritional adequacy and overall diet quality, reflecting the consumption of various foods between and within each food group (9, 17). The higher dietary diversity score indicates better micronutrient adequacy and overall dietary quality (12). According to our design, eight food groups were utilized to evaluate dietary diversity, including vegetables, fruits, meat and meat products (including pork, beef and mutton, offal, etc.), coarse grains (corn, millet, sweet potato, yam, etc.), eggs, fish or fish products, milk or milk products, and soybeans or soybean products (soymilk, tofu, etc.). Pupils who consumed any of these foods more than twice in the past week were considered to have consumed that food group and one point was recorded. In this study, the total dietary diversity score is 8, we defined DDS ≤ 4 as low dietary diversity and DDS > 4 as high dietary diversity.

### 2.5 Covariates

Some important potential covariates are included in the model to adjust the analysis. For example, gender (boy or girl), age (continuous, years), caregivers’ current smoking status (yes, no, quit smoking more than 1 year or quit smoking less than 1 year), breakfast times per week (continuous, times/week), weekly nocturnal sleep time (continuous, hours/day), weight (continuous, kg), BMI (continuous, kg/m^2^), PA time per day (< 30 min, 30 min −1 h, ≥1 h), and dietary intake of vegetable, fruit, meat, coarse grain, milk, egg, fish and soybean (< 1 times/week, 1–2 times/week, 3–5 times/week, > 5 times/week).

### 2.6 Statistical analysis

EpiData version 3.0 (EpiData Association, Odense, Denmark) was used to enter this questionnaire and create a database. Subsequently, the Statistical Package for Social Science (version 25.0, SPSS Inc., USA) and R software (version 4.1.1, R Foundation for Statistical Computing, Austria) were used for all statistical analyses. Mean and standard deviation (*SD*), frequency (*N*), and proportion (%) were used to describe the basic characteristics and dietary intake of the pupils. Crude data were compared using the *Chi*-square test for categorical variables and the *t*-test for numerical variables. The multiple generalized linear regression models were used to explore the association between dietary intake and growth and development of pupils, adjusting for multiple covariates in four models. Model 1 adjusted for gender, age, caregivers’ current smoking status, breakfast, weekly nocturnal sleep time, physical activity time and dietary intake (vegetable, fruit, meat, coarse grain, milk, egg, fish and soybean). Model 2 adjusted for variables in Model 1 and weight. Model 3 adjusted for the variables in Model 1 and BMI. Model 4 adjusted for the variables in Model 1 along with weight and BMI. Differences were considered statistically significant when *p* values were less than 0.05.

## 3 Results

### 3.1 Population characteristics

The characteristics of the study population stratified by gender are shown in Table 1. Among the 592 pupils, the proportion of boys (48.1%) was lower than that of girls (51.9%). The average age of all pupils was 9.71 years, with boys being 9.72 years and girls being 9.70 years old on average. In other aspects, the mean weight, BMI, sitting height, stature-sitting height index, waist circumference, chest circumference, standing long jump, and grip strength of boys were significantly higher than those of the girls, while body height, body fat rate, and fat weight showed the opposite trend.

**Table 1:** Main characteristics of pupils^a^.

Characteristic	Total (*n* = 592)	Gender		*p*-value^b^
		Boy (*n* = 285)	Girl (*n* = 307)	
Age, years	9.71 (1.34)	9.72 (1.32)	9.70 (1.37)	0.912
Body height, cm	138.75 (9.86)	138.61 (9.52)	138.89 (10.18)	0.727
Weight, kg	33.13 (9.63)	33.82 (9.97)	32.48 (9.27)	0.092
BMI, kg/m^2^	16.93 (3.34)	17.34 (3.66)	16.56 (2.96)	0.005
Sitting height, cm	74.65 (5.23)	74.68 (5.08)	74.61 (5.36)	0.870
Stature-sitting height index	53.83 (1.61)	53.91 (1.64)	53.75 (1.59)	0.235
Body fat rate, %	14.13 (7.98)	12.68 (7.82)	15.47 (7.90)	< 0.001
Fat weight, kg	5.28 (4.47)	4.92 (4.41)	5.62 (4.50)	0.057
Waist, cm	59.41 (8.41)	61.22 (9.32)	57.63 (7.15)	< 0.001
Chest, cm	66.31 (7.75)	67.45 (7.77)	65.25 (7.59)	0.001
Standing long jump, cm	130.70 (24.06)	138.11 (25.19)	122.82 (20.74)	< 0.001
Grip strength, kg				
Left hand	13.16 (4.20)	14.13 (4.22)	12.26 (3.98)	< 0.001
Right hand	14.30 (4.44)	15.28 (4.58)	13.39 (4.10)	< 0.001
Weekly nocturnal sleep time, hours/day	9.56 (0.89)	9.54 (0.91)	9.57 (0.86)	0.688
Breakfast, times/week	6.15 (1.52)	6.09 (1.64)	6.21 (1.40)	0.313
Physical activity time per day				
< 30 min	124 (20.9)	57 (20.0)	67 (21.8)	0.016
30 min–1 h	359 (60.6)	162 (56.8)	197 (64.2)	
≥ 1 h	109 (18.4)	66 (23.2)	43 (14.0)	
Caregivers’ current smoking status				
Yes	191 (32.2)	95 (33.3)	96 (31.2)	0.877
No	364 (61.4)	173 (60.7)	191 (62.2)	
Quit smoking more than 1 year	31 (5.2)	15 (5.2)	16 (5.2)	
Quit smoking less than 1 year	6 (1.0)	2 (0.7)	4 (1.3)	

^a^Data were presented as Mean (SD) for continuous variables or n (%) for categorical variables.

^bp-values were calculated from t test for numerical variables and Chi-square test for categorical variables.^

### 3.2 Dietary intake and dietary diversity

Table 2 presents a summary of the dietary intake distribution among students of different genders. The average DDS of pupils was 4.05. There was no gender difference in dietary diversity (*p* = 0.930), but girls had greater DDS than boys (4.05 vs. 4.04). The proportion of pupils with low DDS was 59.1% (350/592, with 167 boys and 183 girls). Additionally, only a small proportion of pupils consumed fish (> 5 times/week 6.6%) and soybean (> 5 times/week 6.9%). There were statistically significant differences in fruit intake (*p* = 0.002), meat intake (*p* = 0.036), and milk intake (*p* = 0.001) among pupils of different genders.

**Table 2:** Distributions of dietary intake in pupils^a^.

Dietary intake	Total (*n* = 592)	Gender		*p*-value^b^
		Boy (*n* = 285)	Girl (*n* = 307)	
Dietary diversity score	4.05 (1.87)	4.04 (1.91)	4.05 (1.84)	0.930
Dietary diversity				
Low dietary diversity	350 (59.1)	167 (58.6)	183 (59.6)	0.802
High dietary diversity	242 (40.9)	118 (41.4)	124 (40.4)	
Vegetable intake, times/week				
< 1	35 (5.9)	22 (7.7)	13 (4.2)	0.134
1–2	94 (15.9)	44 (15.4)	50 (16.3)	
3–5	169 (28.5)	72 (25.3)	97 (31.6)	
> 5	294 (49.7)	147 (51.6)	147 (47.9)	
Fruit intake, times/week				
< 1	58 (9.8)	35 (12.3)	23 (7.5)	0.002
1–2	124 (20.9)	58 (20.4)	66 (21.5)	
3–5	240 (40.5)	96 (33.7)	144 (46.9)	
> 5	170 (28.7)	96 (33.7)	74 (24.1)	
Meat intake, times/week				
< 1	142 (24.0)	75 (26.3)	67 (21.8)	0.036
1–2	204 (34.5)	87 (30.5)	117 (38.1)	
3–5	156 (26.4)	70 (24.6)	86 (28.0)	
> 5	90 (15.2)	53 (18.6)	37 (12.1)	
Coarse grain intake, times/week				
< 1	113 (19.1)	56 (19.6)	57 (18.6)	0.149
1–2	243 (41.0)	104 (36.5)	139 (45.3)	
3–5	162 (27.4)	84 (29.5)	78 (25.4)	
> 5	74 (12.5)	41 (14.4)	33 (10.7)	
Milk intake, times/week				
< 1	54 (9.1)	31 (10.9)	23 (7.5)	0.001
1–2	134 (22.6)	77 (27.0)	57 (18.6)	
3–5	306 (51.7)	123 (43.2)	183 (59.6)	
> 5	98(16.6)	54 (18.9)	44 (14.3)	
Egg intake, times/week				
< 1	91 (15.4)	46 (16.1)	45 (14.7)	0.125
1–2	234 (39.5)	103 (36.1)	131 (42.7)	
3–5	182 (30.7)	86 (30.2)	96 (31.3)	
> 5	85 (14.4)	50 (17.5)	35 (11.4)	
Fish intake, times/week				
< 1	181 (30.6)	88 (30.9)	93 (30.3)	0.371
1–2	245 (41.4)	110 (38.6)	135 (40.0)	
3–5	127 (21.5)	64 (22.5)	63 (20.5)	
> 5	39 (6.6)	23 (8.1)	16 (5.2)	
Soybean intake, times/week				
< 1	144 (24.3)	73 (25.6)	71 (23.1)	0.720
1–2	245 (41.4)	120 (42.1)	125 (40.7)	
3–5	162 (27.4)	72 (25.3)	90 (29.3)	
> 5	41 (6.9)	20 (7.0)	21 (6.8)	

^a^Data were presented as Mean (SD) for continuous variables or *n* (%) for categorical variables.

^b^*p*-values were calculated from t test for numerical variables and Chi-square test for categorical variables.Data were presented as *β* (95% CI).

### 3.3 Association between dietary diversity score and growth and development

The associations between DDS and growth and development measurement indicators are depicted in Table 3 through generalized linear regression analysis. In the fully adjusted model, we observed a negative association between standing long jump and DDS for all pupils (*β* = −6.735, 95% *CI*: −12.064, −1.406). In addition, body fat rate (*β* = −2.650, 95% *CI*: −4.794, −0.507), fat weight (*β* = −1.283, 95% *CI*: −2.286, −0.280), and chest circumference (*β* = −1.456, 95% *CI*: −2.789, −0.123) were also negatively correlated with DDS in boys. However, none of the correlations between DDS and the other indicators were statistically significant, so we conducted separate analyses for the intake of each food group.

**Table 3:** Association between dietary diversity score and growth and development.

Growth and development	High / Low dietary diversity					
	Total (*n* = 242/350)		Boy (*n* = 118/167)		Girl (*n* = 124/183)	
	*β* (95% *CI*)	*p*-value	*β* (95% *CI*)	*p*-Value	*β* (95% *CI*)	*p*-value
Body height, cm						
Model 1	0.513 (−1.392, 2.417)	0.598	1.742 (−1.163, 4.647)	0.240	0.201 (−2.396, 2.799)	0.879
Model 2	0.878 (−0.580, 2.336)	0.238	1.799 (−0.409, 4.007)	0.110	0.994 (−0.981, 2.969)	0.324
Model 3	0.803 (−0.994, 2.600)	0.381	2.029 (−0.684, 4.742)	0.143	0.629 (−1.837, 3.095)	0.617
Model 4	0.234 (−0.351, 0.819)	0.433	0.368 (−0.466, 1.202)	0.387	0.607 (−0.225, 1.439)	0.153
Weight, kg						
Model 1	−0.727 (−3.170, 1.716)	0.560	−0.122 (−4.148, 3.903)	0.952	−1.432 (−4.484, 1.621)	0.358
Model 3	0.308 (−0.611, 1.227)	0.512	0.917 (−0.508, 2.342)	0.207	0.012 (−1.218, 1.242)	0.985
BMI, kg/m^2^						
Model 1	−0.442 (−1.408, 0.524)	0.370	−0.449 (−2.074, 1.176)	0.588	−0.602 (−1.768, 0.564)	0.311
Model 2	−0.175 (−0.538, 0.188)	0.345	−0.402 (−0.977, 0.173)	0.170	−0.102 (−0.571, 0.368)	0.672
Sitting height, cm						
Model 1	−0.160 (−1.300, 0.981)	0.784	0.306 (−1.452, 2.064)	0.733	−0.190 (−1.742, 1.362)	0.810
Model 2	0.053 (−0.834, 0.941)	0.906	0.339 (−1.038, 1.716)	0.629	0.284 (−0.896, 1.464)	0.637
Model 3	0.047 (−1.000, 1.094)	0.929	0.502 (−1.106, 2.111)	0.541	0.139 (−1.279, 1.556)	0.848
Model 4	−0.217 (−0.906, 0.473)	0.538	−0.275 (−1.340, 0.789)	0.612	0.128 (−0.808, 1.065)	0.788
Stature-sitting height index						
Model 1	−0.299 (−0.771, 0.173)	0.215	−0.425 (−1.152, 0.302)	0.252	−0.188 (−0.829, 0.454)	0.566
Model 2	−0.287 (−0.758, 0.183)	0.232	−0.424 (−1.149, 0.302)	0.252	−0.155 (−0.794, 0.483)	0.634
Model 3	−0.262 (−0.728, 0.203)	0.269	−0.395 (−1.114, 0.324)	0.282	−0.119 (−0.748, 0.509)	0.709
Model 4	−0.232 (−0.689, 0.225)	0.320	−0.306 (−1.014, 0.402)	0.397	−0.118 (−0.734, 0.497)	0.706
Body fat rate, %						
Model 1	−1.659 (−3.959, 0.641)	0.158	−3.549 (−6.875, −0.223)	0.037	−0.715 (−3.962, 2.532)	0.666
Model 2	−1.164 (−2.754, 0.426)	0.151	−3.480 (−5.924, −1.036)	0.005	0.515 (−1.401, 2.431)	0.598
Model 3	−0.812 (−2.175, 0.552)	0.243	−2.850 (−5.011, −0.690)	0.010	0.795 (−0.621, 2.211)	0.271
Model 4	−0.763 (−2.119, 0.593)	0.270	**−2.650 (−4.794, −0.507)**	**0.015**	0.796 (−0.614, 2.206)	0.268
Fat weight, kg						
Model 1	−0.899 (−2.164, 0.366)	0.164	−1.583 (−3.442, 0.277)	0.095	−0.676 (−2.449, 1.096)	0.454
Model 2	−0.583 (−1.269, 0.103)	0.096	−1.536 (−2.599, −0.473)	0.005	0.066 (−0.731, 0.863)	0.870
Model 3	−0.407 (−1.071, 0.257)	0.230	−1.153 (−2.174, −0.133)	0.027	0.149 (−0.619, 0.917)	0.704
Model 4	−0.465 (−1.107, 0.177)	0.155	**−1.283 (−2.286, −0.280)**	**0.012**	0.146 (−0.561, 0.853)	0.686
Waist, cm						
Model 1	−0.667 (−3.009, 1.676)	0.577	−0.728 (−4.670, 3.213)	0.717	−1.329 (−4.068, 1.410)	0.342
Model 2	−0.063 (−1.237, 1.111)	0.916	−0.621 (−2.350, 1.108)	0.482	−0.257 (−1.769, 1.255)	0.739
Model 3	0.258 (−0.925, 1.441)	0.669	0.250 (−1.476, 1.976)	0.777	−0.185 (−1.797, 1.428)	0.823
Model 4	0.121 (−0.990, 1.232)	0.831	−0.164 (−1.770, 1.442)	0.841	−0.191 (−1.671, 1.290)	0.801
Chest, cm						
Model 1	−0.810 (−2.824, 1.203)	0.430	−1.767 (−4.912, 1.379)	0.271	−0.162 (−2.813, 2.488)	0.904
Model 2	−0.269 (−1.139, 0.600)	0.543	−1.681 (−3.047, −0.314)	0.016	0.971 (−0.123, 2.064)	0.082
Model 3	−0.013 (−1.019, 0.994)	0.980	−1.004 (−2.507, 0.499)	0.191	1.007 (−0.372, 2.387)	0.152
Model 4	−0.191 (−1.045, 0.663)	0.661	**−1.456 (−2.789, −0.123)**	**0.032**	0.999 (−0.086, 2.085)	0.071
Standing long jump, cm						
Model 1	−5.703 (−11.331, −0.076)	0.047	−7.181 (−16.166, 1.804)	0.177	−3.209 (−10.206, 3.787)	0.369
Model 2	−5.954 (−11.519, −0.388)	0.036	−7.244 (−15.983, 1.494)	0.104	−3.286 (−10.290, 3.718)	0.358
Model 3	−6.338 (−11.795, −0.880)	0.023	−7.181 (−16.166, 1.804)	0.117	−3.209 (−10.206, 3.787)	0.369
Model 4	**−6.735 (−12.064, −1.406)**	**0.013**	**−9.187 (−17.500, −0.874)**	**0.030**	−3.632 (−10.452, 3.189)	0.297
Grip strength, kg						
Left hand						
Model 1	0.126 (−0.830, 1.082)	0.797	0.642 (−0.812, 2.096)	0.387	−0.283 (−1.557, 0.991)	0.663
Model 2	0.250 (−0.611, 1.110)	0.569	0.661 (−0.658, 1.979)	0.326	−0.007 (−1.139, 1.124)	0.990
Model 3	0.258 (−0.654, 1.170)	0.579	0.758 (−0.635, 2.151)	0.286	−0.065 (−1.270, 1.139)	0.915
Model 4	0.125 (−0.696, 0.946)	0.766	0.355 (−0.893, 1.604)	0.577	−0.070 (−1.164, 1.023)	0.900
Right hand						
Model 1	0.220 (−0.754, 1.194)	0.658	0.683 (−0.859, 2.224)	0.386	−0.037 (−1.272, 1.198)	0.953
Model 2	0.355 (−0.507, 1.217)	0.419	0.703 (−0.678, 2.084)	0.318	0.257 (−0.809, 1.323)	0.637
Model 3	0.365 (−0.557, 1.286)	0.438	0.814 (−0.653, 2.281)	0.277	0.197 (−0.954, 1.348)	0.737
Model 4	0.222 (−0.595, 1.039)	0.594	0.379 (−0.926, 1.684)	0.569	0.192 (−0.831, 1.214)	0.713

The bold values indicates that the difference is statistically significant.

Data were presented as *β* (95% CI).

Model 1 adjusted for gender, age, caregivers’ smoking, breakfast, weekly nocturnal sleep time, physical activity time and dietary intake (vegetable, fruit, meat, coarse grain, milk, egg, fish and soybean), and except for gender.

Model 2 adjusted for gender, age, caregivers’ smoking, breakfast, weekly nocturnal sleep time, physical activity time, weight and dietary intake (vegetable, fruit, meat, coarse grain, milk, egg, fish and soybean), and except for gender (not applicable to weight).

Model 3 adjusted for gender, age, caregivers’ smoking, breakfast, weekly nocturnal sleep time, physical activity time, BMI and dietary intake (vegetable, fruit, meat, coarse grain, milk, egg, fish and soybean), and except for gender (not applicable to BMI).

Model 4 adjusted for gender, age, caregivers’ smoking, breakfast, weekly nocturnal sleep time, physical activity time, weight, BMI and dietary intake (vegetable, fruit, meat, coarse grain, milk, egg, fish and soybean), and except for gender (not applicable to weight and BMI).

### 3.4 Association between dietary intake and growth and development

**Figure 1:**
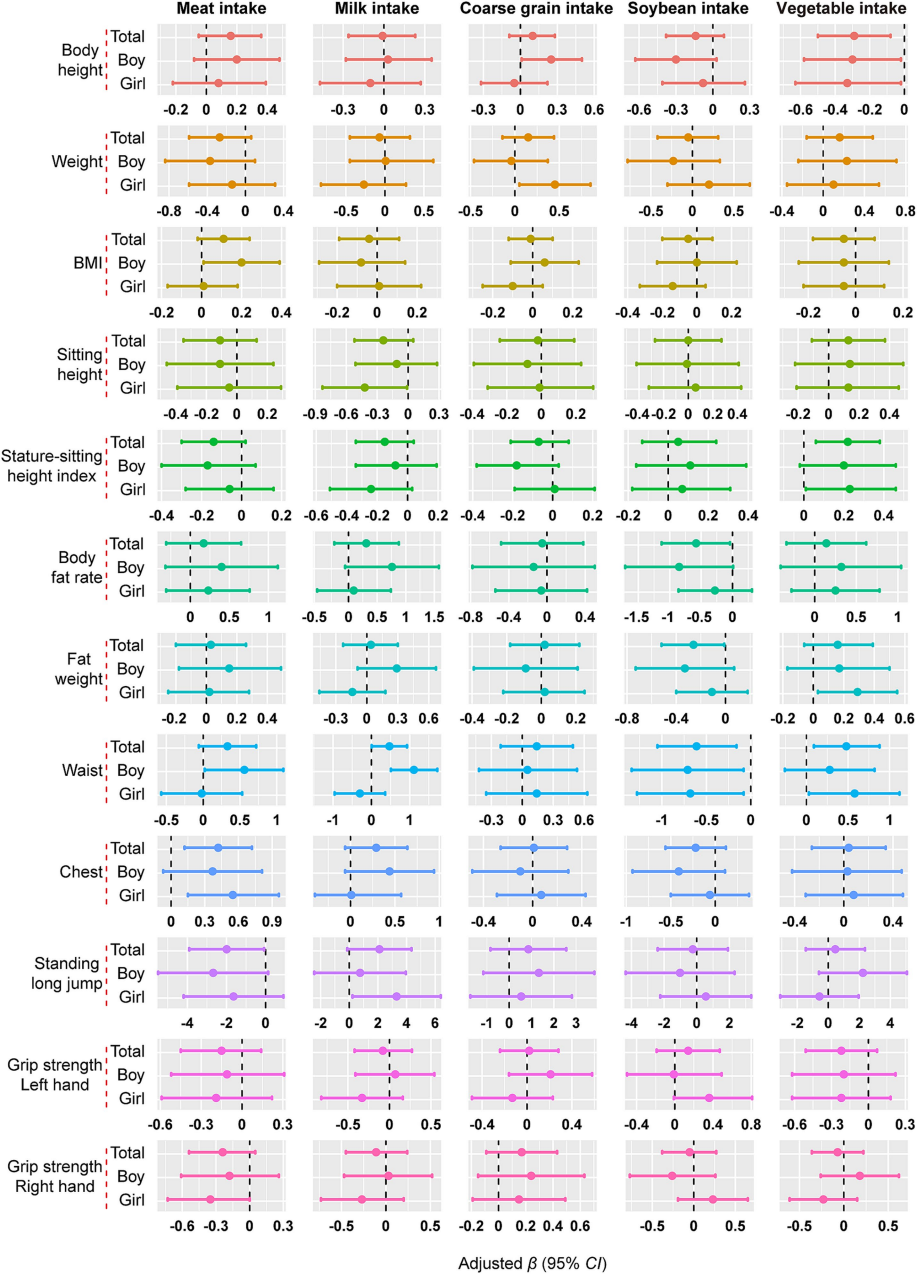
The association between dietary intake and growth and development. Data were presented as effect estimates (*β*) and a 95% confidence interval (95% *CI*). Adjusted for gender, age, caregivers’ current smoking status, breakfast, weekly nocturnal sleep time, physical activity time, weight, BMI and dietary intake (vegetable, fruit, meat, coarse grain, milk, egg, fish and soybean) (not applicable to weight and BMI), and except for the analyzed food group. Gender was excluded when stratified by gender, *p* < 0.05.

Figure 1 illustrates the relationship between various dietary intake and growth and development (fully adjusted model). Among all pupils, meat intake was positively correlated with chest circumference (*β* = 0.420, 95% *CI*: 0.119, 0.721) and negatively correlated with standing long jump (*β* = −1.991, 95% *CI*: −3.902, −0.080); milk intake was positively correlated with waist circumference (*β* = 0.470, 95% *CI*: 0.007, 0.932); soybean intake was negatively correlated with body fat rate (*β* = −0.583, 95% *CI*: −1.125, −0.042), fat weight (*β* = −0.262, 95% *CI*: −0.517, −0.006), and waist circumference (*β* = −0.607, 95% *CI*: −1.050, −0.164); and vegetable intake was negatively correlated with height (*β* = −0.290, 95% *CI*: −0.496, −0.084), and positively correlated with stature-sitting height index and waist circumference (*p* < 0.05) (see Supplementary Tables 1–8 for details). Some of these results were statistically significant in the gender-stratified analyses. For example, meat intake was positively associated with BMI and waist circumference in boys, and with chest circumference in girls. Milk intake was positively correlated with waist circumference in boys and standing long jump in girls, and coarse grain intake was positively correlated with height in boys and weight in girls. Soybean intake was inversely associated with waist in both boys and girls. Vegetable intake was negatively associated with height in both boys and girls.

### 3.5 Association between the frequency of meat, milk, coarse grain, soybean and vegetable intake and growth and development

**Figure 2:**
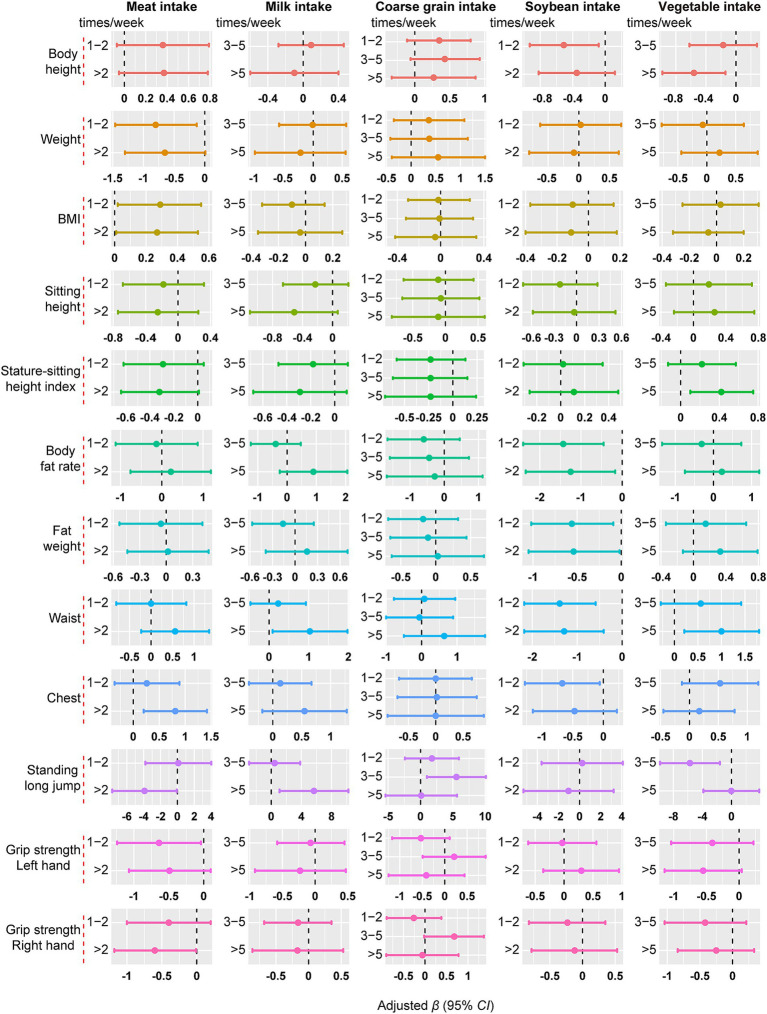
The association between the frequency of meat, milk, coarse grain, soybean and vegetable intake and growth and development. Data were presented as effect estimates (*β*) and a 95% confidence interval (95% *CI*). Adjusted for gender, age, caregivers’ current smoking status, breakfast, weekly nocturnal sleep time, physical activity time, weight, BMI and dietary intake (vegetable, fruit, meat, coarse grain, milk, egg, fish and soybean) (not applicable to weight and BMI), and except for the analyzed food group, *p* < 0.05.

Figure 2 illustrates the correlation between the frequency of meat, milk, coarse grain, soybean and vegetable intake with all pupils’ growth and development. Compared with eating meat less than once a week, eating meat more than twice a week was positively associated with BMI (*β* = 0.266, 95% *CI*: 0.007, 0.525) and chest circumference (*β* = 0.808, 95% *CI*: 0.197, 1.419) and negatively associated with standing long jump (*β* = −3.897, 95% *CI*: −7.725, −0.069) in pupils. In addition, milk intake more than five times per week was positively associated with waist circumference (*β* = 1.030, 95% *CI*: 0.085, 1.975) and standing long jump (*β* = 5.657, 95% *CI*: 1.098, 10.215) of pupils. Soybean intake more than once a week was negatively associated with body fat rate, fat weight, and waist circumference of pupils (*p* < 0.05). Vegetable intake more than five times a week was negatively associated with height and positively associated with waist circumference of pupils (*p* < 0.05). The relationship between dietary intake frequency and growth and development for boys and girls is shown in Supplementary Tables 9–18.

## 4 Discussion

This cross-sectional study investigated the relationships between dietary intake and the growth and development of pupils in Hubei, China. The results showed that, after adjusting for confounding factors, the DDS was negatively correlated with standing long jump of all pupils and body fat rate, fat weight, chest circumference of boys. Furthermore, there were significant correlations between the intake of meat, soybean, milk, coarse grain, and vegetable and growth and development indicators, and the effects of higher frequency of meat, milk, coarse grain and soybean intake on the growth and development of pupils were more obvious.

Weight, height, and BMI are crucial indicators of children’s developmental stage. Among children aged 7 to 12 years, girls had a higher average height compared to boys, while boys had a higher average weight compared to girls. However, these measurements had no statistically significant differences between the genders. This finding does not align with the results of previous studies (18, 19). This difference could be attributed to girls reaching puberty earlier than boys, which could explain their higher height (18). In addition, boys are traditionally expected to be powerful and girls to be slender in rural Chinese viewpoints. Therefore, girls and their families may restrict their diet (20).

Our study showed that the average DDS of pupils was 4.05, with 59.1% having a low DDS. Compared with the results of other studies (12, 21, 22), it was lower than the average DDS value reported by Meng et al. (6.11), Hu et al. (4.67) and Bi et al. (5.77). Diverse study populations and variations in DDS measurement could account for the discrepancies in DDS results between studies (22). Meng et al. (21) collected dietary data over a three-day period, investigated children aged 3–17 years, and utilized a DDS calculation method that did not specify a minimum consumption for any food category. Hu et al. and Bi et al. focused on preschool children in the poor areas of ethnic minorities, collecting dietary information within a 24-h period (12, 22). However, this study collected the frequency of food intake within a week, and the subjects also came from impoverished rural areas.

Body fat rate, fat weight, chest circumference are considered as important indicators of obesity. To the best of our knowledge, no previous studies found a direct association between DDS and body fat rate, fat weight, and chest circumference. However, a study conducted in Iran suggested that DDS was associated with a lower risk of obesity and abdominal adiposity (23). Another study demonstrated a negative correlation between DDS and Waist-to-Hip Ratio (WHR), indicating that dietary diversity may improve overall health by influencing fat distribution in the body (24). A diverse diet can provide essential micronutrients such as fiber (25), which aids in increasing satiety and reducing calorie intake. Standing long jump can reflect the body’s lower limb muscle strength. Our study found a negative association between DDS and standing long jump in pupils. This finding contradicts common expectations. It may be because there is currently no international consensus on the methods for measuring DDS (including food group classification, minimum quantity requirements for food consumption, and reference times) (22), and the specific level of food was not considered in our design.

It is well known that increasing the intakes of meat not only increases protein intake but also raises total energy intake, leading to fat accumulation in the body and having adverse effects on body composition and energy regulation (26). A systematic review and meta-analysis investigating the relationship between red and processed meat consumption and obesity revealed a direct correlation between the intake of these meats and the risk of obesity, higher BMI, and increased waist circumference (27). A cross-sectional study based on the Chinese population found that there is a positive correlation between red meat consumption and BMI, and eating more red meat may increase BMI (28). Similarly, a multiethnic cohort study indicated that diets high in processed and red meats may contribute to obesity and the accumulation of body fat (29). These are similar to our research results. In our study, meat intake were positively correlated with boys’ BMI and waist circumference, girls’ chest circumference. However, meat intake was negatively correlated with standing long jump of pupils. We speculate that this may be due to the fact that most common consumed meat by pupils in our study area is pork. Pork has a high fat content and is generally cooked with high oil, which is not conducive to increasing muscle mass.

Milk and dairy consumption can provide a rich source of protein, fat, calcium, and vitamin D to the human body (30), all of which are essential elements of a healthy and balanced diet. At present, there is no consistent conclusion regarding the relationship between milk and dairy product intake and indicators of overweight and obesity. A meta-analysis of 37 randomized controlled trials found that increased dairy intake was associated with reductions in body weight, body fat, and waist circumference (31). In another cross-sectional study, it was observed that increased consumption of milk and dairy products led to an increase in waist circumference, but not related to BMI (32). In this study, milk intake was positively correlated with waist circumference. This may be because certain components of milk, such as lactose and saturated fat, may cause metabolic changes in some populations, further affecting fat distribution. Furthermore, we found a positive association between standing long jump performance and milk intake, which is consistent with the conclusion of zhang et al. (33). Vitamin D in milk can enhance the body’s absorption of calcium, thereby promoting bone growth, maintaining bone density and muscle strength (33).

The traditional classification of grains in China generally includes rice and its products, wheat products, and other cereals. Other grains are typically treated as coarse grains, similar to the Western definition of whole grains (34). Some previous studies have shown that a higher intake of whole grains is associated with a lower risk of overweight or obesity (35–37). To our surprise, this study found a positive correlation between the intake of coarse grain and weight. We suspect that this may be because Chinese people add other ingredients or even fry them to improve the appearance and taste of whole grains, which increases fat content and contributes to obesity. In addition, we also found a certain positive correlation between the intake of coarse grain and height. The dietary fiber in coarse grain helps regulate intestinal function, promote digestion and absorption, improve the body’s utilization of nutrients, thereby supporting bone growth and development, and promoting height growth (38).

In our study, soybean intake was negatively correlated with body fat rate, fat weight, and waist circumference of pupils. This finding is inconsistent with the results of another study conducted in children and adolescents (39). However, several previous studies have found that soybean supplements can help control body weight and prevent obesity (40, 41). Soybean foods are rich in protein and soy isoflavones (42), which can limit or reduce fat accumulation in the body, resulting in weight loss (43). Moreover, soybeans have high dietary fiber content, which promotes satiety and reduces energy intake (44).

Vegetables are widely believed to be able to control weight due to their low energy density, lowest dietary fat content, and rich vitamin dietary fiber (45). However, the findings on the association between vegetable consumption and body weight and waist circumference are inconsistent. A systematic review of a cohort study found a negative correlation between vegetable intake and weight related outcomes (46). Another prospective cohort study found that increased intake of starchy vegetables was positively associated with weight gain and increased intake of non-starchy vegetables was negatively associated with weight gain (47). This study found a positive correlation between vegetable intake and waist circumference, possibly due to local pupils consuming more starchy vegetables such as peas, corn, and potatoes. Furthermore, contrary to previous research findings (48, 49), we found a negative correlation between vegetable intake and height in primary school students. Generally speaking, vegetables are considered an important source of calcium, but vegetables such as spinach, cabbage, and broccoli contain phytic acid and calcium oxalate, which combine with calcium in food to form insoluble calcium salts, making it difficult for the body to absorb calcium (50). Over time, this may affect bone development. The vegetables intake by the subjects of this study are likely to be mostly of these types. Therefore, more specific investigations on the types of vegetables consumed are needed in the follow-up to further verify this result.

This study examined the association between dietary diversity, dietary intake of each food group, and growth and development in pupils while controlling for confounding variables. However, the present study also has several limitations. Firstly, this study is a cross-sectional design and its causal relationship cannot be determined. Therefore, further studies with a prospective design are required to confirm our findings. Secondly, we collected weekly food intake frequency over the past 3 months, which may be susceptible to recall bias and may not accurately reflect participants’ dietary intake. In the future, we will compare the situation before and after specific dietary intake based on foods’ amount. Thirdly, there might have been bias in the selection process because the study participants were from rural areas.

## 5 Conclusion

In conclusion, this study demonstrates that DDS was negatively associated with standing long jump in all pupils and body fat rate, fat weight, and waist circumference in primary school boys. In the food groups studied, the intake of meat, milk, soybean, coarse grain, and vegetable was correlated with growth and development indicators such as height, weight, BMI, body fat rate, and waist circumference, and dietary intake should be purposefully controlled. Future studies need to more fully consider specific dietary types and intake levels.

## Data Availability

The datasets presented in this article are not readily available because these data contain information that may compromise the privacy of study participants but are available from the corresponding author on reasonable request. Requests to access the datasets should be directed to Yu Lu, y.lu@hbmu.edu.cn.

## References

[1] Norris SA Frongillo EA Black MM Dong Y Fall C Lampl M. Nutrition in adolescent growth and development. Lancet (London, England). (2022) 399:172–84. doi: 10.1016/S0140-6736(21)01590-7, PMID: 34856190

[2] Rojo M Solano S Lacruz T Baile JI Blanco M Graell M. Linking psychosocial stress events, psychological disorders and childhood obesity. Children (Basel, Switzerland). (2021) 8:211. doi: 10.3390/children8030211 33802090 PMC8000555

[3] Wang C Chan JS Ren L Yan JH. Obesity reduces cognitive and motor functions across the lifespan. Neural Plast. (2016) 2016:1–13. doi: 10.1155/2016/2473081, PMID: 26881095 PMC4737453

[4] Aras M Tchang BG Pape J. Obesity and diabetes. Nurs Clin North Am. (2021) 56:527–41. doi: 10.1016/j.cnur.2021.07.008, PMID: 34749892

[5] Chung ST Krenek A Magge SN. Childhood obesity and cardiovascular disease risk. Curr Atheroscler Rep. (2023) 25:405–15. doi: 10.1007/s11883-023-01111-4, PMID: 37256483 PMC10230147

[6] Khadilkar AV Chiplonkar SA Kajale NA Ekbote VH Parathasarathi L Padidela R. Impact of dietary nutrient intake and physical activity on body composition and growth in Indian children. Pediatr Res. (2018) 83:843–50. doi: 10.1038/pr.2017.322, PMID: 29278646

[7] Xiong T Wu Y Hu J Xu S Li Y Kong B. Associations between high protein intake, linear growth, and stunting in children and adolescents: a cross-sectional study. Nutrients. (2023) 15:4821. doi: 10.3390/nu15224821, PMID: 38004215 PMC10675685

[8] Morales-Suárez-Varela M Rubio-López N Ruso C Llopis-Gonzalez A Ruiz-Rojo E Redondo M. Anthropometric status and nutritional intake in children (6-9 years) in Valencia (Spain): the ANIVA study. Int J Environ Res Public Health. (2015) 12:16082–95. doi: 10.3390/ijerph121215045, PMID: 26694443 PMC4690981

[9] Golpour-Hamedani S Rafie N Pourmasoumi M Saneei P Safavi SM. The association between dietary diversity score and general and abdominal obesity in Iranian children and adolescents. BMC Endocr Disord. (2020) 20:181. doi: 10.1186/s12902-020-00662-w, PMID: 33308202 PMC7733278

[10] Molani Gol R Kheirouri S Alizadeh M. Association of Dietary Diversity with Growth Outcomes in infants and children aged under 5 years: a systematic review. J Nutr Educ Behav. (2022) 54:65–83. doi: 10.1016/j.jneb.2021.08.016, PMID: 35000681

[11] Jafari M Izadi A Dehghan P Mojtahedi SY. Dietary diversities score and anthropometric characteristics in Iranian elementary school children. Eur J Transl Myol. (2019) 29:8339. doi: 10.4081/ejtm.2019.8339, PMID: 31579479 PMC6767834

[12] Hu B Tang S Wang Z Chen Y Chen X Zhao Q. Dietary diversity is associated with nutrient adequacy, blood biomarkers and anthropometric status among preschool children in poor ethnic minority area of Northwest China. Front Nutr. (2022) 9:948555. doi: 10.3389/fnut.2022.948555, PMID: 36505258 PMC9729537

[13] Xu Q Zhong C Tan T Lin L Yang H Xu Z. The influence of dietary diversity on anthropometric status among young children ages 12 and 24 months in Wuhan, China. Matern Child Nutr. (2023) 20:e13563. doi: 10.1111/mcn.13563 37734736 PMC10750002

[14] Group CSPaHR. Chinese students’ physical fitness and Health Research report 2010. Beijing: High Education Publication (2015). 693 p p.

[15] Group NSPFaHS. National student physical fitness and health survey handbook. Beijing: Higher Education Press (2014).

[16] Lv W Fu J Zhao G He Z Sun S Huang T. A cohort study of factors influencing the physical fitness of preschool children: a decision tree analysis. Front Public Health. (2023) 11:1184756. doi: 10.3389/fpubh.2023.1184756, PMID: 38074715 PMC10701283

[17] Jiang W Mo M Li M Wang S Muyiduli X Shao B. The relationship of dietary diversity score with depression and anxiety among prenatal and post-partum women. J Obstet Gynaecol Res. (2018) 44:1929–36. doi: 10.1111/jog.13728, PMID: 30051541

[18] Sathiadas MG Antonyraja A Viswalingam A Thangaraja K Wickramasinghe VP. Nutritional status of school children living in northern part of Sri Lanka. BMC Pediatr. (2021) 21:43. doi: 10.1186/s12887-021-02501-w, PMID: 33468077 PMC7814636

[19] Goon DT Toriola AL Shaw BS. Stature and body mass of Nigerian children aged 9-12 years. Minerva Pediatr. (2012) 64:325–31. PMID: 22555326

[20] Zong XN Li H. Construction of a new growth references for China based on urban Chinese children: comparison with the WHO growth standards. PLoS One. (2013) 8:e59569. doi: 10.1371/journal.pone.0059569, PMID: 23527219 PMC3602372

[21] Meng L Wang Y Li T Loo-Bouwman CAV Zhang Y Man-Yau SI. Dietary Diversity and Food Variety in Chinese Children Aged 3^−^17 Years: Are They Negatively Associated with Dietary Micronutrient Inadequacy? Nutrients. (2018) 10:1674. doi: 10.3390/nu10111674 30400573 PMC6267553

[22] Bi J Liu C Li S He Z Chen K Luo R. Dietary diversity among preschoolers: a cross-sectional study in poor, rural, and ethnic minority areas of central South China. Nutrients. (2019) 11:558. doi: 10.3390/nu11030558, PMID: 30845662 PMC6471221

[23] Azadbakht L Esmaillzadeh A. Dietary diversity score is related to obesity and abdominal adiposity among Iranian female youth. Public Health Nutr. (2011) 14:62–9. doi: 10.1017/S1368980010000522, PMID: 20353617

[24] Nachvak SM Abdollahzad H Mostafai R Moradi S Pasdar Y Rezaei M. Dietary diversity score and its related factors among employees of Kermanshah University of Medical Sciences. Clinic Nutr Res. (2017) 6:247–55. doi: 10.7762/cnr.2017.6.4.247, PMID: 29124045 PMC5665746

[25] Wang Z Chen Y Tang S Chen S Gong S Jiang X. Dietary diversity and nutrient intake of Han and Dongxiang smallholder farmers in poverty areas of Northwest China. Nutrients. (2021) 13:3908. doi: 10.3390/nu13113908, PMID: 34836163 PMC8621596

[26] Bizzozero-Peroni B Martínez-Vizcaíno V Garrido-Miguel M Fernández-Rodríguez R Torres-Costoso A Ferri-Morales A. The association between meat consumption and muscle strength index in young adults: the mediating role of total protein intake and lean mass percentage. Eur J Nutr. (2023) 62:673–83. doi: 10.1007/s00394-022-03014-7, PMID: 36184663

[27] Rouhani MH Salehi-Abargouei A Surkan PJ Azadbakht L. Is there a relationship between red or processed meat intake and obesity? A systematic review and meta-analysis of observational studies. Obes Rev. (2014) 15:740–8. doi: 10.1111/obr.12172, PMID: 24815945

[28] Wang W Qiu L Sa R Dang S Liu F Xiao X. Effect of socioeconomic characteristics and lifestyle on BMI distribution in the Chinese population: a population-based cross-sectional study. BMC Public Health. (2021) 21:1369. doi: 10.1186/s12889-021-11405-4, PMID: 34246224 PMC8272370

[29] Chai W Morimoto Y Cooney RV Franke AA Shvetsov YB le L. Dietary red and processed meat intake and markers of adiposity and inflammation: the multiethnic cohort study. J Am Coll Nutr. (2017) 36:378–85. doi: 10.1080/07315724.2017.1318317, PMID: 28628401 PMC5540319

[30] Clark DC Cifelli CJ Pikosky MA. Growth and development of preschool children (12-60 months): a review of the effect of dairy intake. Nutrients. (2020) 12:3556. doi: 10.3390/nu12113556, PMID: 33233555 PMC7699766

[31] Geng T Qi L Huang T. Effects of dairy products consumption on body weight and body composition among adults: an updated Meta-analysis of 37 randomized control trials. Mol Nutr Food Res. (2018) 62:1700410. doi: 10.1002/mnfr.201700410, PMID: 29058378

[32] Esen SA Esen İ Açikgöz Y. Nutritional health in premenopausal women: a cross-sectional study from Turkey. Int J Prev Med. (2022) 13:32. doi: 10.4103/ijpvm.IJPVM_141_20, PMID: 35392308 PMC8980836

[33] Zhang X Mao C Tan Y Lu Z Li Z Zhang L. Association between dietary patterns and physical fitness among Chinese children and adolescents in Shaanxi Province. Nutrients. (2022) 14:3677. doi: 10.3390/nu14183677, PMID: 36145061 PMC9503495

[34] Huang Q Hao L Wang L Jiang H Li W Wang S. Differential associations of intakes of whole grains and coarse grains with risks of Cardiometabolic factors among adults in China. Nutrients. (2022) 14:2109. doi: 10.3390/nu14102109, PMID: 35631250 PMC9145902

[35] Jakobsen DD Brader L Bruun JM. Association between food, beverages and overweight/obesity in children and adolescents-a systematic review and Meta-analysis of observational studies. Nutrients. (2023) 15:764. doi: 10.3390/nu15030764 36771470 PMC9920526

[36] Bradlee ML Singer MR Qureshi MM Moore LL. Food group intake and central obesity among children and adolescents in the third National Health and nutrition examination survey (NHANES III). Public Health Nutr. (2010) 13:797–805. doi: 10.1017/S1368980009991546, PMID: 19772691

[37] Choumenkovitch SF McKeown NM Tovar A Hyatt RR Kraak VI Hastings AV. Whole grain consumption is inversely associated with BMI Z-score in rural school-aged children. Public Health Nutr. (2013) 16:212–8. doi: 10.1017/S1368980012003527, PMID: 22894825 PMC10271384

[38] Han X Ma Y Ding S Fang J Liu G. Regulation of dietary fiber on intestinal microorganisms and its effects on animal health. Anim Nutr. (2023) 14:356–69. doi: 10.1016/j.aninu.2023.06.004, PMID: 37635930 PMC10448034

[39] Wang X He T Xu S Li H Wu M Lin Z. Soy food intake associated with obesity and hypertension in children and adolescents in Guangzhou, southern China. Nutrients. (2022) 14:425. doi: 10.3390/nu14030425, PMID: 35276781 PMC8839714

[40] Zhu J Zhao Q Qiu Y Zhang Y Cui S Yu Y. Soy Isoflavones intake and obesity in Chinese adults: a cross-sectional study in Shanghai, China. Nutrients. (2021) 13:2715. doi: 10.3390/nu13082715, PMID: 34444874 PMC8399780

[41] Heng WK Choo JY Ng YP Loh KS Chua YH. Effect of soy-based meal replacement on weight loss: a systematic review and meta-analyses protocol. Nutr Health. (2022) 28:489–93. doi: 10.1177/02601060221089105, PMID: 35404174

[42] Tang S Du Y Oh C No J. Effects of soy foods in postmenopausal women: a focus on Osteosarcopenia and obesity. J Obes Metab Syndr. (2020) 29:180–7. doi: 10.7570/jomes20006, PMID: 32843586 PMC7539339

[43] Mu Y Kou T Wei B Lu X Liu J Tian H. Soy products ameliorate obesity-related anthropometric indicators in overweight or obese Asian and non-menopausal women: a Meta-analysis of randomized controlled trials. Nutrients. (2019) 11:2790. doi: 10.3390/nu11112790, PMID: 31731772 PMC6893485

[44] Tucker LA. Legume intake, body weight, and abdominal adiposity: 10-year weight change and cross-sectional results in 15,185 U.S. Adults Nutr. (2023) 15:460. doi: 10.3390/nu15020460, PMID: 36678331 PMC9864712

[45] Li SY Leung JCS Lu ZH Kwok TCY. Quantity and variety of fruit and vegetable intake with changes in measures of adiposity among community-dwelling Chinese older adults. Nutrients. (2023) 15:4096. doi: 10.3390/nu15194096, PMID: 37836380 PMC10574446

[46] Nour M Lutze SA Grech A Allman-Farinelli M. The relationship between vegetable intake and weight outcomes: a systematic review of cohort studies. Nutrients. (2018) 10:1626. doi: 10.3390/nu10111626, PMID: 30400139 PMC6266069

[47] Wan Y Tobias DK Dennis KK Guasch-Ferré M Sun Q Rimm EB. Association between changes in carbohydrate intake and long term weight changes: prospective cohort study. BMJ (Clinical research ed). (2023) 382:e073939. doi: 10.1136/bmj-2022-073939, PMID: 37758268 PMC10523278

[48] Rosário R Agostinis-Sobrinho C Padrão P Lopes O Moreira P. The relationship between height and fruit/vegetable intakes in adults: a nationwide cross-sectional study. Nutr Health. (2024) 30:235–41. doi: 10.1177/02601060221108152, PMID: 35702038 PMC11141095

[49] Parvin T Endres K Hasan MT Uddin IM Bhuyian MSI Zohura F. Low fruit and vegetable consumption associated with linear growth faltering among children in urban Bangladesh. Am J Trop Med Hyg. (2022) 106:1741–6. doi: 10.4269/ajtmh.21-1124, PMID: 35576951 PMC9209935

[50] Liu N Zeng F Zhang K Tang Z. A community-based cross-sectional study for relationship of frequency of vegetables intake and osteoporosis in a Chinese postmenopausal women sample. BMC Womens Health. (2016) 16:28. doi: 10.1186/s12905-016-0307-5, PMID: 27259804 PMC4891848

