# Supplementary material for: Relationship between dietary intake and growth and development in Chinese pupils
Source: Front Public Health. 2025 Feb 12;13:1454129. doi: 10.3389/fpubh.2025.1454129 (PMC11861545; doi:10.3389/fpubh.2025.1454129)
Supplement: Supplementary file 1 [file Table_1.docx]

**Table S1. Association between** **meat intake and growth and development (*n* = 592)**

| **Growth and development** | **Meat intake, times/week** | | | | | | |
| --- | --- | --- | --- | --- | --- | --- | --- |
|  | **Total (*n* = 592)** | | **< 1 (*n* = 142)** | **1** - **2 (*n* = 204)** | | **> 2 (*n* = 246)** | |
|  | ***β* (95% *CI*)** | ***P*-Value** |  | ***β* (95% *CI*)** | ***P*-Value** | ***β* (95% *CI*)** | ***P*-Value** |
| Body height, cm |  |  |  |  |  |  |  |
| Model 1 | -0.270 (-0.940, 0.401) | 0.431 | reference | -1.147 (-2.533, 0.239) | 0.105 | -0.703 (-2.060, 0.655) | 0.310 |
| Model 2 | -0.257 (-0.769, 0.255) | 0.325 | reference | -0.689 (-1.751, 0.374) | 0.204 | -0.614 (-1.654, .426) | 0.247 |
| Model 3 | -0.338 (-0.970, 0.293) | 0.294 | reference | -1.114 (-2.421, 0.194) | 0.095 | -0.835 (-2.116, 0.447) | 0.202 |
| Model 4 | 0.156 (-0.051, 0.363) | 0.139 | reference | 0.358 (-0.070, 0.785) | 0.101 | 0.365 (-0.054, 0.783) | 0.088 |
| Weight, kg |  |  |  |  |  |  |  |
| Model 1 | -0.025 (-0.887, 0.837) | 0.955 | reference | -0.915 (-2.692, 0.863) | 0.313 | -0.178 (-1.920, 1.564) | 0.841 |
| Model 3 | -0.267 (-0.590, 0.056) | 0.105 | reference | -0.796 (-1.464, -0.127) | 0.020 | -0.648 (-1.304, 0.007) | 0.052 |
| BMI, kg/m^2^ |  |  |  |  |  |  |  |
| Model 1 | 0.103 (-0.237, 0.444) | 0.552 | reference | -0.051 (-0.754, 0.652) | 0.887 | 0.201 (-0.488, 0.890) | 0.568 |
| Model 2 | 0.112 (-0.015, 0.240) | 0.084 | reference | 0.285 (0.020, 0.549) | 0.035 | 0.266 (0.007, 0.525) | 0.044 |
| Sitting height, cm |  |  |  |  |  |  |  |
| Model 1 | -0.291 (-0.692, 0.110) | 0.154 | reference | **-0.886 (-1.716, -0.057)** | **0.036** | -0.707 (-1.520, 0.106) | 0.088 |
| Model 2 | -0.284 (-0.595, 0.028) | 0.074 | reference | -0.618 (-1.264, 0.028) | 0.061 | **-0.655 (-1.287, -0.022)** | **0.043** |
| Model 3 | -0.340 (-0.707, 0.028) | 0.070 | reference | -0.862 (-1.624, -0.101) | **0.026** | **-0.801 (-1.547, -0.055)** | **0.035** |
| Model 4 | -0.110 (-0.352, 0.132) | 0.372 | reference | -0.180 (-0.683, 0.323) | 0.484 | -0.245 (-0.738, 0.248) | 0.330 |
| Stature-sitting height index |  |  |  |  |  |  |  |
| Model 1 | -0.102 (-0.268, 0.063) | 0.226 | reference | -0.213 (-0.557, 0.131) | 0.224 | -0.234 (-0.571, 0.102) | 0.173 |
| Model 2 | -0.102 (-0.267, 0.063) | 0.226 | reference | -0.198 (-0.541, 0.145) | 0.257 | -0.232 (-0.567, 0.104) | 0.176 |
| Model 3 | -0.111 (-0.274, 0.053) | 0.185 | reference | -0.209 (-0.548, 0.130) | 0.227 | -0.251 (-0.583, 0.081) | 0.138 |
| Model 4 | -0.137 (-0.297, 0.024) | 0.095 | reference | -0.288 (-0.622, 0.045) | 0.090 | -0.316 (-0.643, 0.011) | 0.058 |
| Body fat rate, % |  |  |  |  |  |  |  |
| Model 1 | 0.414 (-0.400, 1.229) | 0.319 | reference | -0.095 (-1.772, 1.581) | 0.911 | 0.705 (-0.938, 2.348) | 0.400 |
| Model 2 | 0.431 (-0.132, 0.994) | 0.133 | reference | 0.528 (-0.631, 1.688) | 0.372 | 0.826 (-0.309, 1.962) | 0.154 |
| Model 3 | 0.215 (-0.268, 0.698) | 0.383 | reference | 0.002 (-0.990, 0.994) | 0.996 | 0.319 (-0.654, 1.292) | 0.520 |
| Model 4 | 0.172 (-0.309, 0.654) | 0.483 | reference | -0.126 (-1.116, 0.865) | 0.804 | 0.215 (-0.755, 1.185) | 0.664 |
| Fat weight, kg |  |  |  |  |  |  |  |
| Model 1 | 0.094 (-0.354, 0.541) | 0.681 | reference | -0.268 (-1.190, 0.654) | 0.568 | 0.120 (-0.784, 1.023) | 0.795 |
| Model 2 | 0.105 (-0.138, 0.347) | 0.398 | reference | 0.130 (-0.370, 0.631) | 0.610 | 0.197 (-0.293, 0.687) | 0.431 |
| Model 3 | -0.022 (-0.256, 0.212) | 0.854 | reference | -0.212 (-0.695, 0.272) | 0.391 | -0.105 (-0.579, 0.370) | 0.;665 |
| Model 4 | 0.028 (-0.199, 0.255) | 0.810 | reference | -0.062 (-0.532, 0.407) | 0.795 | 0.017 (-0.442, 0.476) | 0.942 |
| Waist, cm |  |  |  |  |  |  |  |
| Model 1 | 0.427 (-0.398, 1.253) | 0.310 | reference | -0.457 (-2.162, 1.248) | 0.599 | 0.691 (-0.979, 2.362) | 0.417 |
| Model 2 | **0.448 (0.033, 0.863)** | **0.034** | reference | 0.302 (-0.552, 1.157) | 0.488 | **0.839 (0.002, 1.676)** | **0.049** |
| Model 3 | 0.212 (-0.207, 0.630) | 0.321 | reference | -0.351 (-1.211, 0.510) | 0.425 | 0.271 (-0.573, 1.114) | 0.529 |
| Model 4 | 0.330 (-0.063, 0.724) | 0.100 | reference | 0.004 (-0.807, 0.815) | 0.992 | 0.560 (-0.234, 1.354) | 0.167 |
| Chest, cm |  |  |  |  |  |  |  |
| Model 1 | 0.453 (-0.258, 1.164) | 0.212 | reference | -0.290 (-1.756, 1.175) | 0.698 | 0.795 (-0.641, 2.232) | 0.278 |
| Model 2 | **0.471 (0.165, 0.778)** | **0.003** | reference | 0.390 (-0.243, 1.023) | 0.227 | **0.928 (0.308, 1.547)** | **0.003** |
| Model 3 | 0.266 (-0.088, 0.619) | 0.141 | reference | -0.199 (-0.930, 0.533) | 0.595 | 0.432 (-0.285, 1.150) | 0.237 |
| Model 4 | 0.420 (0.119, 0.721) | 0.006 | reference | 0.262 (-0.362, 0.886) | 0.410 | 0.808 (0.197, 1.419) | 0.010 |
| Standing long jump, cm |  |  |  |  |  |  |  |
| Model 1 | **-2.475 (-4.481, -0.469)** | **0.016** | reference | -0.831 (-4.938, 3.277) | 0.692 | **-5.008 (-9.033, -0.982)** | **0.015** |
| Model 2 | **-2.483 (-4.469, -0.497)** | **0.014** | reference | -1.141 (-5.207, 2.926) | 0.582 | **-5.068 (-9.050, -1.086)** | **0.013** |
| Model 3 | **-2.332 (-4.283, -0.381)** | **0.019** | reference | -0.902 (-4.887, 3.082) | 0.657 | **-4.723 (-8.629, -0.817)** | **0.018** |
| Model 4 | -1.991 (-3.902, -0.080) | 0.041 | reference | 0.112 (-3.799, 4.022) | 0.955 | -3.897 (-7.725, -0.069) | 0.046 |
| Handgrip strength, kg |  |  |  |  |  |  |  |
| Left hand |  |  |  |  |  |  |  |
| Model 1 | -0.239 (-0.579, 0.100) | 0.167 | reference | **-1.002 (-1.697, -0.306)** | **0.005** | **-0.708 (-1.390, -0.027)** | **0.042** |
| Model 2 | -0.235 (-0.541, 0.070) | 0.132 | reference | **-0.846 (-1.472, -0.219)** | **0.008** | **-0.678 (-1.291, -0.064)** | **0.030** |
| Model 3 | -0.271 (-0.594, 0.053) | 0.101 | reference | **-0.987 (-1.650, -0.324)** | **0.004** | **-0.768 (-1.418, -0.118)** | **0.021** |
| Model 4 | -0.153 (-0.445, 0.138) | 0.303 | reference | -0.642 (-1.242, -0.043) | 0.036 | -0.488 (-1.074, 0.099) | 0.103 |
| Right hand |  |  |  |  |  |  |  |
| Model 1 | -0.335 (-0.681, 0.011) | 0.057 | reference | **-0.786 (-1.495, -0.077)** | **0.030** | **-0.834 (-1.529, -0.140)** | **0.019** |
| Model 2 | **-0.330 (-0.636, -0.025)** | **0.034** | reference | -0.616 (-1.244, 0.011) | 0.054 | **-0.801 (-1.416, -0.187)** | **0.011** |
| Model 3 | **-0.369 (-0.696, -0.043)** | **0.027** | reference | **-0.769 (-1.440, -0.099)** | **0.024** | **-0.900 (-1.557, -0.243)** | **0.007** |
| Model 4 | -0.244 (-0.535, 0.046) | 0.099 | reference | -0.399 (-0.996, 0.197) | 0.190 | -0.598 (-1.182, -0.014) | 0.045 |

Model 1 adjusted for gender, age, caregivers’ smoking, breakfast, weekly nocturnal sleep time, physical activity time and dietary intake (vegetable, fruit, coarse grain, milk, egg, fish and soybean).

Model 2 adjusted for gender, age, caregivers’ smoking, breakfast, weekly nocturnal sleep time, physical activity time, weight and dietary intake (vegetable, fruit, coarse grain, milk, egg, fish and soybean) (not applicable to weight).

Model 3 adjusted for gender, age, caregivers’ smoking, breakfast, weekly nocturnal sleep time, physical activity time, BMI and dietary intake (vegetable, fruit, coarse grain, milk, egg, fish and soybean) (not applicable to BMI).

Model 4 adjusted for gender, age, caregivers’ smoking, breakfast, weekly nocturnal sleep time, physical activity time, weight, BMI and dietary intake (vegetable, fruit, coarse grain, milk, egg, fish and soybean) (not applicable to weight and BMI).

**Table S2. Association between** **milk intake and growth and development (*n* = 592)**

| **Growth and development** | **Milk intake, times/week** | | | | | | |
| --- | --- | --- | --- | --- | --- | --- | --- |
|  | **Total (*n* = 592)** | | **< 3 (*n* = 188)** | **3** - **5 (*n* = 306)** | | **> 5 (*n* = 98)** | |
|  | ***β* (95% *CI*)** | ***P*-Value** |  | ***β* (95% *CI*)** | ***P*-Value** | ***β* (95% *CI*)** | ***P*-Value** |
| Body height, cm |  |  |  |  |  |  |  |
| Model 1 | -0.414 (-1.201, 0.372) | 0.302 | reference | -0.401 (-1.598, 0.797) | 0.512 | -1.078 (-2.695, 0.538) | 0.191 |
| Model 2 | 0.120 (-0.483, 0.723) | 0.696 | reference | 0.434 (-0.487, 1.354) | 0.356 | 0.049 (-1.194, 1.292) | 0.938 |
| Model 3 | -0.132 (-0.875, 0.612) | 0.728 | reference | 0.060 (-1.074, 1.195) | 0.917 | -0.512 (-2.043, 1.018) | 0.512 |
| Model 4 | -0.012 (-0.255, 0.230) | 0.921 | reference | 0.086 (-0.284, 0.455) | 0.649 | -0.101 (-0.599, 0.397) | 0.692 |
| Weight, kg |  |  |  |  |  |  |  |
| Model 1 | **-1.064 (-2.075, -0.052)** | **0.039** | reference | **-1.665 (-3.201, -0.129)** | **0.034** | **-2.250 (-4.324, -0.177)** | **0.033** |
| Model 3 | -0.065 (-0.445, 0.316) | 0.739 | reference | -0.014 (-0.594, 0.566) | 0.963 | -0.222 (-1.005, 0.560) | 0.577 |
| BMI, kg/m^2^ |  |  |  |  |  |  |  |
| Model 1 | **-0.426 (-0.826, -0.026)** | **0.037** | reference | **-0.705 (-1.313, -0.098)** | **0.023** | \| **-0.866 (-1.686, -0.045)** \| \| --- \| | **0.039** |
| Model 2 | -0.036 (-0.186, 0.114) | 0.638 | reference | -0.095 (-0.324, 0.135) | 0.419 | -0.041 (-0.350, 0.269) | 0.796 |
| Sitting height, cm |  |  |  |  |  |  |  |
| Model 1 | **-0.494 (-0.964, -0.023)** | **0.040** | reference | -0.567 (-1.284, 0.150) | 0.121 | \| **-1.110 (-2.078, -0.142)** \| \| --- \| | **0.025** |
| Model 2 | -0.182 (-0.549, 0.185) | 0.330 | reference | -0.079 (-0.639, 0.481) | 0.782 | -0.450 (-1.206, 0.306) | 0.243 |
| Model 3 | -0.293 (-0.726, 0.139) | 0.184 | reference | -0.236 (-0.897, 0.424) | 0.483 | -0.704 (-1.595, 0.187) | 0.122 |
| Model 4 | -0.238 (-0.522, 0.046) | 0.101 | reference | -0.225 (-0.659, 0.210) | 0.311 | -0.513 (-1.100, 0.074) | 0.086 |
| Stature-sitting height index |  |  |  |  |  |  |  |
| Model 1 | -0.174 (-0.368, 0.020) | 0.079 | reference | -0.233 (-0.530, 0.064) | 0.124 | -0.339 (-0.740, 0.062) | 0.097 |
| Model 2 | -0.157 (-0.352, 0.037) | 0.112 | reference | -0.206 (-0.504, 0.091) | 0.173 | -0.303 (-0.704, 0.098) | 0.139 |
| Model 3 | -0.140 (-0.332, 0.052) | 0.154 | reference | -0.175 (-0.469, 0.119) | 0.243 | -0.268 (-0.664, 0.129) | 0.186 |
| Model 4 | -0.146 (-0.335, 0.042) | 0.128 | reference | -0.176 (-0.465, 0.112) | 0.230 | -0.290 (-0.679, 0.099) | 0.144 |
| Body fat rate, % |  |  |  |  |  |  |  |
| Model 1 | -0.497 (-1.453, 0.458) | 0.308 | reference | **-1.741 (-3.190, -0.293)** | **0.018** | -0.727 (-2.682, 1.229) | 0.466 |
| Model 2 | 0.229 (-0.434, 0.892) | 0.498 | reference | -0.606 (-1.611, 0.399) | 0.237 | 0.807 (-0.549, 2.164) | 0.243 |
| Model 3 | 0.323 (-0.246, 0.892) | 0.266 | reference | -0.386 (-1.247, 0.475) | 0.379 | 0.937 (-0.225, 2.099) | 0.114 |
| Model 4 | 0.312 (-0.253, 0.878) | 0.279 | reference | -0.388 (-1.244, 0.468) | 0.374 | 0.901 (-0.254, 2.056) | 0.126 |
| Fat weight, kg |  |  |  |  |  |  |  |
| Model 1 | -0.452 (-0.977, 0.073) | 0.092 | reference | **-0.950 (-1.747, -0.154)** | **0.019** | -0.851 (-1.926, 0.224) | 0.121 |
| Model 2 | 0.012 (-0.273, 0.298) | 0.932 | reference | -0.225 (-0.659, 0.209) | 0.310 | 0.129 (-0.457, 0.715) | 0.666 |
| Model 3 | 0.025 (-0.251, 0.301) | 0.860 | reference | -0.163 (-0.583, 0.256) | 0.446 | 0.115 (-0.451, 0.681) | 0.690 |
| Model 4 | 0.037 (-0.230, 0.303) | 0.786 | reference | -0.161 (-0.566, 0.245) | 0.437 | 0.157 (-0.390, 0.704) | 0.574 |
| Waist, cm |  |  |  |  |  |  |  |
| Model 1 | -0.449 (-1.417, 0.520) | 0.364 | reference | -1.252 (-2.725, 0.221) | 0.096 | -0.881 (-2.870, 1.108) | 0.385 |
| Model 2 | 0.432 (-0.057, 0.920) | 0.083 | reference | 0.130 (-0.610, 0.871) | 0.730 | 0.987 (-0.012, 1.987) | 0.053 |
| Model 3 | 0.441 (-0.051, 0.933) | 0.079 | reference | 0.224 (-0.523, 0.970) | 0.557 | 0.931 (-0.077, 1.938) | 0.070 |
| Model 4 | 0.470 (0.007, 0.932) | 0.047 | reference | 0.230 (-0.471, 0.930) | 0.521 | 1.030 (0.085, 1.975) | 0.033 |
| Chest, cm |  |  |  |  |  |  |  |
| Model 1 | -0.518 (-1.353, 0.316) | 0.223 | reference | -1.147 (-2.414, 0.119) | 0.076 | \| -1.140 (-2.849, 0.570) \| \| --- \| | 0.191 |
| Model 2 | 0.274 (-0.087, 0.634) | 0.137 | reference | 0.091 (-0.457, 0.640) | 0.744 | 0.534 (-0.206, 1.275) | 0.157 |
| Model 3 | 0.253 (-0.163, 0.669) | 0.234 | reference | 0.126 (-0.509, 0.761) | 0.697 | 0.424 (-0.433, 1.281) | 0.332 |
| Model 4 | 0.290 (-0.064, 0.644) | 0.108 | reference | 0.134 (-0.405, 0.673) | 0.626 | 0.553 (-0.175, 1.280) | 0.136 |
| Standing long jump, cm |  |  |  |  |  |  |  |
| Model 1 | **2.613 (0.258, 4.967)** | **0.030** | reference | 1.431 (-2.118, 4.980) | 0.429 | **6.600 (1.809, 11.392)** | **0.007** |
| Model 2 | 2.265 (-0.074, 4.604) | 0.058 | reference | 0.886 (-2.658, 4.390) | 0.630 | **5.837 (1.079, 10.594)** | **0.016** |
| Model 3 | 2.025 (-0.272, 4.322) | 0.084 | reference | 0.432 (-3.026, 3.890) | 0.807 | **5.374 (0.708, 10.039)** | **0.024** |
| Model 4 | 2.107 (-0.138, 4.353) | 0.066 | reference | 0.449 (-2.929, 3.827) | 0.794 | 5.657 (1.098, 10.215) | 0.015 |
| Handgrip strength, kg |  |  |  |  |  |  |  |
| Left hand |  |  |  |  |  |  |  |
| Model 1 | -0.233 (-0.632, 0.166) | 0.252 | reference | -0.282 (-0.883, 0.319) | 0.358 | -0.579 (-1.390, 0.232) | 0.162 |
| Model 2 | -0.050 (-0.409, 0.310) | 0.786 | reference | 0.002 (-0.541, 0.545) | 0.994 | -0.195 (-0.928, 0.538) | 0.602 |
| Model 3 | -0.104 (-0.485, 0.277) | 0.592 | reference | -0.072 (-0.647, 0.504) | 0.807 | -0.321 (-1.097, 0.456) | 0.418 |
| Model 4 | -0.076 (-0.419, 0.267) | 0.664 | reference | -0.066 (-0.584, 0.452) | 0.804 | -0.225 (-0.923, 0.474) | 0.529 |
| Right hand |  |  |  |  |  |  |  |
| Model 1 | -0.277 (-0.683, 0.128) | 0.180 | reference | -0.400 (-1.012, 0.213) | 0.201 | \| -0.555 (-1.382, 0.272) \| \| --- \| | 0.188 |
| Model 2 | -0.078 (-0.438, 0.282) | 0.671 | reference | -0.090 (-0.634, 0.453) | 0.744 | -0.137 (-0.871, 0.597) | 0.715 |
| Model 3 | -0.136 (-0.520, 0.249) | 0.489 | reference | -0.169 (-0.751, 0.413) | 0.569 | -0.272 (-1.056, 0.513) | 0.498 |
| Model 4 | -0.105 (-0.446, 0.235) | 0.544 | reference | -0.163 (-0.678, 0.353) | 0.536 | -0.168 (-0.864, 0.527) | 0.636 |

Model 1 adjusted for gender, age, caregivers’ smoking, breakfast, weekly nocturnal sleep time, physical activity time and dietary intake (vegetable, fruit, coarse grain, meat, egg, fish and soybean).

Model 2 adjusted for gender, age, caregivers’ smoking, breakfast, weekly nocturnal sleep time, physical activity time, weight and dietary intake (vegetable, fruit, coarse grain, meat, egg, fish and soybean) (not applicable to weight).

Model 3 adjusted for gender, age, caregivers’ smoking, breakfast, weekly nocturnal sleep time, physical activity time, BMI and dietary intake (vegetable, fruit, coarse grain, meat, egg, fish and soybean) (not applicable to BMI).

Model 4 adjusted for gender, age, caregivers’ smoking, breakfast, weekly nocturnal sleep time, physical activity time, weight, BMI and dietary intake (vegetable, fruit, coarse grain, meat, egg, fish and soybean) (not applicable to weight and BMI).

**Table S3. Association between coarse grain intake and growth and development (*n* = 592)**

| **Growth and development** | **Coarse grain intake, times/week** | | | | | | | | |
| --- | --- | --- | --- | --- | --- | --- | --- | --- | --- |
|  | **Total (*n* = 592)** | | **< 1 (*n* = 113)** | **1** - **2 (*n* = 243)** | | **3** - **5 (*n* = 162)** | | **> 5 (*n* = 74)** | |
|  | ***β* (95% *CI*)** | ***P*-Value** |  | ***β* (95% *CI*)** | ***P*-Value** | ***β* (95% *CI*)** | ***P*-Value** | ***β* (95% *CI*)** | ***P*-Value** |
| Body height, cm |  |  |  |  |  |  |  |  |  |
| Model 1 | 0.592 (-0.002, 1.187) | 0.051 | reference | **1.563 (0.087, 3.038)** | **0.038** | **1.702 (0.088, 3.316)** | **0.039** | **2.001 (0.046, 3.956)** | **0.045** |
| Model 2 | 0.135 (-0.321, 0.591) | 0.562 | reference | 0.408 (-0.727, 1.544) | 0.481 | 0.457 (-0.785, 1.699) | 0.471 | 0.415 (-1.054, 1.956) | 0.557 |
| Model 3 | 0.378 (-0.184, 0.939) | 0.187 | reference | 1.021 (-0.376, 2.419) | 0.152 | 1.110 (-0.418, 2.639) | 0.155 | 1.292 (-0.559, 3.144) | 0.171 |
| Model 4 | 0.097 (-0.087, 0.280) | 0.301 | reference | 0.348 (-0.108, 0.803) | 0.134 | 0.434 (-0.064, 0.932) | 0.088 | 0.269 (-0.334, 0.872) | 0.382 |
| Weight, kg |  |  |  |  |  |  |  |  |  |
| Model 1 | **0.911 (0.147, 1.675)** | **0.019** | reference | **2.303 (0.410, 4.196)** | **0.017** | **2.483 (0.413, 4.554)** | **0.019** | **3.092 (0.585, 5.600)** | **0.016** |
| Model 3 | 0.152 (-0.135, 0.439) | 0.300 | reference | 0.364 (-0.350, 1.079) | 0.318 | 0.366 (-0.416, 1.147) | 0.359 | 0.553 (-0.393, 1.500) | 0.252 |
| BMI, kg/m^2^ |  |  |  |  |  |  |  |  |  |
| Model 1 | **0.324 (0.022, 0.626)** | **0.036** | reference | **0.828 (0.079, 1.576)** | **0.030** | **0.904 (0.085, 1.723)** | **0.031** | **1.084 (0.092, 2.076)** | **0.032** |
| Model 2 | -0.010 (-0.124, 0.103) | 0.859 | reference | -0.017 (-0.299, 0.266) | 0.909 | -0.006 (-0.316, 0.303) | 0.968 | -0.050 (-0.424, 0.325) | 0.795 |
| Sitting height, cm |  |  |  |  |  |  |  |  |  |
| Model 1 | 0.260 (-0.096, 0.615) | 0.152 | reference | 0.592 (-0.291, 1.476) | 0.189 | 0.667 (-0.299, 1.634) | 0.176 | 0.875 (-0.295, 2.046) | 0.143 |
| Model 2 | -0.007 (-0.284, 0.270) | 0.960 | reference | -0.083 (-0.773, 0.608) | 0.815 | -0.061 (-0.816, 0.695) | 0.875 | -0.031 (-0.946, 0.884) | 0.947 |
| Model 3 | 0.108 (-0.219, 0.434) | 0.519 | reference | 0.204 (-0.609, 1.018) | 0.623 | 0.243 (-0.647, 1.133) | 0.592 | 0.367 (-0.711, 1.445) | 0.505 |
| Model 4 | -0.023 (-0.238, 0.192) | 0.834 | reference | -0.108 (-0.644, 0.428) | 0.693 | -0.070 (-0.657, 0.516) | 0.814 | -0.108 (-0.818, 0.603) | 0.767 |
| Stature-sitting height index |  |  |  |  |  |  |  |  |  |
| Model 1 | -0.056 (-0.203, 0.091) | 0.454 | reference | -0.194 (-0.560, 0.172) | 0.300 | -0.196 (-0.597, 0.204) | 0.337 | -0.195 (-0.680, 0.290) | 0.431 |
| Model 2 | -0.070 (-0.217, 0.077) | 0.350 | reference | -0.231 (-0.598, 0.135) | 0.217 | -0.236 (-0.637, 0.164) | 0.248 | -0.245 (-0.731, 0.241) | 0.323 |
| Model 3 | -0.082 (-0.227, 0.063) | 0.269 | reference | -0.262 (-0.624, 0.100) | 0.156 | -0.271 (-0.667, 0.125) | 0.180 | -0.285 (-0.764, 0.195) | 0.245 |
| Model 4 | -0.067 (-0.209, 0.076) | 0.358 | reference | -0.226 (-0.581, 0.130) | 0.213 | -0.234 (-0.623, 0.154) | 0.237 | -0.229 (-0.700, 0.242) | 0.340 |
| Body fat rate, % |  |  |  |  |  |  |  |  |  |
| Model 1 | 0.550 (-0.172, 1.272) | 0.135 | reference | 0.928 (-0.857, 2.713) | 0.308 | 1.242 (-0.710, 3.194) | 0.212 | 1.713 (-0.652, 4.078) | 0.156 |
| Model 2 | -0.072 (-0.573, 0.429) | 0.778 | reference | -0.642 (-1.881, 0.598) | 0.310 | -0.451 (-1.806, 0.905) | 0.515 | -0.395 (-2.037, 1.248) | 0.638 |
| Model 3 | -0.073 (-0.502, 0.357) | 0.740 | reference | -0.662 (-1.723, 0.398) | 0.221 | -0.495 (-1.655, 0.655) | 0.403 | -0.369 (-1.775, 1.036) | 0.606 |
| Model 4 | -0.048 (-0.476, 0.379) | 0.825 | reference | -0.604 (-1.659, 0.451) | 0.262 | -0.436 (-1.590, 0.718) | 0.459 | -0.281 (-1.679, 1.118) | 0.694 |
| Fat weight, kg |  |  |  |  |  |  |  |  |  |
| Model 1 | **0.411 (0.015, 0.808)** | **0.042** | reference | 0.816 (-0.165, 1.798) | 0.103 | 0.970 (-0.104, 2.043) | 0.077 | **1.345 (0.044, 2.645)** | **0.043** |
| Model 2 | 0.014 (-0.202, 0.230) | 0.899 | reference | -0.187 (-0.722, 0.348) | 0.494 | -0.112 (-0.697, 0.473) | 0.708 | -0.002 (-0.711, 0.707) | 0.995 |
| Model 3 | 0.049 (-0.159, 0.258) | 0.643 | reference | -0.107 (-0.624, 0.410) | 0.684 | -0.039 (-0.605, 0.526) | 0.892 | 0.135 (-0.550, 0.820) | 0.699 |
| Model 4 | 0.021 (-0.181, 0.222) | 0.839 | reference | -0.176 (-0.676, 0.324) | 0.491 | -0.108 (-0.654, 0.439) | 0.699 | 0.031 (-0.631, 0.694) | 0.926 |
| Waist, cm |  |  |  |  |  |  |  |  |  |
| Model 1 | **0.884 (0.153, 1.616)** | **0.018** | reference | **1.978 (0.163, 3.794)** | **0.033** | **1.995 (0.010, 3.981)** | **0.049** | **3.159 (0.755, 5.564)** | **0.010** |
| Model 2 | 0.130 (-0.239, 0.500) | 0.489 | reference | 0.067 (-0.846, 0.980) | 0.886 | -0.066 (-1.065, 0.932) | 0.896 | 0.592 (-0.618, 1.803) | 0.337 |
| Model 3 | 0.209 (-0.163, 0.580) | 0.272 | reference | 0.246 (-0.673, 1.166) | 0.599 | 0.103 (-0.903, 1.109) | 0.841 | 0.891 (-0.328, 2.110) | 0.152 |
| Model 4 | 0.141 (-0.209, 0.491) | 0.429 | reference | 0.084 (-0.780, 0.948) | 0.849 | -0.060 (-1.004, 0.885) | 0.901 | 0.644 (-0.500, 1.789) | 0.270 |
| Chest, cm |  |  |  |  |  |  |  |  |  |
| Model 1 | **0.686 (0.056, 1.316)** | **0.033** | reference | **1.702 (0.142, 3.263)** | **0.033** | **1.868 (0.161, 3.575)** | **0.032** | **2.280 (0.213, 4.348)** | **0.031** |
| Model 2 | 0.008 (-0.265, 0.280) | 0.955 | reference | -0.011 (-0.687, 0.666) | 0.975 | 0.021 (-0.719, 0.761) | 0.956 | -0.020 (-0.916, 0.877) | 0.966 |
| Model 3 | 0.100 (-0.214, 0.415) | 0.532 | reference | 0.208 (-0.575, 0.990) | 0.603 | 0.236 (-0.620, 1.091) | 0.589 | 0.323 (-0.713, 1.359) | 0.541 |
| Model 4 | 0.013 (-0.255, 0.280) | 0.926 | reference | -0.003 (-0.668, 0.661) | 0.992 | 0.024 (-0.703, 0.750) | 0.949 | 0.003 (-0.878, 0.883) | 0.995 |
| Standing long jump, cm |  |  |  |  |  |  |  |  |  |
| Model 1 | 0.605 (-1.173, 2.383) | 0.505 | reference | 1.048 (-3.325, 5.422) | 0.639 | 4.734 (-0.050, 9.517) | 0.052 | -0.723 (-6.518, 5.072) | 0.807 |
| Model 2 | 0.903 (-0.866, 2.671) | 0.317 | reference | 1.829 (-2.517, 6.176) | 0.409 | **5.576 (0.822, 10.330)** | **0.022** | 0.326 (-5.434, 6.086) | 0.912 |
| Model 3 | 1.051 (-0.684, 2.786) | 0.235 | reference | 2.221 (-2.039, 6.481) | 0.307 | **6.015 (1.355, 10.674)** | **0.011** | 0.813 (-4.830, 6.456) | 0.778 |
| Model 4 | 0.857 (-0.840, 2.555) | 0.322 | reference | 1.757 (-2.408, 5.921) | 0.408 | 5.548 (0.993, 10.103) | 0.017 | 0.108 (-5.411, 5.627) | 0.969 |
| Handgrip strength, kg |  |  |  |  |  |  |  |  |  |
| Left hand |  |  |  |  |  |  |  |  |  |
| Model 1 | 0.189 (-0.112, 0.490) | 0.220 | reference | -0.125 (-0.865, 0.616) | 0.741 | 0.642 (-0.168, 1.452) | 0.121 | 0.156 (-0.826, 1.137) | 0.756 |
| Model 2 | 0.032 (-0.240, 0.304) | 0.819 | reference | -0.517 (-1.187, 0.152) | 0.130 | 0.218 (-0.514, 0.951) | 0.559 | -0.372 (-1.259, 0.516) | 0.412 |
| Model 3 | 0.091 (-0.197, 0.379) | 0.537 | reference | -0.372 (-1.080, 0.337) | 0.304 | 0.372 (-0.403, 1.147) | 0.347 | -0.168 (-1.107, 0.771) | 0.726 |
| Model 4 | 0.024 (-0.235, 0.283) | 0.855 | reference | -0.529 (-1.168, 0.109) | 0.104 | 0.214 (-0.485, 0.912) | 0.549 | -0.407 (-1.253, 0.439) | 0.346 |
| Right hand |  |  |  |  |  |  |  |  |  |
| Model 1 | **0.347 (0.041, 0.653)** | **0.026** | reference | 0.182 (-0.573, 0.936) | 0.637 | **1.141 (0.316, 1.967)** | **0.007** | 0.553 (-0.447, 1.553) | 0.279 |
| Model 2 | 0.176 (-0.096, 0.448) | 0.205 | reference | -0.246 (-0.917, 0.425) | 0.473 | 0.680 (-0.054, 1.414) | 0.069 | -0.022 (-0.911, 0.867) | 0.962 |
| Model 3 | 0.239 (-0.051, 0.530) | 0.107 | reference | -0.089 (-0.806, 0.628) | 0.808 | **0.845 (0.062, 1.629)** | **0.035** | 0.198 (-0.751, 1.147) | 0.683 |
| Model 4 | 0.168 (-0.090, 0.426) | 0.201 | reference | -0.258 (-0.894, 0.377) | 0.425 | 0.675 (-0.020, 1.370) | 0.057 | -0.059 (-0.901, 0.782) | 0.890 |

Model 1 adjusted for gender, age, caregivers’ smoking, breakfast, weekly nocturnal sleep time, physical activity time and dietary intake (vegetable, fruit, meat, milk, egg, fish and soybean).

Model 2 adjusted for gender, age, caregivers’ smoking, breakfast, weekly nocturnal sleep time, physical activity time, weight and dietary intake (vegetable, fruit, meat, milk, egg, fish and soybean) (not applicable to weight).

Model 3 adjusted for gender, age, caregivers’ smoking, breakfast, weekly nocturnal sleep time, physical activity time, BMI and dietary intake (vegetable, fruit, meat, milk, egg, fish and soybean) (not applicable to BMI).

Model 4 adjusted for gender, age, caregivers’ smoking, breakfast, weekly nocturnal sleep time, physical activity time, weight, BMI and dietary intake (vegetable, fruit, meat, milk, egg, fish and soybean) (not applicable to weight and BMI).

**Table S4. Association between** **soybean intake and growth and development (*n* = 592)**

| **Growth and development** | **Soybean intake, times/week** | | | | | | |
| --- | --- | --- | --- | --- | --- | --- | --- |
|  | **Total (*n* = 592)** | | **< 1 (*n* = 144)** | **1** - **2 (*n* = 245)** | | **> 2 (*n* = 203)** | |
|  | ***β* (95% *CI*)** | ***P*-Value** |  | ***β* (95% *CI*)** | ***P*-Value** | ***β* (95% *CI*)** | ***P*-Value** |
| Body height, cm |  |  |  |  |  |  |  |
| Model 1 | -0.579 (-1.331, 0.173) | 0.132 | reference | -0.868 (-2.234, 0.498) | 0.213 | -1.151 (-2.664, 0.363) | 0.136 |
| Model 2 | 0.049 (-0.528, 0.627) | 0.867 | reference | -0.123 (-1.172, 0.925) | 0.818 | 0.060 (-1.105, 1.226) | 0.919 |
| Model 3 | -0.240 (-0.952, 0.472) | 0.509 | reference | -0.444 (-1.736, 0.848) | 0.501 | -0.498 (-1.934, 0.937) | 0.496 |
| Model 4 | -0.142 (-0.375, 0.091) | 0.231 | reference | -0.505 (-0.926, -0.084) | 0.019 | -0.350 (-0.817, 0.117) | 0.142 |
| Weight, kg |  |  |  |  |  |  |  |
| Model 1 | **-1.250 (-2.217, -0.283)** | **0.011** | reference | -1.486 (-3.238, 0.267) | 0.097 | **-2.417 (-4.359, -0.475)** | **0.015** |
| Model 3 | -0.053 (-0.417, 0.311) | 0.775 | reference | 0.033 (-0.628, 0.694) | 0.922 | -0.080 (-0.814, 0.654) | 0.830 |
| BMI, kg/m^2^ |  |  |  |  |  |  |  |
| Model 1 | **-0.510 (-0.892, -0.128)** | **0.009** | reference | -0.648 (-1.341, 0.045) | 0.067 | **-0.997 (-1.766, -0.229)** | **0.011** |
| Model 2 | -0.052 (-0.196, 0.092) | 0.478 | reference | -0.104 (-0.365, 0.157) | 0.436 | -0.112 (-0.402, 0.179) | 0.451 |
| Sitting height, cm |  |  |  |  |  |  |  |
| Model 1 | -0.284 (-0.734, 0.165) | 0.215 | reference | -0.492 (-1.309, 0.326) | 0.239 | \| -0.569 (-1.475, 0.337) \| \| --- \| | 0.218 |
| Model 2 | 0.082 (-0.270, 0.433) | 0.649 | reference | -0.056 (-0.694, 0.582) | 0.863 | 0.139 (-0.569, 0.848) | 0.700 |
| Model 3 | -0.045 (-0.459, 0.370) | 0.833 | reference | -0.188 (-0.940, 0.565) | 0.625 | -0.101 (-0.937, 0.735) | 0.812 |
| Model 4 | 0.001 (-0.271, 0.273) | 0.994 | reference | -0.216 (-0.711, 0.279) | 0.393 | -0.032 (-0.583, 0.518) | 0.908 |
| Stature-sitting height index |  |  |  |  |  |  |  |
| Model 1 | 0.019 (-0.167, 0.205) | 0.841 | reference | -0.034 (-0.373, 0.305) | 0.844 | 0.036 (-0.340, 0.411) | 0.852 |
| Model 2 | 0.038 (-0.148, 0.224) | 0.687 | reference | -0.010 (-0.348, 0.329) | 0.954 | 0.075 (-0.301, 0.451) | 0.697 |
| Model 3 | 0.060 (-0.124, 0.244) | 0.525 | reference | 0.020 (-0.315, 0.355) | 0.909 | 0.118 (-0.254, 0.490) | 0.534 |
| Model 4 | 0.054 (-0.126, 0.235) | 0.555 | reference | 0.023 (-0.305, 0.351) | 0.891 | 0.110 (-0.255, 0.475) | 0.554 |
| Body fat rate, % |  |  |  |  |  |  |  |
| Model 1 | **-1.557 (-2.471, -0.643)** | **0.001** | reference | **-2.677 (-4.329, -1.024)** | **0.002** | \| **-3.157 (-4.988, -1.326)** \| \| --- \| | **0.001** |
| Model 2 | **-0.703 (-1.338, -0.068)** | **0.030** | reference | **-1.664 (-2.809, -0.519)** | **0.004** | **-1.510 (-2.782, -0.238)** | **0.020** |
| Model 3 | **-0.575 (-1.120, -0.030)** | **0.039** | reference | **-1.431 (-2.412, -0.450)** | **0.004** | **-1.241 (-2.330, -0.151)** | **0.026** |
| Model 4 | -0.583 (-1.125, -0.042) | 0.035 | reference | -1.426 (-2.401, -0.450) | 0.004 | -1.254 (-2.337, -0.170) | 0.023 |
| Fat weight, kg |  |  |  |  |  |  |  |
| Model 1 | **-0.842 (-1.344, -0.340)** | **0.001** | reference | **-1.273 (-2.182, -0.364)** | **0.006** | **-1.665 (-2.672, -0.658)** | **0.001** |
| Model 2 | **-0.297 (-0.571, -0.024)** | **0.033** | reference | **-0.626 (-1.120, -0.132)** | **0.013** | **-0.612 (-1.161, -0.063)** | **0.029** |
| Model 3 | **-0.272 (-0.536, -0.007)** | **0.044** | reference | **-0.500 (-1.028, -0.072)** | **0.024** | **-0.552 (-1.083, -0.021)** | **0.042** |
| Model 4 | -0.262 (-0.517, -0.006) | 0.045 | reference | -0.556 (-1.018, -0.094) | 0.018 | -0.537 (-1.050, -0.024) | 0.040 |
| Waist, cm |  |  |  |  |  |  |  |
| Model 1 | **-1.697 (-2.623, -0.771)** | **< 0.001** | reference | **-2.726 (-4.407, -1.046)** | **0.001** | \| **-3.417 (-5.279, -1.555)** \| \| --- \| | **< 0.001** |
| Model 2 | **-0.662 (-1.130, -0.194)** | **0.006** | reference | **-1.493 (-2.337, -0.650)** | **0.001** | **-1.411 (-2.348, -0.474)** | **0.003** |
| Model 3 | **-0.631 (-1.103, -0.159)** | **0.009** | reference | **-1.370 (-2.220, -0.519)** | **0.002** | **-1.330 (-2.275, -0.385)** | **0.006** |
| Model 4 | -0.607 (-1.050, -0.164) | 0.007 | reference | -1.385 (-2.183, -0.587) | 0.001 | -1.294 (-2.181, -0.407) | 0.004 |
| Chest, cm |  |  |  |  |  |  |  |
| Model 1 | -1.177 (-1.974, -0.379) | 0.730 | reference | 1.209 (-2.840, 5.258) | 0.559 | 0.265 (-4.222, 4.751) | 0.908 |
| Model 2 | -0.246 (-0.591, 0.099) | 0.162 | reference | **-0.716 (-1.341, -0.091)** | **0.025** | -0.516 (-1.210, 0.178) | 0.145 |
| Model 3 | -0.253 (-0.652, 0.146) | 0.214 | reference | -0.650 (-1.374, 0.073) | 0.078 | -0.512 (-1.316, 0.291) | 0.211 |
| Model 4 | -0.222 (-0.561, 0.117) | 0.199 | reference | -0.669 (-1.283, -0.056) | 0.033 | -0.466 (-1.148, 0.216) | 0.181 |
| Standing long jump, cm |  |  |  |  |  |  |  |
| Model 1 | 0.396 (-1.854, 2.646) | 0.730 | reference | 1.209 (-2.840, 5.258) | 0.559 | 0.265 (-4.222, 4.751) | 0.908 |
| Model 2 | -0.012 (-2.252, 2.228) | 0.992 | reference | 0.705 (-3.310, 4.719) | 0.731 | -0.555 (-5.016, 3.905) | 0.807 |
| Model 3 | -0.308 (-2.508, 1.893) | 0.784 | reference | 0.290 (-3.649, 4.229) | 0.885 | -1.149 (-5.525, 3.228) | 0.607 |
| Model 4 | -0.240 (-2.391, 1.911) | 0.827 | reference | 0.248 (-3.600, 4.096) | 0.900 | -1.046 (-5.322, 3.229) | 0.631 |
| Handgrip strength, kg |  |  |  |  |  |  |  |
| Left hand |  |  |  |  |  |  |  |
| Model 1 | -0.041 (-0.422, 0.340) | 0.832 | reference | -0.208 (-0.893, 0.478) | 0.553 | -0.036 (-0.796, 0.724) | 0.926 |
| Model 2 | 0.174 (-0.171, 0.519) | 0.322 | reference | 0.046 (-0.573, 0.664) | 0.885 | 0.376 (-0.311, 1.064) | 0.283 |
| Model 3 | 0.113 (-0.252, 0.478) | 0.545 | reference | -0.014 (-0.670, 0.641) | 0.966 | 0.262 (-0.466, 0.990) | 0.481 |
| Model 4 | 0.136 (-0.192, 0.465) | 0.416 | reference | -0.028 (-0.618, 0.562) | 0.925 | 0.297 (-0.359, 0.952) | 0.375 |
| Right hand |  |  |  |  |  |  |  |
| Model 1 | -0.247 (-0.634, 0.141) | 0.212 | reference | -0.429 (-1.128, 0.269) | 0.228 | -0.488 (-1.262, 0.286) | 0.217 |
| Model 2 | -0.012 (-0.357, 0.332) | 0.944 | reference | -0.154 (-0.773, 0.466) | 0.627 | -0.039 (-0.727, 0.649) | 0.911 |
| Model 3 | -0.077 (-0.445, 0.291) | 0.682 | reference | -0.217 (-0.880, 0.445) | 0.520 | -0.161 (-0.898, 0.575) | 0.667 |
| Model 4 | -0.052 (-0.379, 0.274) | 0.754 | reference | -0.233 (-0.820, 0.354) | 0.437 | -0.124 (-0.776, 0.528) | 0.709 |

Model 1 adjusted for gender, age, caregivers’ smoking, breakfast, weekly nocturnal sleep time, physical activity time and dietary intake (vegetable, fruit, meat, milk, egg, fish and coarse grain).

Model 2 adjusted for gender, age, caregivers’ smoking, breakfast, weekly nocturnal sleep time, physical activity time, weight and dietary intake (vegetable, fruit, meat, milk, egg, fish and coarse grain) (not applicable to weight).

Model 3 adjusted for gender, age, caregivers’ smoking, breakfast, weekly nocturnal sleep time, physical activity time, BMI and dietary intake (vegetable, fruit, meat, milk, egg, fish and coarse grain) (not applicable to BMI).

Model 4 adjusted for gender, age, caregivers’ smoking, breakfast, weekly nocturnal sleep time, physical activity time, weight, BMI and dietary intake (vegetable, fruit, meat, milk, egg, fish and coarse grain) (not applicable to weight and BMI).

**Table S5. Association between vegetable intake and growth and development (*n* = 592)**

| **Growth and development** | **Vegetable intake, times/week** | | | | | | |
| --- | --- | --- | --- | --- | --- | --- | --- |
|  | **Total (*n* = 592)** | | **< 3 (*n* = 129)** | **3** - **5 (*n* = 169)** | | **> 5 (*n* = 294)** | |
|  | ***β* (95% *CI*)** | ***P*-Value** |  | ***β* (95% *CI*)** | ***P*-Value** | ***β* (95% *CI*)** | ***P*-Value** |
| Body height, cm |  |  |  |  |  |  |  |
| Model 1 | 0.060 (-0.609, 0.728) | 0.861 | reference | -0.290 (-1.755, 1.176) | 0.698 | -0.062 (-1.428, 1.303) | 0.929 |
| Model 2 | -0.115 (-0.626, 0.396) | 0.660 | reference | -0.264 (-1.387, 0.858) | 0.644 | -0.337 (-1.383, 0.709) | 0.528 |
| Model 3 | 0.007 (-0.623, 0.637) | 0.983 | reference | -0.295 (-1.677, 1.087) | 0.676 | -0.155 (-1.433, 1.134) | 0.814 |
| Model 4 | -0.290 (-0.496, -0.084) | 0.006 | reference | -0.167 (-0.617, 0.283) | 0.468 | -0.557 (-0.976, -0.137) | 0.009 |
| Weight, kg |  |  |  |  |  |  |  |
| Model 1 | 0.347 (-0.513, 1.207) | 0.429 | reference | -0.051 (-1.930, 1.829) | 0.958 | 0.547 (-1.204, 2.299) | 0.540 |
| Model 3 | 0.161 (-0.161, 0.483) | 0.328 | reference | -0.069 (-0.776, 0.637) | 0.847 | 0.217 (-0.441, 0.876) | 0.518 |
| BMI, kg/m^2^ |  |  |  |  |  |  |  |
| Model 1 | 0.080 (-0.260, 0.419) | 0.647 | reference | 0.008 (-0.735, 0.751) | 0.983 | 0.141 (-0.552, 0.834) | 0.690 |
| Model 2 | -0.048 (-0.175, 0.080) | 0.463 | reference | 0.027 (-0.253, 0.306) | 0.852 | -0.060 (-0.320, 0.201) | 0.653 |
| Sitting height, cm |  |  |  |  |  |  |  |
| Model 1 | 0.303 (-0.097, 0.703) | 0.138 | reference | 0.133 (-0.744, 1.010) | 0.766 | 0.512 (-0.305, 1.329) | 0.220 |
| Model 2 | 0.201 (-0.110, 0.512) | 0.205 | reference | 0.148 (-0.535, 0.830) | 0.671 | 0.351 (-0.285, 0.987) | 0.279 |
| Model 3 | 0.265 (-0.101, 0.632) | 0.156 | reference | 0.129 (-0.676, 0.934) | 0.753 | 0.446 (-0.304, 1.196) | 0.244 |
| Model 4 | 0.127 (-0.114, 0.368) | 0.300 | reference | 0.189 (-0.341, 0.718) | 0.485 | 0.259 (-0.235, 0.753) | 0.303 |
| Stature-sitting height index |  |  |  |  |  |  |  |
| Model 1 | **0.206 (0.041, 0.371)** | **0.015** | reference | 0.226 (-0.138, 0.589) | 0.223 | **0.414 (0.075, 0.753)** | **0.017** |
| Model 2 | **0.200 (0.036, 0.365)** | **0.017** | reference | 0.227 (-0.135, 0.598) | 0.220 | **0.405 (0.068, 0.743)** | **0.019** |
| Model 3 | **0.199 (0.036, 0.362)** | **0.016** | reference | 0.225 (-0.133, 0.584) | 0.218 | **0.402 (0.069, 0.736)** | **0.018** |
| Model 4 | 0.215 (0.055, 0.375) | 0.008 | reference | 0.218 (-0.133, 0.570) | 0.223 | 0.424 (0.097, 0.752) | 0.011 |
| Body fat rate, % |  |  |  |  |  |  |  |
| Model 1 | 0.264 (-0.549, 1.076) | 0.525 | reference | -0.282 (-2.055, 1.490) | 0.755 | 0.459 (-1.193, 2.110) | 0.586 |
| Model 2 | 0.026 (-0.535, 0.588) | 0.926 | reference | -0.248 (-1.473, 0.977) | 0.692 | 0.085 (-1.056, 1.227) | 0.883 |
| Model 3 | 0.111 (-0.371, 0.592) | 0.653 | reference | -0.298 (-1.347, 0.751) | 0.578 | 0.188 (-0.790, 1.165) | 0.706 |
| Model 4 | 0.136 (-0.343, 0.616) | 0.577 | reference | -0.309 (-1.352, 0.734) | 0.562 | 0.223 (-0.749, 1.195) | 0.653 |
| Fat weight, kg |  |  |  |  |  |  |  |
| Model 1 | 0.283 (-0.163, 0.730) | 0.213 | reference | 0.149 (-0.825, 1.124) | 0.764 | 0.530 (-0.379, 1.438) | 0.253 |
| Model 2 | 0.132 (-0.110, 0.374) | 0.285 | reference | 0.171 (-0.358, 0.700) | 0.526 | 0.291 (-0.202, 0.784) | 0.247 |
| Model 3 | 0.194 (-0.039, 0.428) | 0.103 | reference | 0.140 (-0.371, 0.652) | 0.591 | 0.372 (-0.104, 0.849) | 0.126 |
| Model 4 | 0.164 (-0.062, 0.390) | 0.154 | reference | 0.153 (-0.341, 0.647) | 0.543 | 0.332 (-0.129, 0.792) | 0.158 |
| Waist, cm |  |  |  |  |  |  |  |
| Model 1 | 0.721 (-0.102, 1.544) | 0.086 | reference | 0.548 (-1.254, 2.351) | 0.551 | 1.395 (-0.285, 3.074) | 0.104 |
| Model 2 | **0.434 (0.020, 0.848)** | **0.040** | reference | 0.590 (-0.312, 1.493) | 0.200 | **0.940 (0.099, 1.782)** | **0.028** |
| Model 3 | **0.555 (0.138, 0.972)** | **0.009** | reference | 0.531 (-0.378, 1.441) | 0.252 | **1.100 (0.252, 1.948)** | **0.011** |
| Model 4 | 0.484 (0.092, 0.876) | 0.016 | reference | 0.562 (-0.291, 1.416) | 0.197 | 1.003 (0.207, 1.799) | 0.013 |
| Chest, cm |  |  |  |  |  |  |  |
| Model 1 | 0.272 (-0.437, 0.982) | 0.452 | reference | 0.505 (-1.044, 2.055) | 0.523 | 0.546 (-0.897, 1.990) | 0.458 |
| Model 2 | 0.014 (-0.292, 0.319) | 0.929 | reference | 0.543 (-0.126, 1.212) | 0.111 | 0.139 (-0.484, 0.762) | 0.661 |
| Model 3 | 0.128 (-0.224, 0.481) | 0.475 | reference | 0.491 (-0.283, 1.265) | 0.214 | 0.292 (-0.429, 1.013) | 0.427 |
| Model 4 | 0.036 (-0.264, 0.336) | 0.815 | reference | 0.531 (-0.126, 1.188) | 0.113 | 0.166 (-0.446, 0.778) | 0.595 |
| Standing long jump, cm |  |  |  |  |  |  |  |
| Model 1 | 0.548 (-1.453, 2.549) | 0.591 | reference | **-5.817 (-10.160, -1.474)** | **0.009** | 0.050 (-3.997, 4.096) | 0.981 |
| Model 2 | 0.661 (-1.321, 2.644) | 0.513 | reference | **-5.834 (-10.130, -1.538)** | **0.008** | 0.236 (-3.768, 4.239) | 0.908 |
| Model 3 | 0.658 (-1.288, 2.604) | 0.508 | reference | **-5.806 (-10.019, -1.593)** | **0.007** | 0.249 (-3.677, 4.176) | 0.901 |
| Model 4 | 0.453 (-1.451, 2.356) | 0.641 | reference | -5.717 (-9.834, -1.601) | 0.006 | -0.028 (-3.864, 3.809) | 0.989 |
| Handgrip strength, kg |  |  |  |  |  |  |  |
| Left hand |  |  |  |  |  |  |  |
| Model 1 | -0.124 (-0.462, 0.215) | 0.475 | reference | -0.438 (-1.173, 0.297) | 0.243 | -0.411 (-1.096, 0.274) | 0.240 |
| Model 2 | -0.183 (-0.488, 0.122) | 0.238 | reference | -0.429 (-1.091, 0.232) | 0.204 | -0.504 (-1.121, 0.113) | 0.109 |
| Model 3 | -0.148 (-0.471, 0.175) | 0.370 | reference | -0.440 (-1.142, 0.261) | 0.218 | -0.453 (-1.106, 0.200) | 0.174 |
| Model 4 | -0.218 (-0.509, 0.073) | 0.142 | reference | -0.410 (-1.041, 0.221) | 0.202 | -0.547 (-1.135, 0.041) | 0.068 |
| Right hand |  |  |  |  |  |  |  |
| Model 1 | 0.030 (-0.314, 0.375) | 0.863 | reference | -0.446 (-1.195, 0.304) | 0.244 | -0.107 (-0.805, 0.592) | 0.765 |
| Model 2 | -0.035 (-0.340, 0.270) | 0.823 | reference | -0.436 (-1.099, 0.227) | 0.197 | -0.208 (-0.826, 0.410) | 0.509 |
| Model 3 | 0.004 (-0.322, 0.330) | 0.981 | reference | -0.448 (-1.157, 0.260) | 0.215 | -0.153 (-0.813, 0.508) | 0.651 |
| Model 4 | -0.071 (-0.360, 0.218) | 0.629 | reference | -0.416 (-1.044, 0.212) | 0.194 | -0.254 (-0.839, 0.332) | 0.395 |

Model 1 adjusted for gender, age, caregivers’ smoking, breakfast, weekly nocturnal sleep time, physical activity time and dietary intake (vegetable, fruit, meat, milk, egg, fish and coarse grain).

Model 2 adjusted for gender, age, caregivers’ smoking, breakfast, weekly nocturnal sleep time, physical activity time, weight and dietary intake (fruit, meat, coarse grain, milk, egg, fish and soybean) (not applicable to weight).

Model 3 adjusted for gender, age, caregivers’ smoking, breakfast, weekly nocturnal sleep time, physical activity time, BMI and dietary intake (fruit, meat, coarse grain, milk, egg, fish and soybean) (not applicable to BMI).

Model 4 adjusted for gender, age, caregivers’ smoking, breakfast, weekly nocturnal sleep time, physical activity time, weight, BMI and dietary intake (fruit, meat, coarse grain, milk, egg, fish and soybean) (not applicable to weight and BMI).

**Table S6. Association between fruit intake and growth and development (*n* = 592)**

| **Growth and development** | **Fruit intake, times/week** | | | | | | |
| --- | --- | --- | --- | --- | --- | --- | --- |
|  | **Total (*n* = 592)** | | **< 3 (*n* = 182)** | **3** - **5 (*n* = 240)** | | **> 5 (*n* = 170)** | |
|  | ***β* (95% *CI*)** | ***P*-Value** |  | ***β* (95% *CI*)** | ***P*-Value** | ***β* (95% *CI*)** | ***P*-Value** |
| Body height, cm |  |  |  |  |  |  |  |
| Model 1 | 0.065 (-0.644, 0.773) | 0.858 | reference | 0.070 (-1.190, 1.329) | 0.914 | 0.064 (-1.353, 1.481) | 0.930 |
| Model 2 | 0.051 (-0.489, 0.592) | 0.852 | reference | 0.294 (-0.671, 1.260) | 0.550 | 0.081 (-1.004, 1.167) | 0.883 |
| Model 3 | 0.052 (-0.615, 0.719) | 0.879 | reference | 0.247 (-0.942, 1.436) | 0.684 | 0.057 (-1.279, 1.394) | 0.933 |
| Model 4 | 0.087 (-0.131, 0.304) | 0.436 | reference | -0.097 (-0.484, 0.290) | 0.622 | 0.164 (-0.271, 0.599) | 0.461 |
| Weight, kg |  |  |  |  |  |  |  |
| Model 1 | 0.026 (-0.884, 0.937) | 0.955 | reference | -0.449 (-2.065, 1.167) | 0.586 | -0.035 (-1.852, 1.783) | 0.970 |
| Model 3 | -0.019 (-0.360, 0.322) | 0.914 | reference | 0.186 (-0.422, 0.794) | 0.549 | -0.057 (-0.741, 0.626) | 0.869 |
| BMI, kg/m^2^ |  |  |  |  |  |  |  |
| Model 1 | 0.019 (-0.341, 0.379) | 0.917 | reference | -0.271 (-0.910, 0.368) | 0.406 | 0.010 (-0.709, 0.729) | 0.979 |
| Model 2 | 0.010 (-0.125, 0.144) | 0.889 | reference | -0.107 (-0.347, 0.134) | 0.385 | 0.022 (-0.248, 0.293) | 0.871 |
| Sitting height, cm |  |  |  |  |  |  |  |
| Model 1 | 0.004 (-0.420, 0.427) | 0.987 | reference | 0.167 (-0.587, 0.922) | 0.663 | -0.057 (-0.905, 0.791) | 0.895 |
| Model 2 | -0.004 (-0.333, 0.325) | 0.980 | reference | 0.299 (-0.288, 0.886) | 0.318 | -0.047 (-0.707, 0.613) | 0.889 |
| Model 3 | -0.005 (-0.394, 0.383) | 0.978 | reference | 0.295 (-0.398, 0.987) | 0.404 | -0.062 (-0.840, 0.717) | 0.877 |
| Model 4 | 0.011 (-0.244, 0.265) | 0.935 | reference | 0.135 (-0.321, 0.591) | 0.561 | -0.012 (-0.524, 0.500) | 0.963 |
| Stature-sitting height index |  |  |  |  |  |  |  |
| Model 1 | -0.012 (-0.187, 0.163) | 0.893 | reference | 0.095 (-0.218, 0.407) | 0.553 | -0.407 (-0.399, 0.304) | 0.792 |
| Model 2 | -0.012 (-0.187, 0.162) | 0.889 | reference | 0.102 (-0.210, 0.413) | 0.522 | -0.047 (-0.397, 0.304) | 0.794 |
| Model 3 | -0.014 (-0.186, 0.159) | 0.878 | reference | 0.117 (-0.191, 0.425) | 0.457 | -0.048 (-0.394, 0.298) | 0.786 |
| Model 4 | -0.015 (-0.184, 0.154) | 0.859 | reference | 0.136 (-0.167, 0.438) | 0.379 | -0.054 (-0.393, 0.286) | 0.756 |
| Body fat rate, % |  |  |  |  |  |  |  |
| Model 1 | -0.299 (-1.160, 0.562) | 0.496 | reference | -0.537 (-2.061, 0.986) | 0.489 | -0.821 (-2.535, 0.893) | 0.348 |
| Model 2 | -0.317 (-0.912, 0.278) | 0.296 | reference | -0.231 (-1.285, 0.822) | 0.667 | -0.797 (-1.982, 0.387) | 0.187 |
| Model 3 | -0.336 (-0.846, 0.174) | 0.197 | reference | -0.017 (-0.919, 0.886) | 0.971 | -0.840 (-1.854, 0.175) | 0.105 |
| Model 4 | -0.339 (-0.846, 0.168) | 0.190 | reference | 0.013 (-0.884, 0.911) | 0.977 | -0.849 (-1.857, 0.160) | 0.099 |
| Fat weight, kg |  |  |  |  |  |  |  |
| Model 1 | -0.187 (-0.660, 0.286) | 0.438 | reference | -0.439 (-1.277, 0.399) | 0.305 | -0.455 (-1.398, 0.487) | 0.344 |
| Model 2 | -0.198 (-0.455, 0.058) | 0.129 | reference | -0.243 (-0.698, 0.211) | 0.294 | -0.440 (-0.952, 0.071) | 0.092 |
| Model 3 | -0.208 (-0.456, 0.039) | 0.099 | reference | -0.136 (-0.576, 0.303) | 0.543 | -0.466 (-0.961, 0.028) | 0.065 |
| Model 4 | -0.205 (-0.444, 0.034) | 0.093 | reference | -0.171 (-0.596, 0.254) | 0.429 | -0.455 (-0.933, 0.022) | 0.062 |
| Waist, cm |  |  |  |  |  |  |  |
| Model 1 | -0.141 (-1.013, 0.731) | 0.751 | reference | -0.219 (-1.769, 1.330) | 0.782 | -0.440 (-2.183, 1.303) | 0.621 |
| Model 2 | -0.163 (-0.601, 0.275) | 0.466 | reference | 0.153 (-0.623, 0.930) | 0.698 | -0.411 (-1.284, 0.462) | 0.356 |
| Model 3 | -0.181 (-0.623, 0.260) | 0.421 | reference | 0.348 (-0.434, 1.131) | 0.383 | -0.460 (-1.340, 0.419) | 0.305 |
| Model 4 | -0.173 (-0.588, 0.242) | 0.414 | reference | 0.265 (-0.469, 1.000) | 0.479 | -0.435 (-1.260, 0.391) | 0.302 |
| Chest, cm |  |  |  |  |  |  |  |
| Model 1 | 0.135 (-0.617, 0.886) | 0.726 | reference | -0.278 (-1.610, 1.054) | 0.683 | 0.155 (-1.343, 1.654) | 0.839 |
| Model 2 | 0.115 (-0.208, 0.438) | 0.486 | reference | 0.058 (-0.519, 0.631) | 0.849 | 0.181 (-0.466, 0.828) | 0.583 |
| Model 3 | 0.100 (-0.274, 0.473) | 0.601 | reference | 0.211 (-0.454, 0.877) | 0.534 | 0.138 (-0.611, 0.886) | 0.718 |
| Model 4 | 0.110 (-0.207, 0.428) | 0.495 | reference | 0.104 (-0.461, 0.669) | 0.719 | 0.171 (-0.464, 0.806) | 0.598 |
| Standing long jump, cm |  |  |  |  |  |  |  |
| Model 1 | -0.570 (-2.689, 1.549) | 0.598 | reference | 1.420 (-2.314, 5.153) | 0.456 | -1.267 (-5.467, 2.933) | 0.554 |
| Model 2 | -0.561 (-2.660, 1.537) | 0.600 | reference | 1.267 (-2.427, 4.961) | 0.501 | -1.279 (-5.433, 2.876) | 0.546 |
| Model 3 | -0.543 (-2.604, 1.517) | 0.605 | reference | 1.035 (-2.589, 4.660) | 0.575 | -1.253 (-5.327, 2.821) | 0.547 |
| Model 4 | -0.520 (-2.533, 1.494) | 0.613 | reference | 0.798 (-2.743, 4.340) | 0.659 | -1.180 (-5.160, 2.800) | 0.561 |
| Handgrip strength, kg |  |  |  |  |  |  |  |
| Left hand |  |  |  |  |  |  |  |
| Model 1 | -0.086 (-0.444, 0.273) | 0.640 | reference | 0.223 (-0.409, 0.855) | 0.489 | -0.303 (-1.014, 0.408) | 0.404 |
| Model 2 | -0.090 (-0.413, 0.233) | 0.584 | reference | 0.300 (-0.270, 0.869) | 0.302 | -0.297 (-0.937, 0.343) | 0.363 |
| Model 3 | -0.091 (-0.433, 0.251) | 0.601 | reference | 0.304 (-0.299, 0.907) | 0.323 | -0.306 (-0.984, 0.372) | 0.377 |
| Model 4 | -0.083 (-0.391, 0.224) | 0.596 | reference | 0.223 (-0.319, 0.766) | 0.420 | -0.281 (-0.891, 0.329) | 0.367 |
| Right hand |  |  |  |  |  |  |  |
| Model 1 | -0.213 (-0.578, 0.152) | 0.253 | reference | -0.031 (-0.675, 0.613) | 0.924 | -0.527 (-1.252, 0.198) | 0.154 |
| Model 2 | -0.218 (-0.540, 0.105) | 0.186 | reference | 0.052 (-0.518, 0.622) | 0.858 | -0.521 (-1.162, 0.121) | 0.112 |
| Model 3 | -0.219 (-0.564, 0.126) | 0.213 | reference | 0.057 (-0.552, 0.667) | 0.854 | -0.530 (-1.216, 0.155) | 0.129 |
| Model 4 | -0.210 (-0.516, 0.095) | 0.178 | reference | -0.029 (-0.569, 0.511) | 0.916 | -0.503 (-1.111, 0.104) | 0.104 |

Model 1 adjusted for gender, age, caregivers’ smoking, breakfast, weekly nocturnal sleep time, physical activity time and dietary intake (vegetable, meat, coarse grain, milk, egg, fish and soybean).

Model 2 adjusted for gender, age, caregivers’ smoking, breakfast, weekly nocturnal sleep time, physical activity time, weight and dietary intake (vegetable, meat, coarse grain, milk, egg, fish and soybean) (not applicable to weight).

Model 3 adjusted for gender, age, caregivers’ smoking, breakfast, weekly nocturnal sleep time, physical activity time, BMI and dietary intake (vegetable, meat, coarse grain, milk, egg, fish and soybean) (not applicable to BMI).

Model 4 adjusted for gender, age, caregivers’ smoking, breakfast, weekly nocturnal sleep time, physical activity time, weight, BMI and dietary intake (vegetable, meat, coarse grain, milk, egg, fish and soybean) (not applicable to weight and BMI).

**Table S7. Association between egg intake and growth and development (*n* = 592)**

| **Growth and development** | **Egg intake, times/week** | | | | | | | | |
| --- | --- | --- | --- | --- | --- | --- | --- | --- | --- |
|  | **Total (*n* = 592)** | | **< 1 (*n* = 91)** | **1** - **2 (*n* = 234)** | | **3** - **5 (*n* = 182)** | | **> 5 (*n* = 85)** | |
|  | ***β* (95% *CI*)** | ***P*-Value** |  | ***β* (95% *CI*)** | ***P*-Value** | ***β* (95% *CI*)** | ***P*-Value** | ***β* (95% *CI*)** | ***P*-Value** |
| Body height, cm |  |  |  |  |  |  |  |  |  |
| Model 1 | 0.282 (-0.331, 0.895) | 0.367 | reference | -1.210 (-2.764, 0.344) | 0.127 | -0.261 (-1.925, 1.403) | 0.759 | 0.265 (-1.772, 2.301) | 0.799 |
| Model 2 | 0.130 (-0.338, 0.599) | 0.585 | reference | -0.273 (-1.467, 0.920) | 0.654 | -0.222 (-1.496, 1.053) | 0.733 | 0.498 (-1.062, 2.058) | 0.532 |
| Model 3 | 0.234 (-0.344, 0.811) | 0.428 | reference | -0.764 (-2.233, 0.706) | 0.308 | -0.242 (-1.811, 1.327) | 0.763 | 0.477 (-1.444, 2.399) | 0.626 |
| Model 4 | -0.006 (-0.195, 0.182) | 0.947 | reference | -0.265 (-0.744, 0.213) | 0.278 | -0.223 (-0.734, 0.288) | 0.392 | -0.070 (-0.696, 0.556) | 0.826 |
| Weight, kg |  |  |  |  |  |  |  |  |  |
| Model 1 | 0.302 (-0.486, 1.090) | 0.452 | reference | -1.869 (-3.863, 0.124) | 0.066 | -0.078 (-2.212, 2.056) | 0.943 | -0.465 (-3.077, 2.147) | 0.727 |
| Model 3 | 0.130 (-0.165, 0.425) | 0.389 | reference | -0.270 (-1.021, 0.482) | 0.482 | -0.010 (-0.813, 0.792) | 0.980 | 0.296 (-0.686, 1.278) | 0.555 |
| BMI, kg/m^2^ |  |  |  |  |  |  |  |  |  |
| Model 1 | 0.074 (-0.238, 0.385) | 0.644 | reference | -0.683 (-1.471, 0.106) | 0.090 | -0.029 (-0.873, 0.815) | 0.946 | -0.325 (-1.358, 0.708) | 0.538 |
| Model 2 | -0.037 (-0.154, 0.079) | 0.531 | reference | 0.002 (-0.295, 0.300) | 0.988 | 0.000 (-0.318, 0.317) | 0.998 | -0.154 (-0.543, 0.234) | 0.436 |
| Sitting height, cm |  |  |  |  |  |  |  |  |  |
| Model 1 | 0.160 (-0.207, 0.526) | 0.393 | reference | -0.580 (-1.511, 0.350) | 0.221 | -0.115 (-1.111, 0.881) | 0.821 | 0.240 (-0.979, 1.459) | 0.700 |
| Model 2 | 0.071 (-0.214, 0.356) | 0.625 | reference | -0.032 (-0.758, 0.694) | 0.930 | -0.092 (-0.867, 0.683) | 0.816 | 0.376 (-0.572, 1.325) | 0.437 |
| Model 3 | 0.125 (-0.211, 0.461) | 0.466 | reference | -0.260 (-1.116, 0.596) | 0.551 | -0.101 (-1.015, 0.813) | 0.828 | 0.392 (-0.727, 1.511) | 0.492 |
| Model 4 | 0.014 (-0.207, 0.234) | 0.904 | reference | -0.029 (-0.592, 0.534) | 0.920 | -0.092 (-0.694, 0.509) | 0.763 | 0.139 (-0.598, 0.875) | 0.712 |
| Stature-sitting height index |  |  |  |  |  |  |  |  |  |
| Model 1 | 0.003 (-0.148, 0.154) | 0.970 | reference | 0.071 (-0.315, 0.456) | 0.719 | 0.030 (-0.382, 0.443) | 0.885 | \| 0.066 (-0.439, 0.571) \| \| --- \| | 0.797 |
| Model 2 | -0.002 (-0.153, 0.149) | 0.982 | reference | 0.101(-0.284, 0.486) | 0.608 | 0.032 (-0.380, 0.443) | 0.880 | 0.074 (-0.430, 0.577) | 0.774 |
| Model 3 | -0.003 (-0.152, 0.146) | 0.969 | reference | 0.127 (-0.254, 0.508) | 0.513 | 0.033 (-0.374, 0.440) | 0.874 | 0.093 (-0.405, 0.591) | 0.714 |
| Model 4 | 0.010 (-0.137, 0.156) | 0.896 | reference | 0.100 (-0.273, 0.474) | 0.599 | 0.032 (-0.367, 0.431) | 0.876 | 0.123 (-0.366, 0.611) | 0.622 |
| Body fat rate, % |  |  |  |  |  |  |  |  |  |
| Model 1 | 0.226 (-0.519, 0.970) | 0.552 | reference | -0.893 (-2.773, 0.987) | 0.352 | 0.864 (-1.148, 2.876) | 0.400 | -0.680 (-3.144, 1.783) | 0.588 |
| Model 2 | 0.019 (-0.496, 0.534) | 0.942 | reference | 0.382 (-0.921, 1684) | 0.566 | 0.917 (-0.473, 2.308) | 0.196 | -0.363 (-2.065, 1.339) | 0.676 |
| Model 3 | 0.084 (-0.357, 0.526) | 0.709 | reference | 0.420 (-0.696, 1.535) | 0.461 | 0.920 (-0.271, 2.111) | 0.130 | -0.056 (-1.514, 1.403) | 0.940 |
| Model 4 | 0.105 (-0.334, 0.544) | 0.639 | reference | 0.376 (-0.733, 1.486) | 0.506 | 0.918 (-0.266, 2.102) | 0.129 | -0.008 (-1.458, 1.442) | 0.991 |
| Fat weight, kg |  |  |  |  |  |  |  |  |  |
| Model 1 | 0.214 (-0.195, 0.623) | 0.306 | reference | -0.579 (-1.613, 0.455) | 0.272 | 0.582 (-0.525, 1.689) | 0.303 | -0.204 (-1.558, 1.151) | 0.768 |
| Model 2 | 0.082 (-0.140, 0.304) | 0.470 | reference | 0.235 (-0.327, 0.798) | 0.412 | **0.616 (0.016, 1.217)** | **0.044** | -0.001 (-0.736, 0.734) | 0.998 |
| Model 3 | 0.131 (-0.083, 0.345) | 0.229 | reference | 0.183 (-0.360, 0.727) | 0.509 | **0.614 (0.034, 1.195)** | **0.038** | 0.159 (-0.552, 0.870) | 0.661 |
| Model 4 | 0.107 (-0.100, 0.314) | 0.310 | reference | 0.234 (-0.291, 0.759) | 0.383 | 0.616 (0.056, 1.177) | 0.031 | 0.104 (-0.583, 0.791) | 0.767 |
| Waist, cm |  |  |  |  |  |  |  |  |  |
| Model 1 | 0.353 (-0.402, 1.107) | 0.360 | reference | -0.511 (-2.423, 1.400) | 0.600 | 0.961 (-1.085, 3.008) | 0.357 | 0.014 (-2.491, 2.519) | 0.991 |
| Model 2 | 0.102 (-0.277, 0.482) | 0.597 | reference | **1.040 (0.080, 2.000)** | **0.034** | 1.026 (0.002, 2.051) | 0.050 | 0.400 (-0.854, 1.655) | 0.532 |
| Model 3 | 0.199 (-0.183, 0.581) | 0.307 | reference | 0.918 (-0.050, 1.885) | 0.063 | 1.022 (-0.011, 2.055) | 0.052 | 0.694 (-0.571, 1.958) | 0.282 |
| Model 4 | 0.141 (-0.218, 0.501) | 0.441 | reference | 1.038 (0.130, 1.946) | 0.025 | 1.027 (0.058, 1.996) | 0.038 | 0.562 (-0.625, 1.749) | 0.353 |
| Chest, cm |  |  |  |  |  |  |  |  |  |
| Model 1 | 0.480 (-0.170, 1.130) | 0.148 | reference | -1.037 (-2.681, 0.606) | 0.216 | 0.798 (-0.962, 2.557) | 0.374 | 0.230 (-1.924, 2.384) | 0.834 |
| Model 2 | 0.255 (-0.025, 0.535) | 0.074 | reference | 0.353 (-0.358, 1.064) | 0.330 | **0.856 (0.097, 1.615)** | **0.027** | 0.576 (-0.353, 1.505) | 0.224 |
| Model 3 | **0.347 (0.024, 0.670)** | **0.035** | reference | 0.196 (-0.627, 1.019) | 0.640 | 0.850 (-0.028, 1.729) | 0.058 | 0.817 (-0.259, 1.893) | 0.137 |
| Model 4 | 0.272 (-0.003, 0.547) | 0.052 | reference | 0.352 (-0.346, 1.051) | 0.323 | 0.856 (0.111, 1.602) | 0.024 | 0.646 (-0.268, 1.559) | 0.166 |
| Standing long jump, cm |  |  |  |  |  |  |  |  |  |
| Model 1 | -1.296 (-3.130, 0.538) | 0.166 | reference | -2.403 (-7.009, 2.203) | 0.307 | -3.665 (-8.596, 1.266) | 0.145 | -4.059 (-10.095, 1.976) | 0.187 |
| Model 2 | -1.197 (-3.014, 0.620) | 0.197 | reference | -3.037 (-7.606, 1.532) | 0.193 | -3.691 (-8.569, 1.186) | 0.138 | -4.217 (-10.188, 1.754) | 0.166 |
| Model 3 | -1.194 (-2.978, 0.589) | 0.189 | reference | -3.371 (-7.850, 1.109) | 0.140 | -3.706 (-8.490, 1.078) | 0.129 | -4.520 (-10.377, 1.337) | 0.130 |
| Model 4 | -1.360 (-3.104, 0.384) | 0.126 | reference | -3.027 (-7.405, 1.351) | 0.175 | -3.693 (-8.366, 0.980) | 0.121 | -4.897 (-10.620, 0.826) | 0.094 |
| Handgrip strength, kg |  |  |  |  |  |  |  |  |  |
| Left hand |  |  |  |  |  |  |  |  |  |
| Model 1 | 0.059 (-0.251, 0.370) | 0.708 | reference | -0.782 (-1.562, -0.002) | 0.049 | -0.073 (-0.908, 0.762) | 0.864 | -0.305 (-1.327, 0.717) | 0.558 |
| Model 2 | 0.007 (-0.272, 0.287) | 0.959 | reference | -0.464 (-1.168, 0.240) | 0.197 | -0.060 (-0.811, 0.692) | 0.877 | -0.226 (-1.146, 0.694) | 0.630 |
| Model 3 | 0.037 (-0.259, 0.333) | 0.805 | reference | -0.579 (-1.324, 0.167) | 0.128 | -0.064 (-0.860, 0.732) | 0.874 | -0.208 (-1.183, 0.766) | 0.675 |
| Model 4 | -0.020 (-0.286, 0.247) | 0.885 | reference | -0.462 (-1.133, 0.209) | 0.177 | -0.060 (-0.776, 0.657) | 0.870 | -0.336 (-1.214, 0.541) | 0.452 |
| Right hand |  |  |  |  |  |  |  |  |  |
| Model 1 | 0.257 (-0.059, 0.573) | 0.111 | reference | -0.585 (-1.379, 0.210) | 0.149 | 0.422 (-0.429, 1.272) | 0.332 | 0.183 (-0.858, 1.224) | 0.731 |
| Model 2 | 0.200 (-0.079, 0.479) | 0.161 | reference | -0.237 (-0.943, 0.468) | 0.509 | 0.436 (-0.317, 1.189) | 0.256 | 0.269 (-0.652, 1.191) | 0.567 |
| Model 3 | 0.232 (-0.066, 0.531) | 0.127 | reference | -0.361 (-1.115, 0.392) | 0.348 | 0.431 (-0.374, 1.236) | 0.294 | 0.289 (-0.696, 1.275) | 0.565 |
| Model 4 | 0.171 (-0.093, 0.436) | 0.205 | reference | -0.236 (-0.904, 0.432) | 0.489 | 0.436 (-0.277, 1.149) | 0.231 | 0.152 (-0.721, 1.025) | 0.734 |

Model 1 adjusted for gender, age, caregivers’ smoking, breakfast, weekly nocturnal sleep time, physical activity time and dietary intake (vegetable, fruit, meat, coarse grain, milk, fish and soybean).

Model 2 adjusted for gender, age, caregivers’ smoking, breakfast, weekly nocturnal sleep time, physical activity time, weight and dietary intake (vegetable, fruit, meat, coarse grain, milk, fish and soybean) (not applicable to weight).

Model 3 adjusted for gender, age, caregivers’ smoking, breakfast, weekly nocturnal sleep time, physical activity time, BMI and dietary intake (vegetable, fruit, meat, coarse grain, milk, fish and soybean) (not applicable to BMI).

Model 4 adjusted for gender, age, caregivers’ smoking, breakfast, weekly nocturnal sleep time, physical activity time, weight, BMI and dietary intake (vegetable, fruit, meat, coarse grain, milk, fish and soybean) (not applicable to weight and BMI).

**Table S8. Association between fish intake and growth and development (*n* = 592)**

| **Growth and development** | **Fish intake, times/week** | | | | | | |
| --- | --- | --- | --- | --- | --- | --- | --- |
|  | **Total (*n* = 592)** | | **< 1 (*n* = 181)** | **1** - **2 (*n* = 245)** | | **> 2 (*n* = 166)** | |
|  | ***β* (95% *CI*)** | ***P*-Value** |  | ***β* (95% *CI*)** | ***P*-Value** | ***β* (95% *CI*)** | ***P*-Value** |
| Body height, cm |  |  |  |  |  |  |  |
| Model 1 | 0.197 (-0.513, 0.908) | 0.586 | reference | 0.641 (-0.617, 1.898) | 0.318 | 0.532 (-0.901, 1.965) | 0.467 |
| Model 2 | 0.129 (-0.414, 0.671) | 0.642 | reference | 0.618 (-0.345, 1.581) | 0.209 | 0.254 (-0.844, 1.352) | 0.651 |
| Model 3 | 0.178 (-0.491, 0.847) | 0.602 | reference | 0.717 (-0.470, 1.903) | 0.236 | 0.420 (-0.932, 1.772) | 0.542 |
| Model 4 | 0.052 (-0.166, 0.271) | 0.640 | reference | 0.129 (-0.258, 0.515) | 0.514 | 0.133 (-0.307, 0.573) | 0.554 |
| Weight, kg |  |  |  |  |  |  |  |
| Model 1 | 0.136 (-0.777, 1.050) | 0.770 | reference | 0.046 (-1.568, 1.659) | 0.956 | 0.555 (-1.283, 2.394) | 0.554 |
| Model 3 | 0.068 (-0.274, 0.410) | 0.696 | reference | 0.318 (-0.289, 0.924) | 0.304 | 0.155 (-0.536, 0.847) | 0.660 |
| BMI, kg/m^2^ |  |  |  |  |  |  |  |
| Model 1 | 0.029 (-0.332, 0.390) | 0.875 | reference | -0.116 (-0.754, 0.522) | 0.721 | 0.171 (-0.556, 0.898) | 0.645 |
| Model 2 | -0.021 (-0.156, 0.114) | 0.762 | reference | -0.133 (-0.373, 0.107) | 0.277 | -0.033 (-0.306, 0.241) | 0.814 |
| Sitting height, cm |  |  |  |  |  |  |  |
| Model 1 | 0.060 (-0.365, 0.485) | 0.782 | reference | 0.343 (-0.410, 1.096) | 0.372 | 0.200 (-0.658, 1.058) | 0.648 |
| Model 2 | 0.020 (-0.310, 0.350) | 0.905 | reference | 0.329 (-0.256, 0.915) | 0.270 | 0.037 (-0.631, 0.705) | 0.914 |
| Model 3 | 0.046 (-0.343, 0.436) | 0.816 | reference | 0.397 (-0.294, 1.088) | 0.260 | 0.120 (-0.668, 0.907) | 0.766 |
| Model 4 | -0.012 (-0.268, 0.243) | 0.925 | reference | 0.125 (-0.330, 0.580) | 0.591 | -0.014 (-0.532, 0.505) | 0.959 |
| Stature-sitting height index |  |  |  |  |  |  |  |
| Model 1 | -0.033 (-0.208, 0.142) | 0.712 | reference | 0.009 (-0.303, 0.321) | 0.953 | -0.057 (-0.412, 0.299) | 0.755 |
| Model 2 | -0.035 (-0.210, 0.140) | 0.693 | reference | 0.009 (-0.302, 0.319) | 0.957 | -0.066 (-0.420, 0.289) | 0.717 |
| Model 3 | -0.035 (-0.208, 0.138) | 0.689 | reference | 0.019 (-0.289, 0.326) | 0.904 | -0.071 (-0.421, 0.280) | 0.693 |
| Model 4 | -0.029 (-0.198, 0.141) | 0.741 | reference | 0.051 (-0.251, 0.352) | 0.742 | -0.055 (-0.399, 0.288) | 0.753 |
| Body fat rate, % |  |  |  |  |  |  |  |
| Model 1 | -0.261 (-1.124, 0.602) | 0.554 | reference | -0.264 (-1.786, 1.257) | 0.733 | -0.112 (-1.846, 1.621) | 0.899 |
| Model 2 | -0.354 (-0.950, 0.243) | 0.245 | reference | -0.295 (-1.347, 0.756) | 0.582 | -0.491 (-1.689, 0.707) | 0.422 |
| Model 3 | -0.317 (-0.829, 0.195) | 0.225 | reference | -0.041 (-0.941, 0.860) | 0.929 | -0.440 (-1.467, 0.586) | 0.400 |
| Model 4 | -0.306 (-0.815, 0.203) | 0.239 | reference | 0.010 (-0.886, 0.906) | 0.982 | -0.416 (-1.436, 0.605) | 0.425 |
| Fat weight, kg |  |  |  |  |  |  |  |
| Model 1 | -0.084 (-0.558, 0.390) | 0.728 | reference | -0.047 (-0.883, 0.790) | 0.913 | 0.053 (-0.901, 1.006) | 0.914 |
| Model 2 | -0.144 (-0.400, 0.113) | 0.274 | reference | -0.066 (-0.520, 0.388) | 0.775 | -0.189 (-0.707, 0.328) | 0.474 |
| Model 3 | -0.117 (-0.365, 0.132) | 0.357 | reference | 0.083 (-0.356, 0.522) | 0.710 | -0.138 (-0.638, 0.362) | 0.589 |
| Model 4 | -0.129 (-0.369, 0.111) | 0.291 | reference | 0.024 (-0.401, 0.448) | 0.913 | -0.167 (-0.650, 0.316) | 0.498 |
| Waist, cm |  |  |  |  |  |  |  |
| Model 1 | 0.156 (-0.719, 1.031) | 0.727 | reference | 0.120 (-1.427, 1.667) | 0.879 | 0.651 (-1.111, 2.414) | 0.469 |
| Model 2 | 0.043 (-0.396, 0.483) | 0.847 | reference | 0.082 (-0.692, 0.857) | 0.835 | 0.190 (-0.692, 1.073) | 0.672 |
| Model 3 | 0.096 (-0.347, 0.539) | 0.673 | reference | 0.363 (-0.418, 1.144) | 0.362 | 0.294 (-0.596, 1.184) | 0.517 |
| Model 4 | 0.065 (-0.351, 0.481) | 0.759 | reference | 0.222 (-0.512, 0.955) | 0.553 | 0.225 (-0.610, 1.060) | 0.598 |
| Chest, cm |  |  |  |  |  |  |  |
| Model 1 | 0.081 (-0.672, 0.835) | 0.833 | reference | 0.009 (-1.321, 1.339) | 0.989 | 0.493 (-1.023, 2.009) | 0.524 |
| Model 2 | -0.202 (-0.344, 0.304) | 0.903 | reference | -0.025 (-0.599, 0.549) | 0.933 | 0.080 (-0.574, 0.734) | 0.810 |
| Model 3 | 0.029 (-0.346, 0.403) | 0.881 | reference | 0.219 (-0.445, 0.883) | 0.518 | 0.185 (-0.572, 0.942) | 0.632 |
| Model 4 | -0.011 (-0.329, 0.308) | 0.948 | reference | 0.035 (-0.529, 0.599) | 0.903 | 0.095 (-0.548, 0.737) | 0.772 |
| Standing long jump, cm |  |  |  |  |  |  |  |
| Model 1 | 0.377 (-1.749, 2.503) | 0.728 | reference | -0.308 (-4.035, 3.419) | 0.871 | 0.372 (-3.876, 4.619) | 0.864 |
| Model 2 | 0.421 (-1.684, 2.526) | 0.695 | reference | -0.292 (-3.979, 3.394) | 0.876 | 0.560 (-3.643, 4.763) | 0.794 |
| Model 3 | 0.417 (-1.650, 2.484) | 0.693 | reference | -0.473 (-4.089, 3.144) | 0.798 | 0.613 (-3.508, 4.735) | 0.771 |
| Model 4 | 0.330 (-1.691, 2.350) | 0.749 | reference | -0.878 (-4.414, 2.658) | 0.627 | 0.415 (-3.611, 4.442) | 0.840 |
| Handgrip strength, kg |  |  |  |  |  |  |  |
| Left hand |  |  |  |  |  |  |  |
| Model 1 | 0.140 (-0.220, 0.500) | 0.446 | reference | 0.272 (-0.359, 0.904) | 0.397 | 0.452 (-0.267, 1.172) | 0.218 |
| Model 2 | 0.116 (-0.207, 0.440) | 0.481 | reference | 0.265 (-0.303, 0.833) | 0.361 | 0.358 (-0.290, 1.005) | 0.279 |
| Model 3 | 0.131 (-0.212, 0.474) | 0.454 | reference | 0.307 (-0.295, 0.909) | 0.317 | 0.401 (-0.284, 1.087) | 0.251 |
| Model 4 | 0.101 (-0.207, 0.410) | 0.520 | reference | 0.170 (-0.372, 0.712) | 0.540 | 0.334 (-0.283, 0.952) | 0.289 |
| Right hand |  |  |  |  |  |  |  |
| Model 1 | 0.186 (-0.180, 0.552) | 0.320 | reference | 0.441 (-0.202, 1.084) | 0.179 | 0.530 (-0.203, 1.263) | 0.156 |
| Model 2 | 0.160 (-0.163, 0.484) | 0.331 | reference | 0.433 (-0.136, 1.002) | 0.136 | 0.427 (-0.222, 1.076) | 0.197 |
| Model 3 | 0.176 (-0.170, 0.522) | 0.318 | reference | 0.479 (-0.129, 1.088) | 0.122 | 0.474 (-0.219, 1.168) | 0.180 |
| Model 4 | 0.144 (-0.162, 0.451) | 0.356 | reference | 0.331 (-0.208, 0.871) | 0.228 | 0.402 (-0.212, 1.016) | 0.200 |

Model 1 adjusted for gender, age, caregivers’ smoking, breakfast, weekly nocturnal sleep time, physical activity time and dietary intake (vegetable, fruit, meat, coarse grain, milk, egg and soybean).

Model 2 adjusted for gender, age, caregivers’ smoking, breakfast, weekly nocturnal sleep time, physical activity time, weight and dietary intake (vegetable, fruit, meat, coarse grain, milk, egg and soybean) (not applicable to weight).

Model 3 adjusted for gender, age, caregivers’ smoking, breakfast, weekly nocturnal sleep time, physical activity time, BMI and dietary intake (vegetable, fruit, meat, coarse grain, milk, egg and soybean) (not applicable to BMI).

Model 4 adjusted for gender, age, caregivers’ smoking, breakfast, weekly nocturnal sleep time, physical activity time, weight, BMI and dietary intake (vegetable, fruit, meat, coarse grain, milk, egg and soybean) (not applicable to weight and BMI).

**Table S9. Association between meat intake and growth and development among boys (n = 285)**

| **Growth and development** | **Meat intake, times/week** | | | | | | |
| --- | --- | --- | --- | --- | --- | --- | --- |
|  | **Total (*n* = 285)** | | **< 1 (*n* = 75)** | **1** - **2 (*n* = 87)** | | **> 2 (*n* = 123)** | |
|  | ***β* (95% *CI*)** | ***P*-Value** |  | ***β* (95% *CI*)** | ***P*-Value** | ***β* (95% *CI*)** | ***P*-Value** |
| Body height, cm |  |  |  |  |  |  |  |
| Model 1 | -0.175 (-1.121, 0.771) | 0.717 | reference | -0.753 (-2.746, 1.241) | 0.459 | -0.447 (-2.358, 1.464) | 0.647 |
| Model 2 | -0.511 (-1.235, 0.213) | 0.167 | reference | -0.346 (-1.866, 1.173) | 0.655 | -1.064 (-2.522,0.394) | 0.153 |
| Model 3 | -0.472 (-1.363, 0.418) | 0.299 | reference | -0.742 (-2.605, 1.121) | 0.435 | -1.035 (-2.831,0.760) | 0.258 |
| Model 4 | 0.201 (-0.076, 0.477) | 0.155 | reference | 0.760 (0.188, 1.333) | 0.009 | 0.459 (-0.093, 1.011) | 0.103 |
| Weight, kg |  |  |  |  |  |  |  |
| Model 1 | 0.722 (-0.590, 2.034) | 0.281 | reference | -0.867 (-3.622, 1.889) | 0.538 | 1.316 (-1.326, 3.958) | 0.329 |
| Model 3 | -0.373 (-0.842, 0.096) | 0.119 | reference | -0.828 (-1.806, 0.150) | 0.097 | -0.824 (-1.766, 0.119) | 0.087 |
| BMI, kg/m^2^ |  |  |  |  |  |  |  |
| Model 1 | 0.473 (-0.057, 1.004) | 0.080 | reference | -0.017 (-1.130, 1.096) | 0.976 | \| 0.924 (-0.143, 1.992) \| \| --- \| | 0.090 |
| Model 2 | 0.201 (0.012, 0.390) | 0.037 | reference | 0.311 (-0.085, 0.706) | 0.124 | 0.427 (0.048, 0.807) | 0.027 |
| Sitting height, cm |  |  |  |  |  |  |  |
| Model 1 | -0.224 (-0.796, 0.349) | 0.444 | reference | -1.064 (-2.268, 0.140) | 0.083 | -0.577 (-1.731, 0.577) | 0.327 |
| Model 2 | -0.418 (-0.869, 0.032) | 0.069 | reference | -0.829 (-1.772, 0.115) | 0.085 | **-0.934 (-1.840, -0.028)** | **0.043** |
| Model 3 | -0.427 (-0.955, 0.101) | 0.113 | reference | -1.056 (-2.158, 0.045) | 0.060 | -0.980 (-2.042, 0.081) | 0.070 |
| Model 4 | -0.111 (-0.458, 0.237) | 0.533 | reference | -0.356 (-1.086, 0.374) | 0.340 | -0.283 (-0.987, 0.420) | 0.430 |
| Stature-sitting height index |  |  |  |  |  |  |  |
| Model 1 | -0.100 (-0.337, 0.138) | 0.412 | reference | -0.496 (-0.995, 0.003 | 0.051 | -0.259 (-0.737, 0.219) | 0.288 |
| Model 2 | -0.109 (-0.347, 0.128) | 0.368 | reference | -0.484 (-0.982, 0.014) | 0.057 | -0.276 (-0.754, 0.202) | 0.257 |
| Model 3 | -0.131 (-0.367, 0.105) | 0.277 | reference | **-0.495 (-0.988, -0.002)** | **0.049** | -0.322 (-0.797, 0.153) | 0.184 |
| Model 4 | -0.166 (-0.399, 0.067) | 0.162 | reference | -0.577 (-1.063, -0.091) | 0.020 | -0.404 (-0.872, 0.065) | 0.091 |
| Body fat rate, % |  |  |  |  |  |  |  |
| Model 1 | **1.231 (0.126, 2.337)** | **0.029** | reference | 0.035 (-2.259, 2.329) | 0.976 | 2.047 (-0.153, 4.247) | 0.068 |
| Model 2 | 0.822 (0.002, 1.642) | 0.049 | reference | 0.521 (-1.176, 2.218) | 0.547 | 1.309 (-0.319, 2.937) | 0.115 |
| Model 3 | 0.485 (-0.242, 1.213) | 0.191 | reference | 0.061 (-1.434, 1.557) | 0.936 | 0.602 (-0.839, 2.043) | 0.413 |
| Model 4 | 0.401 (-0.322, 1.124) | 0.277 | reference | -0.133 (-1.617, 1.352) | 0.861 | 0.409 (-1.022, 1.840) | 0.575 |
| Fat weight, kg |  |  |  |  |  |  |  |
| Model 1 | 0.564 (-0.051, 1.178) | 0.072 | reference | -0.306 (-1.585, 0.973) | 0.639 | 0.942 (-0.285, 2.168) | 0.132 |
| Model 2 | 0.289 (-0.069, 0.646) | 0.113 | reference | 0.023 (-0.716, 0.761) | 0.952 | 0.443 (-0.266, 1.152) | 0.221 |
| Model 3 | 0.106 (-0.234, 0.447) | 0.541 | reference | -0.290 (-0.994, 0.414) | 0.420 | 0.055 (-0.624, 0.733) | 0.874 |
| Model 4 | 0.154 (-0.182, 0.491) | 0.369 | reference | -0.179 (-0.874, 0.516) | 0.614 | 0.165 (-0.505, 0.835) | 0.630 |
| Waist, cm |  |  |  |  |  |  |  |
| Model 1 | **1.430 (0.141, 2.720)** | **0.030** | reference | 0.402 (-2.297, 3.101) | 0.771 | **2.718 (0.130, 5.305)** | **0.040** |
| Model 2 | **0.795 (0.220, 1.370)** | **0.007** | reference | 1.165 (-0.021, 2.350) | 0.054 | **1.559 (0.422, 2.697)** | **0.007** |
| Model 3 | 0.396 (-0.172, 0.964) | 0.172 | reference | 0.438 (-0.743, 1.620) | 0.467 | 0.702 (-0.436, 1.840) | 0.227 |
| Model 4 | 0.556 (0.023, 1.089) | 0.041 | reference | 0.811 (-0.290, 1.913) | 0.149 | 1.073 (0.011, 2.135) | 0.048 |
| Chest, cm |  |  |  |  |  |  |  |
| Model 1 | 1.006 (-0.022, 2.035) | 0.055 | reference | -0.102 (-2.260, 2.056) | 0.926 | 1.915 (-0.154, 3.983) | 0.070 |
| Model 2 | **0.498 (0.047, 0.949)** | **0.031** | reference | 0.508 (-0.437, 1.454) | 0.292 | **0.988 (0.081, 1.896)** | **0.033** |
| Model 3 | 0.198 (-0.292, 0.687) | 0.429 | reference | -0.073 (-1.105, 0.958) | 0.889 | 0.340 (-0.654, 1.335) | 0.502 |
| Model 4 | 0.373 (-0.066, 0.812) | 0.095 | reference | 0.327 (-0.594, 1.249) | 0.486 | 0.739 (-0.149, 1.627) | 0.103 |
| Standing long jump, cm |  |  |  |  |  |  |  |
| Model 1 | **-4.036 (-7.049, -1.024)** | **0.009** | reference | -1.635 (-7.813, 4.543) | 0.604 | \| **-8.691 (-14.614, -2.768)** \| \| --- \| | **0.004** |
| Model 2 | **-3.658 (-6.596, -0.719)** | **0.015** | reference | -2.085 (-8.099, 3.929) | 0.497 | **-8.008 (-13,779, -2.237)** | **0.007** |
| Model 3 | **-3.170 (-6.037, -0.304)** | **0.030** | reference | -1.665 (-7.513, 4.182) | 0.577 | **-7.036 (-12.670, -1.402)** | **0.014** |
| Model 4 | -2.679 (-5.490, 0.132) | 0.062 | reference | -0.632 (-6.378, 5.114) | 0.829 | -6.008 (-11.547, -0.470) | 0.033 |
| Handgrip strength, kg |  |  |  |  |  |  |  |
| Left hand |  |  |  |  |  |  |  |
| Model 1 | -0.153 (-0.639, 0.334) | 0.538 | reference | **-1.277 (-2.273, -0.280)** | **0.012** | -0.564 (-1.520, 0.391) | 0.247 |
| Model 2 | -0.267 (-0.708, 0.174) | 0.236 | reference | **-1.145 (-2.050, -0.240)** | **0.013** | -0.765 (-1.633, 0.104) | 0.084 |
| Model 3 | -0.278 (-0.746, 0.191) | 0.245 | reference | **-1.272 (-2.227, -0.317)** | **0.009** | -0.801 (-1.721, 0.120) | 0.088 |
| Model 4 | -0.106 (-0.523, 0.312) | 0.619 | reference | -0.907 (-1.764, -0.051) | 0.038 | -0.437 (-1.263, 0.388) | 0.299 |
| Right hand |  |  |  |  |  |  |  |
| Model 1 | -0.218 (-0.732, 0.297) | 0.407 | reference | **-1.147 (-2.204, -0.090)** | **0.033** | -0.695 (-1.708, 0.319) | 0.179 |
| Model 2 | -0.344(-0.805, 0.117) | 0.143 | reference | **-1.000 (-1.947, -0.052)** | **0.039** | **-0.919 (-1.828, 0.009)** | **0.048** |
| Model 3 | -0.360 (-0.851, 0.132) | 0.152 | reference | **-1.142 (-2.148, -0.136)** | **0.026** | -0.964 (-1.934, 0.005) | 0.051 |
| Model 4 | -0.177 (-0.613, 0.260) | 0.427 | reference | -0.747 (-1.642, 0.148) | 0.102 | -0.571 (-1.434, 0.292) | 0.194 |

Model 1 adjusted for age, caregivers’ smoking, breakfast, weekly nocturnal sleep time, physical activity time and dietary intake (vegetable, fruit, coarse grain, milk, egg, fish and soybean).

Model 2 adjusted for age, caregivers’ smoking, breakfast, weekly nocturnal sleep time, physical activity time, weight and dietary intake (vegetable, fruit, coarse grain, milk, egg, fish and soybean) (not applicable to weight).

Model 3 adjusted for age, caregivers’ smoking, breakfast, weekly nocturnal sleep time, physical activity time, BMI and dietary intake (vegetable, fruit, coarse grain, milk, egg, fish and soybean) (not applicable to BMI).

Model 4 adjusted for age, caregivers’ smoking, breakfast, weekly nocturnal sleep time, physical activity time, weight, BMI and dietary intake (vegetable, fruit, coarse grain, milk, egg, fish and soybean) (not applicable to weight and BMI).

**Table S10. Association between meat intake and growth and development among girls (n = 307)**

| **Growth and development** | **Meat intake, times/week** | | | | | | |
| --- | --- | --- | --- | --- | --- | --- | --- |
|  | **Total (*n* = 307)** | | **< 1 (*n* = 67)** | **1** - **2 (*n* = 117)** | | **> 2 (*n* = 123)** | |
|  | ***β* (95% *CI*)** | ***P*-Value** |  | ***β* (95% *CI*)** | ***P*-Value** | ***β* (95% *CI*)** | ***P*-Value** |
| Body height, cm |  |  |  |  |  |  |  |
| Model 1 | -0.377 (-1.334, 0.581) | 0.441 | reference | -1.509 (-3.435, 0.418) | 0.125 | -0.974 (-2.923, 0.975) | 0.327 |
| Model 2 | 0.067 (-0.661, 0.794) | 0.858 | reference | -0.858 (-2.325, 0.610) | 0.252 | -0.140 (-1.626, 1.346) | 0.854 |
| Model 3 | -0.179 (-1.087, 0.729) | 0.700 | reference | -1.365 (-3.192, 0.463) | 0.143 | -0.651 (-2.502, 1.200) | 0.491 |
| Model 4 | 0.084 (-0.222, 0.390) | 0.590 | reference | -0.061 (-0.682, 0.560) | 0.847 | 0.134 (-0.494, 0.761) | 0.676 |
| Weight, kg |  |  |  |  |  |  |  |
| Model 1 | -0.797 (-1.921, 0.328) | 0.165 | reference | -1.180 (-3.448, 1.087) | 0.308 | \| -1.512 (-3.806, 0.782) \| \| --- \| | 0.196 |
| Model 3 | -0.139 (-0.590, 0.313) | 0.547 | reference | -0.691 (-1.602, 0.220) | 0.137 | -0.416 (-1.339, 0.507) | 0.377 |
| BMI, kg/m^2^ |  |  |  |  |  |  |  |
| Model 1 | -0.274 (-0.703, 0.155) | 0.211 | reference | -0.204 (-1.071, 0.662) | 0.644 | -0.457 (-1.334, 0.419) | 0.306 |
| Model 2 | 0.005 (-0.168, 0.177) | 0.959 | reference | 0.209 (-0.140, 0.557) | 0.241 | 0.072 (-0.281, 0.425) | 0.691 |
| Sitting height, cm |  |  |  |  |  |  |  |
| Model 1 | -0.321 (-0.888, 0.246) | 0.267 | reference | -0.771 (-1.922, 0.380) | 0.189 | -0.775 (-1.939, 0.390) | 0.192 |
| Model 2 | -0.059 (-0.490, 0.373) | 0.789 | reference | -0.381 (-1.257, 0.495) | 0.394 | -0.275 (-1.162, 0.612) | 0.543 |
| Model 3 | -0.172 (-0.691, 0.346) | 0.515 | reference | -0.660 (-1.710, 0.391) | 0.218 | -0.525 (-1.590, 0.539) | 0.333 |
| Model 4 | -0.052 (-0.392, 0.288) | 0.765 | reference | -0.062 (-0.758, 0.634) | 0.862 | -0.165 (-0.869, 0.538) | 0.645 |
| Stature-sitting height index |  |  |  |  |  |  |  |
| Model 1 | -0.074 (-0.307, 0.159) | 0.534 | reference | 0.017 (-0.459, 0.493) | 0.944 | -0.162 (-0.644, 0.319) | 0.509 |
| Model 2 | -0.058 (-0.290, 0.175) | 0.626 | reference | 0.044 (-0.430, 0.518) | 0.854 | -0.127 (-0.607, 0.353) | 0.603 |
| Model 3 | -0.045 (-0.273, 0.184) | 0.702 | reference | 0.040 (-0.425, 0.506) | 0.865 | -0.110 (-0.582, 0.362) | 0.648 |
| Model 4 | -0.059 (-0.283, 0.164) | 0.603 | reference | -0.032 (-0.489, 0.426) | 0.892 | -0.153 (-0.616, 0.309) | 0.515 |
| Body fat rate, % |  |  |  |  |  |  |  |
| Model 1 | -0.431 (-1.619, 0.756) | 0.477 | reference | -0.586 (-2.996, 1.823) | 0.633 | -0.741 (-3.179, 1.696) | 0.551 |
| Model 2 | 0.240 (-0.479, 0.959) | 0.513 | reference | 0.427 (-0.995, 1.849) | 0.556 | 0.557 (-0.883, 1.997) | 0.449 |
| Model 3 | 0.245 (-0.293, 0.783) | 0.371 | reference | -0.075 (-1.126, 0.976) | 0.889 | 0.404 ((-0.661, 1.468) | 0.458 |
| Model 4 | 0.227 (-0.308, 0.762) | 0.405 | reference | -0.149 (-1.199, 0.902) | 0.781 | 0.359 (-0.702, 1.421) | 0.507 |
| Fat weight, kg |  |  |  |  |  |  |  |
| Model 1 | -0.387 (-1.035, 0.260) | 0.241 | reference | -0.435 (-1.751, 0.881) | 0.517 | -0.679 (-2.010, 0.653) | 0.318 |
| Model 2 | 0.020 (-0.278, 0.318) | 0.894 | reference | 0.177 (-0.414, 0.769) | 0.557 | 0.106 (-0.493, 0.704) | 0.730 |
| Model 3 | -0.015 (-0.299, 0.268) | 0.915 | reference | -0.155 (-0.724, 0.414) | 0.593 | -0.052 (-0.629, 0.524) | 0.859 |
| Model 4 | 0.017 (-0.247, 0.281) | 0.902 | reference | 0.013 (-0.512, 0.539) | 0.960 | 0.049 (-0.482, 0.581) | 0.856 |
| Waist, cm |  |  |  |  |  |  |  |
| Model 1 | -0.609 (-1.615, 0.398) | 0.236 | reference | -1.696 (-3.731, 0.339) | 0.102 | -1.403 (-3.461, 0.656) | 0.182 |
| Model 2 | -0.017 (-0.580, 0.546) | 0.953 | reference | -0.811 (-1.933, 0.310) | 0.156 | -0.270 (-1.406, 0.866) | 0.642 |
| Model 3 | -0.089 (-0.684, 0.505) | 0.768 | reference | **-1.307 (-2.502, -0.112)** | **0.032** | -0.533 (-1.743, 0.677) | 0.388 |
| Model 4 | -0.020 (-0.570, 0.530) | 0.943 | reference | -0.948 (-2.049, 0.153) | 0.091 | -0.317 (-1.429, 0.796) | 0.577 |
| Chest, cm |  |  |  |  |  |  |  |
| Model 1 | -0.071 (-1.043, 0.901) | 0.886 | reference | -0.712 (-2.678, 1.254) | 0.478 | \| -0.101 (-2.090, 1.889) \| \| --- \| | 0.921 |
| Model 2 | **0.556 (0.150, 0.961)** | **0.007** | reference | 0.220 (-0.595, 1.035) | 0.597 | **1.093 (0.268, 1.919)** | **0.009** |
| Model 3 | 0.460 (-0.046, 0.966) | 0.075 | reference | -0.316 (-1.341, 0.709) | 0.545 | 0.785 (-0.253, 1.824) | 0.138 |
| Model 4 | 0.554 (0.152, 0.957) | 0.007 | reference | 0.162 (-0.649, 0.973) | 0.696 | 1.073 (0.253, 1.893) | 0.010 |
| Standing long jump, cm |  |  |  |  |  |  |  |
| Model 1 | -1.632 (-4.240, 0.977) | 0.220 | reference | 0.024 (-5.172, 5.220) | 0.993 | -2.874 (-8.132, 2.383) | 0.284 |
| Model 2 | -1.656 (-4.272, 0.960) | 0.215 | reference | -0.032 (-5.236, 5.172) | 0.990 | -2.946 (-8.216, 2.325) | 0.273 |
| Model 3 | -1.797 (-4.400, 0.805) | 0.176 | reference | -0.111 (-5.277, 5.056) | 0.966 | -3.176 (-8.410, 2.058) | 0.234 |
| Model 4 | -1.641 (-4.194,0.912 ) | 0.208 | reference | 0.675 (-4.405,5.755 ) | 0.794 | -2.703 (-7.837, 2.431) | 0.302 |
| Handgrip strength, kg |  |  |  |  |  |  |  |
| Left hand |  |  |  |  |  |  |  |
| Model 1 | -0.342 (-0.814, 0.130) | 0.156 | reference | -0.758 (-1.703, 0.188) | 0.116 | -0.817 (-1.773, 0.139) | 0.094 |
| Model 2 | -0.188 (-0.609, 0.233) | 0.381 | reference | -0.530 (-1.370, 0.309) | 0.216 | -0.525 (-1.375, 0.325) | 0.226 |
| Model 3 | -0.242 (-0.689, 0.205) | 0.288 | reference | -0.684 (-1.576, 0.208) | 0.133 | -0.651 (-1.555, 0.252) | 0.158 |
| Model 4 | -0.185 (-0.592, 0.221) | 0.372 | reference | -0.400 (-1.213, 0.413) | 0.334 | -0.481 (-1.302, 0.341) | 0.252 |
| Right hand |  |  |  |  |  |  |  |
| Model 1 | **-0.512 (-0.973, -0.050)** | **0.030** | reference | -0.434 (-1.350, 0.483) | 0.354 | **-1.025 (-1.951, -0.098)** | **0.030** |
| Model 2 | -0.348 (-0.749, 0.053) | 0.089 | reference | -0.192 (-0.983, 0.599) | 0.635 | -0.715 (-1.516, 0.086) | 0.080 |
| Model 3 | -0.405 (-0.837, 0.026) | 0.066 | reference | -0.354 (-1.207, 0.498) | 0.415 | -0.847 (-1.711, 0.016) | 0.054 |
| Model 4 | -0.345 (-0.730, 0.004) | 0.087 | reference | -0.058 (-0.818, 0.703) | 0.881 | -0.669 (-1.437, 0.1) | 0.088 |

Model 1 adjusted for age, caregivers’ smoking, breakfast, weekly nocturnal sleep time, physical activity time and dietary intakes (vegetable, fruit, coarse grain, milk, egg, fish and soybean).

Model 2 adjusted for age, caregivers’ smoking, breakfast, weekly nocturnal sleep time, physical activity time, weight and dietary intakes (vegetable, fruit, coarse grain, milk, egg, fish and soybean) (not applicable to weight).

Model 3 adjusted for age, caregivers’ smoking, breakfast, weekly nocturnal sleep time, physical activity time, BMI and dietary intakes (vegetable, fruit, coarse grain, milk, egg, fish and soybean) (not applicable to BMI).

Model 4 adjusted for age, caregivers’ smoking, breakfast, weekly nocturnal sleep time, physical activity time, weight, BMI and dietary intakes (vegetable, fruit, coarse grain, milk, egg, fish and soybean) (not applicable to weight and BMI).

**Table S11. Association between milk intake and growth and development among boys (n = 285)**

| **Growth and development** | **Milk intake, times/week** | | | | | | |
| --- | --- | --- | --- | --- | --- | --- | --- |
|  | **Total (*n* = 285)** | | **< 3 (*n* = 108)** | **3** - **5 (*n* = 123)** | | **> 5 (*n* = 54)** | |
|  | ***β* (95% *CI*)** | ***P*-Value** |  | ***β* (95% *CI*)** | ***P*-Value** | ***β* (95% *CI*)** | ***P*-Value** |
| Body height, cm |  |  |  |  |  |  |  |
| Model 1 | -0.023 (-1.106, 1.060) | 0.967 | reference | -0.045 (-1.763, 1.673) | 0.959 | -0.434 (-2.692, 1.823) | 0.706 |
| Model 2 | 0.302 (-0.526, 1.131) | 0.474 | reference | 0.651 (-0.661, 1.963) | 0.331 | 0.349 (-1.374, 2.071) | 0.692 |
| Model 3 | 0.191 (-0.825, 1.206) | 0.713 | reference | 0.358 (-1.252, 1.969) | 0.663 | 0.021 (-2.094,2.135) | 0.985 |
| Model 4 | 0.032 (-0.283, 0.346) | 0.843 | reference | 0.389 (-0.103, 0.882) | 0.121 | 0.049 (-0.598, 0.695) | 0.883 |
| Weight, kg |  |  |  |  |  |  |  |
| Model 1 | -0.699 (-2.200, 0.802) | 0.362 | reference | -1.486 (-3.860, 0.889) | 0.220 | -1.670 (-4.790, 1.451) | 0.294 |
| Model 3 | 0.008 (-0.447, 0.623) | 0.747 | reference | -0.017 (-0.862, 0.828) | 0.968 | -0.015 (-1.125, 1.094) | 0.978 |
| BMI, kg/m^2^ |  |  |  |  |  |  |  |
| Model 1 | -0.340 (-0.947, 0.267) | 0.272 | reference | -0.634 (-1.594, 0.325) | 0.195 | -0.715 (-1.975, 0.546) | 0.266 |
| Model 2 | -0.076 (-0.292, 0.140) | 0.489 | reference | -0.73 (-0.415, 0.268) | 0.673 | -0.084 (-0.532, 0.364) | 0.713 |
| Sitting height, cm |  |  |  |  |  |  |  |
| Model 1 | -0.186 (-0.840, 0.469) | 0.579 | reference | -0.562 (-1.599, 0.476) | 0.289 | -0.484 (-1.847, 0.879) | 0.486 |
| Model 2 | 0.003 (-0.512, 0.518) | 0.991 | reference | -0.158 (-0.973, 0.657) | 0.703 | -0.031 (-1.101, 1.039) | 0.954 |
| Model 3 | -0.039 (-0.587, 0.351) | 0.898 | reference | -0.285 (-1.236, 0.667) | 0.558 | -0.172 (-1.422, 1.077) | 0.787 |
| Model 4 | -0.114 (-0.509, 0.281) | 0.571 | reference | -0.270 (-0.898, 0.358) | 0.399 | -0.159 (-0.984, 0.665) | 0.705 |
| Stature-sitting height index |  |  |  |  |  |  |  |
| Model 1 | -0.106 (-0.378, 0.166) | 0.446 | reference | -0.363 (-0.793, 0.067) | 0.098 | -0.139 (-0.704, 0.426) | 0.629 |
| Model 2 | -0.097 (-0.368, 0.175) | 0.486 | reference | -0.344 (-0.774, 0.086) | 0.117 | -0.117 (-0.682, 0.447) | 0.684 |
| Model 3 | -0.083 (-0.353, 0.186) | 0.545 | reference | -0.320 (-0.746, 0.106) | 0.141 | -0.091 (-0.650, 0.469) | 0.751 |
| Model 4 | -0.075 (-0.340, 0.190) | 0.580 | reference | -0.322 (-0.740, 0.096) | 0.131 | -0.092 (-0.641, 0.457) | 0.742 |
| Body fat rate, % |  |  |  |  |  |  |  |
| Model 1 | 0.205 (-1.060, 1.470) | 0.750 | reference | -1.218 (-3.195, 0.760) | 0.227 | 0.825 (-1.773, 3.423) | 0.534 |
| Model 2 | 0.602 (-0.336, 1.539) | 0.209 | reference | -0.384 (-1.849, 1.081) | 0.607 | 1.761 (-0.162, 3.685) | 0.073 |
| Model 3 | 0.742 (-0.088, 1.527) | 0.080 | reference | -0.226 (-1.518, 1.067) | 0.732 | **1.942 (0.245, 3.639)** | **0.025** |
| Model 4 | 0.762 (-0.059, 1.583) | 0.069 | reference | -0.230 (-1.507, 1.047) | 0.724 | 1.939 (0.262, 3.616) | 0.023 |
| Fat weight, kg |  |  |  |  |  |  |  |
| Model 1 | -0.026 (-0.729, 0.677) | 0.943 | reference | -0.528 (-1.630, 0.574) | 0.348 | 0.024 (-1.425, 1.472) | 0.974 |
| Model 2 | 0.241 (-0.168, 0.649) | 0.248 | reference | 0.035 (-0.602, 0.673) | 0.914 | 0.657 (-0.180, 1.494) | 0.124 |
| Model 3 | 0.303 (-0.085, 0.692) | 0.126 | reference | 0.081 (-0.528, 0.689) | 0.795 | 0.710 (-0.090, 1.509) | 0.082 |
| Model 4 | 0.292 (-0.090, 0.674) | 0.135 | reference | 0.083 (-0.515, 0.681) | 0.786 | 0.712 (-0.074, 1.497) | 0.076 |
| Waist, cm |  |  |  |  |  |  |  |
| Model 1 | 0.408 (-1.067, 1.884) | 0.588 | reference | -0.588 (-2.914, 1.737) | 0.620 | 0.798 (-2.258, 3.854) | 0.609 |
| Model 2 | **1.023 (0.366, 1.681)** | **0.002** | reference | 0.719 (-0.305, 1.743) | 0.169 | **2.267 (0.923, 3.612)** | **0.001** |
| Model 3 | **1.152 (0.504, 1.800)** | **< 0.001** | reference | 0.795 (-0.226, 1.816) | 0.127 | **2.356 (1.016, 3.697)** | **0.001** |
| Model 4 | 1.114 (0.509, 1.720) | < 0.001 | reference | 0.803 (-0.145, 1.750) | 0.097 | 2.363 (1.119, 3.607) | < 0.001 |
| Chest, cm |  |  |  |  |  |  |  |
| Model 1 | -0.098 (-1.275, 1.078) | 0.870 | reference | -0.730 (-2.589, 1.130) | 0.442 | -0.344 (-2.787, 2.100) | 0.783 |
| Model 2 | 0.394 (-0.121, 0.910) | 0.134 | reference | 0.316 (-0.500, 1.133) | 0.448 | 0.832 (-0.204, 1.904) | 0.128 |
| Model 3 | 0.483 (-0.076, 1.041) | 0.090 | reference | 0.351 (-0.541, 1.242) | 0.441 | 0.874 (-0.297, 2.044) | 0.144 |
| Model 4 | 0.441 (-0.057, 0.940) | 0.083 | reference | 0.359 (-0.433, 1.151) | 0.374 | 0.881 (-0.159, 1.921) | 0.097 |
| Standing long jump, cm |  |  |  |  |  |  |  |
| Model 1 | 1.496 (-1.950, 4.943) | 0.395 | reference | 3.620 (-1.704, 8.944) | 0.183 | 4.089 (-2.906, 11.085) | 0.252 |
| Model 2 | 1.130 (-2.230, 4.490) | 0.510 | reference | 2.849 (-2.344, 8.042) | 0.282 | 3.223 (-3.596, 10.041) | 0.354 |
| Model 3 | 0.874 (-2.396, 4.143) | 0.600 | reference | 2.484 (-2.570, 7.538) | 0.335 | 2.810 (-3.826, 9.446) | 0.407 |
| Model 4 | 0.758 (-2.435, 3.951) | 0.642 | reference | 2.505 (-2.437, 7.448) | 0.320 | 2.829 (-3.661, 9.319) | 0.393 |
| Handgrip strength, kg |  |  |  |  |  |  |  |
| Left hand |  |  |  |  |  |  |  |
| Model 1 | 0.019 (-0.538, 0.575) | 0.947 | reference | -0.071 (-0.930, 0.788) | 0.872 | -0.256 (-1.385, 0.872) | 0.657 |
| Model 2 | 0.129 (-0.375, 0.634) | 0.616 | reference | 0.155 (-0.626, 0.936) | 0.697 | -0.002 (-1.028, 1.024) | 0.997 |
| Model 3 | 0.109 (-0.426, 0.643) | 0.690 | reference | 0.091 (-0.734, 0.917) | 0.828 | -0.073 (-1.157, 1.011) | 0.895 |
| Model 4 | 0.068 (-0.406, 0.542) | 0.779 | reference | 0.099 (-0.638, 0.836) | 0.792 | -0.067 (-1.034, 0.901) | 0.893 |
| Right hand |  |  |  |  |  |  |  |
| Model 1 | -0.031 (-0.619, 0.558) | 0.919 | reference | -0.011 (-0.922, 0.899) | 0.980 | -0.379 (-1.576, 0.818) | 0.535 |
| Model 2 | 0.092 (-0.435, 0.619) | 0.732 | reference | 0.242 (-0.577, 1.060) | 0.563 | -0.095 (-1.169, 0.980) | 0.863 |
| Model 3 | 0.071 (-0.489, 0.632) | 0.803 | reference | 0.174 (-0.696, 1.043) | 0.695 | -0.170 (-1.312, 0.971) | 0.770 |
| Model 4 | 0.028 (-0.467, 0.524) | 0.911 | reference | 0.182 (-0.588, 0.952) | 0.643 | -0.163 (-1.174, 0.848) | 0.752 |

Model 1 adjusted for age, caregivers’ smoking, breakfast, weekly nocturnal sleep time, physical activity time and dietary intake (vegetable, fruit, coarse grain, meat, egg, fish and soybean).

Model 2 adjusted for age, caregivers’ smoking, breakfast, weekly nocturnal sleep time, physical activity time, weight and dietary intake (vegetable, fruit, coarse grain, meat, egg, fish and soybean) (not applicable to weight).

Model 3 adjusted for age, caregivers’ smoking, breakfast, weekly nocturnal sleep time, physical activity time, BMI and dietary intake (vegetable, fruit, coarse grain, meat, egg, fish and soybean) (not applicable to BMI).

Model 4 adjusted for age, caregivers’ smoking, breakfast, weekly nocturnal sleep time, physical activity time, weight, BMI and dietary intake (vegetable, fruit, coarse grain, meat, egg, fish and soybean) (not applicable to weight and BMI).

**Table S12. Association between milk intake and growth and development among girls (n = 307)**

| **Growth and development** | **Milk intake, times/week** | | | | | | |
| --- | --- | --- | --- | --- | --- | --- | --- |
|  | **Total (*n* = 307)** | | **< 3 (*n* = 80)** | **3** - **5 (*n* = 183)** | | **> 5 (*n* = 44)** | |
|  | ***β* (95% *CI*)** | ***P*-Value** |  | ***β* (95% *CI*)** | ***P*-Value** | ***β* (95% *CI*)** | ***P*-Value** |
| Body height, cm |  |  |  |  |  |  |  |
| Model 1 | -1.010 (-2.157, 0.138) | 0.085 | reference | -1.044 (-2.733, 0.644) | 0.225 | **-2.533 (-4.850, -0.216)** | **0.032** |
| Model 2 | -0.126 (-1.003, 0.751) | 0.778 | reference | 0.131 (-1.163, 1.424) | 0.843 | -0.603 (-2.383, 1.178) | 0.507 |
| Model 3 | -0.614 (-1.708, 0.479) | 0.271 | reference | -0.464 (-2.077, 1.149) | 0.573 | -1.730 (-3.943, 0.484) | 0.126 |
| Model 4 | -0.097 (-0.465, 0.272) | 0.607 | reference | -0.158 (-0.705, 0.388) | 0.570 | -0.270 (-1.021, 0.482) | 0.482 |
| Weight, kg |  |  |  |  |  |  |  |
| Model 1 | **-1.589 (-2.937, -0.241)** | **0.021** | reference | **-2.129 (-4.117, -0.142)** | **0.036** | **-3.499 (-6.226, -0.772)** | **0.012** |
| Model 3 | -0.273 (-0.817, 0.270) | 0.325 | reference | -0.162 (-0.966, 0.642) | 0.693 | -0.773 (-1.877, 0.330) | 0.170 |
| BMI, kg/m^2^ |  |  |  |  |  |  |  |
| Model 1 | **-0.548 (-1.062, -0.034)** | **0.037** | reference | **-0.821 (-1.580, -0.062)** | **0.034** | **-1.137 (-2.179, -0.095)** | **0.032** |
| Model 2 | 0.008 (-0.200, 0.215) | 0.942 | reference | -0.076 (-0.383, 0.231) | 0.629 | 0.087 (-0.336, 0.510) | 0.686 |
| Sitting height, cm |  |  |  |  |  |  |  |
| Model 1 | **-0.950 (-1.630, -0.270)** | **0.006** | reference | -0.782 (-1.791, 0.227) | 0.129 | **-2.158 (-3.543, -0.774)** | **0.002** |
| Model 2 | -0.427 (-0.947, 0.093) | 0.107 | reference | -0.078 (-0.850, 0.694) | 0.842 | -1.003 (-2.065, 0.060) | 0.064 |
| Model 3 | **-0.652 (-1.277, -0.028)** | **0.041** | reference | -0.335 (-1.262, 0.593) | 0.479 | **-1.539 (-2.811, -0.267)** | **0.018** |
| Model 4 | -0.416 (-0.826, -0.005) | 0.047 | reference | -0.194 (-0.807, 0.418) | 0.534 | -0.869 (-1.712, -0.027) | 0.043 |
| Stature-sitting height index |  |  |  |  |  |  |  |
| Model 1 | -0.267 (-0.546, 0.012) | 0.060 | reference | -0.137 (-0.555, 0.280) | 0.518 | -0.521 (-1.093, 0.052) | 0.075 |
| Model 2 | -0.235 (-0.515, 0.045) | 0.100 | reference | -0.088 (-0.506, 0.329) | 0.678 | -0.440 (-1.015, 0.135) | 0.134 |
| Model 3 | -0.209 (-0.485, 0.067) | 0.137 | reference | -0.044 (-0.455, 0.367) | 0.835 | -0.391 (-0.955, 0.173) | 0.174 |
| Model 4 | -0.238 (-0.508, 0.032) | 0.084 | reference | -0.061 (-0.463, 0.342) | 0.767 | -0.472 (-1.026, 0.082) | 0.095 |
| Body fat rate, % |  |  |  |  |  |  |  |
| Model 1 | -1.225 (-2.648, 0.199) | 0.092 | reference | **-2.353 (-4.464, -0.241)** | **0.029** | -2.179 (-5.077, 0.719) | 0.141 |
| Model 2 | 0.113 (-0.753, 0.980) | 0.798 | reference | -0.524 (-1.778, 0.729) | 0.412 | 0.825 (-0.901, 2.550) | 0.349 |
| Model 3 | 0.128 (-0.520, 0.776) | 0.699 | reference | -0.298 (-1.226, 0.629) | 0.529 | 0.667 (-0.606, 1.940) | 0.305 |
| Model 4 | 0.092 (-0.553, 0.737) | 0.780 | reference | -0.315 (-1.239, 0.608) | 0.503 | 0.584 (-0.687, 1.856) | 0.368 |
| Fat weight, kg |  |  |  |  |  |  |  |
| Model 1 | **-0.948 (-1.724, -0.172)** | **0.017** | reference | **-1.477 (-2.630, -0.323)** | **0.012** | **-1.843 (-3.426, -0.260)** | **0.022** |
| Model 2 | -0.135 (-0.494, 0.224) | 0.461 | reference | -0.372 (-0.893, 0.149) | 0.162 | -0.028 (-0.746, 0.689) | 0.938 |
| Model 3 | -0.204 (-0.546, 0.137) | 0.241 | reference | -0.352 (-0.854, 0.150) | 0.169 | -0.286 (-0.975, 0.404) | 0.416 |
| Model 4 | -0.141 (-0.460, 0.177) | 0.385 | reference | -0.313 (-0.775, 0.150) | 0.185 | -0.097 (-0.733, 0.540) | 0.765 |
| Waist, cm |  |  |  |  |  |  |  |
| Model 1 | **-1.475 (-2.681, -0.269)** | **0.017** | reference | **-2.253 (-4.036, -0.470)** | **0.013** | **-2.970 (-5.417, -0.522)** | **0.017** |
| Model 2 | -0.295 (-0.973, 0.384) | 0.395 | reference | -0.657 (-1.648, 0.331) | 0.192 | -0.348 (-1.709, 1.013) | 0.616 |
| Model 3 | -0.437 (-1.153, 0.279) | 0.232 | reference | -0.692 (-1.746, 0.362) | 0.198 | -0.807 (-2.255, 0.640) | 0.274 |
| Model 4 | -0.300 (-0.963, 0.363) | 0.375 | reference | -0.608 (-1.576, 0.361) | 0.219 | -0.405 (-1.738, 0.927) | 0.551 |
| Chest, cm |  |  |  |  |  |  |  |
| Model 1 | -1.159 (-2.325, 0.006) | 0.051 | reference | **-2.027 (-3.751, -0.304)** | **0.021** | **-2.471 (-4.836, -0.106)** | **0.041** |
| Model 2 | 0.090 (-0.399, 0.579) | 0.718 | reference | -0.346 (-1.065, 0.372) | 0.345 | 0.292 (-0.697, 1.280) | 0.563 |
| Model 3 | -0.098 (-0.708, 0.511) | 0.752 | reference | -0.437 (-1.342, 0.467) | 0.343 | -0.268 (-1.510, 0.974) | 0.672 |
| Model 4 | 0.008 (-0.397, 0.573) | 0.723 | reference | -0.325 (-1.039, 0.388) | 0.372 | 0.267 (-0.715, 1.249) | 0.594 |
| Standing long jump, cm |  |  |  |  |  |  |  |
| Model 1 | **3.331 (0.204, 6.458)** | **0.037** | reference | -0.714 (-5.268, 3.840) | 0.759 | **7.921 (1.671, 14.171)** | **0.013** |
| Model 2 | **3.282 (0.129, 6.436)** | **0.041** | reference | -0.814 (-5.400, 3.772) | 0.728 | **7.756 (1.443, 14.069)** | **0.016** |
| Model 3 | 2.999 (-0.134, 6.133) | 0.061 | reference | -1.255 (-5.815, 3.305) | 0.590 | **7.171 (0.913, 13.429)** | **0.025** |
| Model 4 | 3.308 (0.231, 6.385) | 0.035 | reference | -1.071 (-5.539, 3.397) | 0.639 | 8.051 (1.901,14.201 ) | 0.010 |
| Handgrip strength, kg |  |  |  |  |  |  |  |
| Left hand |  |  |  |  |  |  |  |
| Model 1 | **-0.644 (-1.210, -0.079)** | **0.026** | reference | -0.762 (-1.591, 0.067) | 0.072 | **-1.379 (-2.516, -0.242)** | **0.017** |
| Model 2 | -0.338 (-0.845, 0.169) | 0.191 | reference | -0.351 (-1.091, 0.389) | 0.352 | -0.704 (-1.722, 0.314) | 0.175 |
| Model 3 | -0.446 (-0.984, 0.092) | 0.104 | reference | -0.465 (-1.252, 0.323) | 0.248 | -0.967 (-2.048, 0.113) | 0.079 |
| Model 4 | -0.333 (-0.824, 0.157) | 0.182 | reference | -0.398 (-1.113, 0.317) | 0.275 | -0.650 (-1.634, 0.334) | 0.196 |
| Right hand |  |  |  |  |  |  |  |
| Model 1 | **-0.599 (-1.152, -0.045)** | **0.034** | reference | **-0.888 (-1.691, -0.085)** | **0.030** | **-1.169 (-2.271, -0.067)** | **0.038** |
| Model 2 | -0.272 (-0.756, 0.211) | 0.270 | reference | -0.452 (-1.49, 0.245) | 0.204 | -0.452 (-1.412, 0.507) | 0.356 |
| Model 3 | -0.385 (-0.905, 0.134) | 0.146 | reference | -0.570 (-1.323, 0.182) | 0.137 | -0.728 (-1.761, 0.305) | 0.167 |
| Model 4 | -0.267 (-0.731, 0.197) | 0.259 | reference | -0.501 (-1.17, 0.168) | 0.142 | -0.396 (-1.317, 0.524) | 0.399 |

Model 1 adjusted for age, caregivers’ smoking, breakfast, weekly nocturnal sleep time, physical activity time and dietary intake (vegetable, fruit, coarse grain, meat, egg, fish and soybean).

Model 2 adjusted for age, caregivers’ smoking, breakfast, weekly nocturnal sleep time, physical activity time, weight and dietary intake (vegetable, fruit, coarse grain, meat, egg, fish and soybean) (not applicable to weight).

Model 3 adjusted for age, caregivers’ smoking, breakfast, weekly nocturnal sleep time, physical activity time, BMI and dietary intake (vegetable, fruit, coarse grain, meat, egg, fish and soybean) (not applicable to BMI).

Model 4 adjusted for age, caregivers’ smoking, breakfast, weekly nocturnal sleep time, physical activity time, weight, BMI and dietary intake (vegetable, fruit, coarse grain, meat, egg, fish and soybean) (not applicable to weight and BMI).

**Table S13. Association between coarse grain intake and growth and development among boys (n = 285)**

| **Growth and development** | **Coarse grain intake, times/week** | | | | | | | | |
| --- | --- | --- | --- | --- | --- | --- | --- | --- | --- |
|  | **Total (*n* = 285)** | | **< 1 (*n* = 56)** | **1** - **2 (*n* = 104)** | | **3** - **5 (*n* = 84)** | | **> 5 (*n* = 41)** | |
|  | ***β* (95% *CI*)** | ***P*-Value** |  | ***β* (95% *CI*)** | ***P*-Value** | ***β* (95% *CI*)** | ***P*-Value** | ***β* (95% *CI*)** | ***P*-Value** |
| Body height, cm |  |  |  |  |  |  |  |  |  |
| Model 1 | 0.376 (-0.467, 1.218) | 0.382 | reference | 1.280 (-0.846, 3.405) | 0.238 | 0.963 (-1.318, 3.243) | 0.408 | 1.937 (-0.830, 4.704) | 0.170 |
| Model 2 | 0.050 (-0.595, 0.695) | 0.879 | reference | -235 (-1.867, 1.397) | 0.778 | -0.225 (-1.969, 1.519) | 0.800 | 0.691 (-1.423, 2.805) | 0.522 |
| Model 3 | 0.174 (-1.764, 0.356) | 0.667 | reference | 0.358 (-1.648, 2.364) | 0.726 | 0.177 (-1.968, 2.322) | 0.871 | 1.272 (-1.322, 3.866) | 0.337 |
| Model 4 | 0.254 (0.009, 0.498) | 0.042 | reference | 0.575 (-0.039, 1.188) | 0.066 | 0.763 (0.107, 1.420) | 0.023 | 0.836 (0.043, 1.630) | 0.039 |
| Weight, kg |  |  |  |  |  |  |  |  |  |
| Model 1 | 0.700 (-0.469, 1.868) | 0.240 | reference | **3.231 (0.293, 6.168)** | **0.031** | 2.533 (-0.619, 5.685) | 0.115 | 2.658 (-1.167, 6.482) | 0.173 |
| Model 3 | -0.044 (-0.461, 0.373) | 0.835 | reference | -0.119 (-1.172, 0.933) | 0.824 | -0.323 (-1.448, 0.802) | 0.574 | 0.240 (-1.121, 1.602) | 0.729 |
| BMI, kg/m^2^ |  |  |  |  |  |  |  |  |  |
| Model 1 | 0.322 (-0.150, 0.794) | 0.182 | reference | **1.447 (0.261, 2.634)** | **0.017** | 1.234 (-0.039, 2.507) | 0.058 | 1.044 (-0.501, 2.589) | 0.185 |
| Model 2 | 0.057 (-0.111, 0.226) | 0.504 | reference | 0.227 (-0.197, 0.652) | 0.294 | 0.277 (-0.176, 0.731) | 0.231 | 0.041 (-0.509, 0.591) | 0.885 |
| Sitting height, cm |  |  |  |  |  |  |  |  |  |
| Model 1 | 0.020 (-0.489, 0.530) | 0.939 | reference | 0.130 (-1.153, 1.413) | 0.842 | 0.136 (-1.241, 1.513) | 0.846 | 0.207 (-1.463. 1.878) | 0.808 |
| Model 2 | -0.169 (-0.570, 0.233) | 0.410 | reference | -0.764 (-1.760, 0.267) | 0.149 | -0.551 (-1.635, 0.532) | 0.319 | -0.514 (-1.827, 0.799) | 0.443 |
| Model 3 | -0.118 (-0.587, 0.351) | 0.621 | reference | -0.502 (-1.687, 0.684) | 0.407 | -0.402 (-1.670, 0.865) | 0.534 | -0.249 (-1.782, 1.285) | 0.751 |
| Model 4 | -0.081 (-0.388, 0.227) | 0.607 | reference | -0.401 (-1.183, 0.382) | 0.316 | -0.129 (-0.966, 0.708) | 0.763 | -0.452 (-1.464, 0.560) | 0.382 |
| Stature-sitting height index |  |  |  |  |  |  |  |  |  |
| Model 1 | -0.151 (-0.362, 0.061) | 0.163 | reference | -0.404 (-0.935, 0.128) | 0.137 | -0.308 (-0.879, 0.263) | 0.290 | -0.658 (-1.350, 0.035) | 0.063 |
| Model 2 | -0.160 (-0.371, 0.052) | 0.139 | reference | -0.446 (-0.981, 0.089) | 0.102 | -0.341 (-0.913, 0.230) | 0.242 | -0.693 (-1.386,0.000) | 0.050 |
| Model 3 | -0.172 (-0.382, 0.038) | 0.108 | reference | -0.502, -1.033, 0.029) | 0.064 | -0.392 (-0.960, 0.176) | 0.176 | **-0.729 (-1.415, -0.042)** | **0.038** |
| Model 4 | -0.176 (-0.382, 0.030) | 0.094 | reference | -0.514 (-1.034, 0.007) | 0.053 | -0.424 (-0.981, 0.133) | 0.136 | -0.705 (-1.378, -0.031) | 0.040 |
| Body fat rate, % |  |  |  |  |  |  |  |  |  |
| Model 1 | 0.375 (-0.610, 1.359) | 0.456 | reference | 1.639 (-0.806, 4.085) | 0.189 | 1.836 (-0.788, 4.460) | 0.170 | 0.891 (-2.294, 4.075) | 0.584 |
| Model 2 | -0.022 (-0.752, 0.708) | 0.953 | reference | -0.173 (-1.995, 1.649) | 0.853 | 0.415 (-1.533, 2.363) | 0.676 | -0.600 (-2.961, 1.761) | 0.618 |
| Model 3 | -0.133 (-0.779, 0.514) | 0.688 | reference | -0.623 (-2.333, 0.987) | 0.448 | -0.093 (-1.814, 1.628) | 0.916 | -0.742 (-2.824, 1.340) | 0.485 |
| Model 4 | -0.143 (-0.782, 0.497) | 0.662 | reference | -0.651 (-2.242, 0.940) | 0.422 | -0.169 (-1.871, 1.533) | 0.846 | -0.686 (-2.744, 1.372) | 0.514 |
| Fat weight, kg |  |  |  |  |  |  |  |  |  |
| Model 1 | 0.218 (-0.330, 0.765) | 0.436 | reference | 1.251 (-0.113, 2.614) | 0.072 | 1.051 (-0.412, 2.515) | 0.159 | 0.667 (-1.108, 2.443) | 0.461 |
| Model 2 | -0.049 (-0.368, 0.269) | 0.761 | reference | 0.026 (-0.768, 0.819) | 0.949 | 0.091 (-0.757, 0.939) | 0.833 | -0.340 (-1.368, 0.687) | 0.516 |
| Model 3 | -0.094 (-0.396, 0.209) | 0.545 | reference | -0.138 (-0.896, 0.620) | 0.721 | -0.132 (-0.943, 0.678) | 0.749 | -0.335 (-1.315, 0.646) | 0.503 |
| Model 4 | -0.088 (-0.386, 0.210) | 0.563 | reference | -0.122 (-0.867, 0.623) | 0.748 | -0.089 (-0.886, 0.708) | 0.826 | -0.367 (-1.331, 0.597) | 0.455 |
| Waist, cm |  |  |  |  |  |  |  |  |  |
| Model 1 | 0.738 (-0.410, 1.886) | 0.208 | reference | **3.787 (0.910, 6.664)** | **0.010** | 2.565 (-0.522, 5.652) | 0.103 | 2.924 (-0.822, 6.670) | 0.126 |
| Model 2 | 0.122 (-0.390, 0.634) | 0.641 | reference | 0.944 (-0.329, 2.217) | 0.146 | 0.336 (-1.025, 1.697) | 0.629 | 0.585 (-1.064, 2.235) | 0.487 |
| Model 3 | 0.034 (-0.470, 0.539) | 0.893 | reference | 0.632 (-0.640, 1.904) | 0.330 | -0.125 (-1.485, 1.235) | 0.857 | 0.647 (-0.998, 2.292) | 0.441 |
| Model 4 | 0.054 (-0.418, 0.525) | 0.824 | reference | 0.686 (-0.495, 1.866) | 0.255 | 0.020 (-1.242, 1.283) | 0.975 | 0.539 (-0.988, 2.065) | 0.489 |
| Chest, cm |  |  |  |  |  |  |  |  |  |
| Model 1 | 0.426 (-0.489, 1.342) | 0.362 | reference | **2.582 (0.282, 4.883)** | **0.028** | 1.973 (-0.495, 4.441) | 0.117 | 1.482 (-1.513, 4.477) | 0.332 |
| Model 2 | -0.067 (-0.469, 0.335) | 0.744 | reference | 0.308 (-0.708, 1.324) | 0.552 | 0.190 (-0.896, 1.275) | 0.732 | -0.389 (-1.705,0.927) | 0.563 |
| Model 3 | -0.123 (-0.558, 0.312) | 0.579 | reference | 0.118 (-0.993, 1.229) | 0.835 | -0.128 (-1.316, 1.059) | 0.833 | -0.296 (-1.733, 1.140) | 0.686 |
| Model 4 | -0.102 (-0.491, 0.286) | 0.605 | reference | 0.176 (-0.811, 1.163) | 0.727 | 0.028 (-1.027, 1.084) | 0.958 | -0.413 (-1.689, 0.864) | 0.526 |
| Standing long jump, cm |  |  |  |  |  |  |  |  |  |
| Model 1 | 0.685 (-1.997, 3.367) | 0.617 | reference | -3.188 (-9.774, 3.397) | 0.343 | 3.884 (-3.183, 10.950) | 0.281 | 0.805 (-7.770, 9.380) | 0.854 |
| Model 2 | 1.052 (-1.565, 3.669) | 0.431 | reference | -1.512 (-7.970, 4.946) | 0.646 | 5.198(-1.706, 12.102) | 0.140 | 2.184 (-6.185, 10.553) | 0.609 |
| Model 3 | 1.274 (-1.273, 3.820) | 0.327 | reference | -0.597 (-6.893, 5.698) | 0.852 | 6.092 (-0.638, 12.823) | 0.076 | 2.675 (-5.467, 10.816) | 0.520 |
| Model 4 | 1.332 (-1.155, 3.819) | 0.294 | reference | -0.449 (-6.606, 5.709) | 0.886 | 6.495 (-0.091, 13.082) | 0.053 | 2.375 (-5.589, 10.339) | 0.559 |
| Handgrip strength, kg |  |  |  |  |  |  |  |  |  |
| Left hand |  |  |  |  |  |  |  |  |  |
| Model 1 | 0.277 (-0.157, 0.710) | 0.211 | reference | 0.384 (-0.679, 1.446) | 0.479 | 1.067 (-0.073, 2.207) | 0.067 | 0.821 (-0.562, 2.204) | 0.245 |
| Model 2 | 0.166 (-0.227, 0.559) | 0.407 | reference | -0.108 (-1.079, 0.864) | 0.828 | 0.682 (-0.357, 1.720) | 0.198 | 0.417 (-0.842, 1.676) | 0.517 |
| Model 3 | 0.192 (-0.224, 0.608) | 0.366 | reference | 0.014 (-1.015, 1.042) | 0.979 | 0.751 (-0.348, 1.851) | 0.180 | 0.554 (-0.776, 1.884) | 0.414 |
| Model 4 | 0.212 (-0.157, 0.581) | 0.260 | reference | 0.066 (-0.851, 0.984) | 0.888 | 0.894 (-0.088, 1.875) | 0.074 | 0.448 (-0.739, 1.635) | 0.459 |
| Right hand |  |  |  |  |  |  |  |  |  |
| Model 1 | 0.314 (-0.144, 0.772) | 0.179 | reference | 0.420 (-0.707, 1.546) | 0.465 | **1.228 (0.019, 2.437)** | **0.047** | 0.872 (-0.595, 2.340) | 0.244 |
| Model 2 | 0.191 (-0.219, 0.602) | 0.362 | reference | -0.130 (-1.148, 0.887) | 0.802 | 0.796 (-0.292, 1.884) | 0.151 | 0.420 (0.899, 1.739) | 0.533 |
| Model 3 | 0.217 (-0.219, 0.654) | 0.330 | reference | -0.002 (-1.085, 1.080) | 0.996 | 0.868 (-0.290, 2.025) | 0.142 | 0.568 (-0.833, 1.968) | 0.427 |
| Model 4 | 0.239 (-0.147, 0.625) | 0.225 | reference | 0.054 (-0.905, 1.014) | 0.911 | 1.022 (-0.004, 2.048) | 0.051 | 0.453 (-0.788, 1.694) | 0.474 |

Model 1 adjusted for gender, age, caregivers’ smoking, breakfast, weekly nocturnal sleep time, physical activity time and dietary intake (vegetable, fruit, meat, milk, egg, fish and soybean).

Model 2 adjusted for gender, age, caregivers’ smoking, breakfast, weekly nocturnal sleep time, physical activity time, weight and dietary intake (vegetable, fruit, meat, milk, egg, fish and soybean) (not applicable to weight).

Model 3 adjusted for gender, age, caregivers’ smoking, breakfast, weekly nocturnal sleep time, physical activity time, BMI and dietary intake (vegetable, fruit, meat, milk, egg, fish and soybean) (not applicable to BMI).

Model 4 adjusted for gender, age, caregivers’ smoking, breakfast, weekly nocturnal sleep time, physical activity time, weight, BMI and dietary intake (vegetable, fruit, meat, milk, egg, fish and soybean) (not applicable to weight and BMI).

**Table S14. Association between coarse grain intake and growth and development among girls (n = 307)**

| **Growth and development** | **Coarse grain intake, times/week** | | | | | | | | |
| --- | --- | --- | --- | --- | --- | --- | --- | --- | --- |
|  | **Total (*n* = 307)** | | **< 1 (*n* = 57)** | **1** - **2 (*n* = 139)** | | **3** - **5 (*n* = 78)** | | **> 5 (*n* = 33)** | |
|  | ***β* (95% *CI*)** | ***P*-Value** |  | ***β* (95% *CI*)** | ***P*-Value** | ***β* (95% *CI*)** | ***P*-Value** | ***β* (95% *CI*)** | ***P*-Value** |
| Body height, cm |  |  |  |  |  |  |  |  |  |
| Model 1 | **1.047 (0.203, 1.892)** | **0.015** | reference | 2.004 (-0.056, 4.064) | 0.057 | **3.189 (0.883, 5.495)** | **0.007** | **2.871 (0.090, 5.651)** | **0.043** |
| Model 2 | 0.330 (-0.316, 0.977) | 0.317 | reference | 0.876 (-0.698, 2.449) | 0.275 | 1.558 (-0.211, 3.322) | 0.084 | 0.876 (-0.698, 2.449) | 0.275 |
| Model 3 | 0.794 (-0.010, 1.597) | 0.053 | reference | 1.659 (-0.297, 3.616) | 0.096 | **2.730 (0.539, 4.922)** | **0.015** | 2.021 (-0.631, 4.673) | 0.135 |
| Model 4 | -0.050 (-0.322, 0.222) | 0.719 | reference | 0.008 (-0.658, 0.673) | 0.982 | 0.079 (-0.671, 0.828) | 0.837 | -0.223 (-1.125, 0.679) | 0.628 |
| Weight, kg |  |  |  |  |  |  |  |  |  |
| Model 1 | **1.289 (0.297, 2.282)** | **0.011** | reference | 2.045 (-0.379, 4.469) | 0.098 | **2.960 (0.246, 5.673)** | **0.033** | **4.071 (0.799, 7.344)** | **0.015** |
| Model 3 | 0.445 (0.046, 0.845) | 0.029 | reference | 0.875 (-0.100, 1.851) | 0.079 | 1.405 (0.312, 2.498) | 0.012 | 1.189 (-0.133, 2.511) | 0.078 |
| BMI, kg/m^2^ |  |  |  |  |  |  |  |  |  |
| Model 1 | 0.352 (-0.027, 0.730) | 0.069 | reference | 0.488 (-0.438, 1.415) | 0.302 | 0.649 (-0.388, 1.686) | 0.220 | 1.203 (-0.048, 2.453) | 0.059 |
| Model 2 | -0.099 (-0.253, 0.054) | 0.204 | reference | -0.227 (-0.601, 0.146) | 0.233 | -0.387 (-0.807, 0.033) | 0.071 | -0.222 (-0.729, 0.285) | 0.391 |
| Sitting height, cm |  |  |  |  |  |  |  |  |  |
| Model 1 | **0.569 (0.069, 1.070)** | **0.026** | reference | 1.038 (-0.193, 2.269) | 0.098 | **1.497 (0.119, 2.875** | **0.033** | **1.681 (0.019, 3.342)** | **0.047** |
| Model 2 | 0.145 (-0.238, 0.529) | 0.458 | reference | 0.362 (-0.577, 1.301) | 0.449 | 0.519 (-0.535, 1.574) | 0.334 | 0.336 (-0.938, 1.610) | 0.605 |
| Model 3 | 0.378 (-0.080, 0.837) | 0.106 | reference | 0.772 (-0.353, 1.897) | 0.178 | 1.144 (-0.116, 2.403) | 0.075 | 1.026 (-0.499, 2.550) | 0.187 |
| Model 4 | -0.008 (-0.311, 0.296) | 0.960 | reference | 0.014 (-0.732, 0.761) | 0.970 | -0.073 (-0.913, 0.768) | 0.865 | -0.004 (-1.015, 1.008) | 0.994 |
| Stature-sitting height index |  |  |  |  |  |  |  |  |  |
| Model 1 | 0.002 (-0.203, 0.208) | 0.981 | reference | -0.050 (-0.559, 0.459) | 0.847 | -0.158 (-0.728, 0.411) | 0.586 | 0.080 (-0.607, 0.766) | 0.821 |
| Model 2 | -0.024 (-0.230, 0.183) | 0.823 | reference | -0.097 (-0.605, 0.411) | 0.708 | -0.227 (-0.797, 0.344) | 0.436 | -0.014 (-0.704, 0.675) | 0.967 |
| Model 3 | -0.035 (-0.237, 0.168) | 0.735 | reference | -0.106 (-0.604, 0.393) | 0.678 | -0.232 (-0.791, 0.326) | 0.415 | -0.058 (-0.734, 0.618) | 0.867 |
| Model 4 | 0.013 (-0.187, 0.212) | 0.902 | reference | -0.014 (-0.505, 0.476) | 0.954 | -0.086 (-0.638, 0.467) | 0.761 | 0.067 (-0.598, 0.731) | 0.845 |
| Body fat rate, % |  |  |  |  |  |  |  |  |  |
| Model 1 | 0.747 (-0.301, 1.795) | 0.162 | reference | 0.824 (-1.752, 3.400) | 0.531 | 0.776 (-2.107, 3.660) | 0.598 | 2.561 (-0.917, 6.039) | 0.149 |
| Model 2 | -0.339 (-0.978, 0.300) | 0.299 | reference | -0.932 (-2.457, 0.593) | 0.231 | **-1.765 (-3.477, -0.053)** | **0.043** | -0.934 (-3.003, 1.135) | 0.376 |
| Model 3 | -0.121 (-0.597, 0.355) | 0.619 | reference | -0.398 (-1.523, 0.727) | 0.488 | -0.847 (-2.108, 0.413) | 0.188 | -0.449 (-1.974, 1.076) | 0.564 |
| Model 4 | -0.062 (-0.539, 0.415) | 0.798 | reference | -0.304 (-1.430, 0.822) | 0.596 | -0.697 (-1.965, 0.571) | 0.281 | -0.322 (-1.848, 1.204) | 0.679 |
| Fat weight, kg |  |  |  |  |  |  |  |  |  |
| Model 1 | **0.599 (0.028, 1.170)** | **0.040** | reference | 0.875 (-0.532, 2.282) | 0.223 | 0.986 (-0.589, 2.561) | 0.220 | **2.057 (0.157, 3.956)** | **0.034** |
| Model 2 | -0.061 (-0.326, 0.204) | 0.653 | reference | -0.186 (-0.820, 0.448) | 0.566 | -0.549 (-1.261, 0.163) | 0.131 | -0.055 (-0.916, 0.805) | 0.900 |
| Model 3 | 0.122 (-0.129, 0.373) | 0.341 | reference | 0.206 (-0.403, 0.816) | 0.507 | 0.098 (-0.585, 0.780) | 0.779 | 0.409 (-0.417, 1.235) | 0.332 |
| Model 4 | 0.019 (-0.216, 0.254) | 0.875 | reference | -0.007 (-0.571, 0.556) | 0.980 | -0.246 (-0.880, 0.389) | 0.448 | 0.119 (-0.645, 0.883) | 0.761 |
| Waist, cm |  |  |  |  |  |  |  |  |  |
| Model 1 | **1.029 (0.141, 1.917)** | **0.023** | reference | 1.064 (-1.111, 3.240) | 0.338 | 1.490 (-0.945, 3.925) | 0.230 | **3.301 (0.364, 6.238)** | **0.028** |
| Model 2 | 0.071 (-0.429, 0.572) | 0.780 | reference | -0.468 (-1.671, 0.735) | 0.446 | -0.728 (-2.078, 0.623) | 0.291 | 0.251 (-1.381, 1.883) | 0.763 |
| Model 3 | 0.363 (-0.163, 0.889) | 0.176 | reference | 0.136 (-1.143, 1.415) | 0.835 | 0.256 (-1.177, 1.689) | 0.726 | 1.014 (-0.719, 2.748) | 0.251 |
| Model 4 | 0.140 (-0.350, 0.630) | 0.576 | reference | -0.319 (-1.499, 0.861) | 0.596 | -0.474 (-1.803, 0.855) | 0.484 | 0.396 (-1.203, 1.996) | 0.627 |
| Chest, cm |  |  |  |  |  |  |  |  |  |
| Model 1 | **1.053 (0.195, 1.911)** | **0.016** | reference | 1.575 (-0.527, 3.677) | 0.142 | 2.119 (-0.234, 4.473) | 0.078 | **3.491 (0.653, 6.329)** | **0.016** |
| Model 2 | 0.039 (-0.322, 0.399) | 0.834 | reference | -0.040 (-0.913, 0.834) | 0.929 | -0.217 (-1.198, 0.764) | 0.664 | 0.276 (-0.909, 1.462) | 0.648 |
| Model 3 | 0.372 (-0.076, 0.820) | 0.104 | reference | 0.629 (-0.468, 1.727) | 0.261 | 0.863 (-0.367, 2.092) | 0.169 | 1.161 (-0.327, 2.649) | 0.126 |
| Model 4 | 0.068 (-0.290, 0.427) | 0.708 | reference | 0.024 (-0.846, 0.893) | 0.957 | -0.110 (-1.089, 0.870) | 0.826 | 0.338 (-0.841, 1.517) | 0.574 |
| Standing long jump, cm |  |  |  |  |  |  |  |  |  |
| Model 1 | 0.826 (-1.476, 3.127) | 0.482 | reference | **6.318 (0.763, 11.874)** | **0.026** | **8.377 (2.158, 14.596)** | **0.008** | 0.172 (-7.328, 7.672) | 0.964 |
| Model 2 | 0.865 (-1.460, 3.191) | 0.466 | reference | **6.415 (0.836, 11.994)** | **0.024** | **8.516 (2.253, 14.780)** | **0.008** | 0.364 (-7.207, 7.935) | 0.925 |
| Model 3 | 1.039 (-1.264, 3.341) | 0.377 | reference | **6.640 (1.109, 12.172)** | **0.019** | **8.805 (2.608, 15.001)** | **0.005** | 0.965 (-6.533, 8.463) | 0.801 |
| Model 4 | 0.536 (-1.740, 2.811) | 0.644 | reference | 5.644 (0.198, 11.091) | 0.042 | 7.206 (1.073, 13.339) | 0.021 | -0.388 (-7.77, 6.994) | 0.918 |
| Handgrip strength, kg |  |  |  |  |  |  |  |  |  |
| Left hand |  |  |  |  |  |  |  |  |  |
| Model 1 | 0.181 (-0.235, 0.598) | 0.394 | reference | -0.721 (-1.732, 0.290) | 0.162 | 0.438 (-0.694, 1.569) | 0.449 | -0.349 (-1.714, 1.015) | 0.616 |
| Model 2 | -0.067 (-0.441, 0.307) | 0.725 | reference | **-1.115 (-2.015, -0.215)** | **0.015** | -0.134 (-1.144, 0.877) | 0.795 | -1.135 (-2.356, 0.087) | 0.069 |
| Model 3 | 0.054 (-0.342, 0.449) | 0.790 | reference | -0.898 (-1.853, 0.058) | 0.065 | 0.203 (-0.867, 1.272) | 0.711 | -0.785 -2.079, 0.510) | 0.235 |
| Model 4 | -0.129 (-0.492, 0.233) | 0.484 | reference | **-1.257 (-2.128, -0.385)** | **0.005** | -0.374 (-1.355, 0.607) | 0.455 | **-1.273 (-2.454, -0.091)** | **0.035** |
| Right hand |  |  |  |  |  |  |  |  |  |
| Model 1 | **0.480 (0.072, 0.887)** | **0.021** | reference | -0.113 (-1.092, 0.867) | 0.822 | **1.415 (0.319, 2.511)** | **0.011** | 0.332 (-0.990, 1.655) | 0.622 |
| Model 2 | 0.215 (-0.142, 0.571) | 0.238 | reference | -0.531 (-1.379, 0.317) | 0.219 | 0.809 (-0.143, 1.761) | 0.096 | -0.501 (-1.652, 0.649) | 0.393 |
| Model 3 | 0.343 (-0.039, 0.725) | 0.079 | reference | -0.302 (-1.215, 0.611) | 0.517 | **1.164 (0.141, 2.186)** | **0.026** | -0.133 (-1.371, 1.104) | 0.833 |
| Model 4 | 0.150 (-0.193, 0.493) | 0.392 | reference | -0.677 (-1.493,0.138 ) | 0.103 | 0.560 (-0.357, 1.478) | 0.231 | -0.644 (-1.749,0.461 ) | 0.253 |

Model 1 adjusted for gender, age, caregivers’ smoking, breakfast, weekly nocturnal sleep time, physical activity time and dietary intake (vegetable, fruit, meat, milk, egg, fish and soybean).

Model 2 adjusted for gender, age, caregivers’ smoking, breakfast, weekly nocturnal sleep time, physical activity time, weight and dietary intake (vegetable, fruit, meat, milk, egg, fish and soybean) (not applicable to weight).

Model 3 adjusted for gender, age, caregivers’ smoking, breakfast, weekly nocturnal sleep time, physical activity time, BMI and dietary intake (vegetable, fruit, meat, milk, egg, fish and soybean) (not applicable to BMI).

Model 4 adjusted for gender, age, caregivers’ smoking, breakfast, weekly nocturnal sleep time, physical activity time, weight, BMI and dietary intake (vegetable, fruit, meat, milk, egg, fish and soybean) (not applicable to weight and BMI).

**Table S15. Association between soybean intake and growth and development among boys (n = 285)**

| **Growth and development** | **Soybean intake, times/week** | | | | | | |
| --- | --- | --- | --- | --- | --- | --- | --- |
|  | **Total (*n* = 285)** | | **< 1 (*n* = 73)** | **1** - **2 (*n* = 120)** | | **> 2 (*n* = 92)** | |
|  | ***β* (95% *CI*)** | ***P*-Value** |  | ***β* (95% *CI*)** | ***P*-Value** | ***β* (95% *CI*)** | ***P*-Value** |
| Body height, cm |  |  |  |  |  |  |  |
| Model 1 | -1.125 (-2.249, -0.001) | 0.050 | reference | -1.585 (-3.554, 0.383) | 0.114 | **-2.453 (-4.710, -0.195)** | **0.033** |
| Model 2 | -0.299 (-1.165, 0.568) | 0.499 | reference | -0.836 (-2.338, 0.667) | 0.276 | -0.771 (-2.506,0.964) | 0.384 |
| Model 3 | -0.704 (-1.764, 0.356) | 0.193 | reference | -1.205 (-3.049, 0.638) | 0.200 | -1.616 (-3.741, 0.509) | 0.136 |
| Model 4 | -0.297 (-0.625, 0.031) | 0.076 | reference | -0.810 (-1.374, -0.246) | 0.005 | -0.628 (-1.279, 0.023) | 0.059 |
| Weight, kg |  |  |  |  |  |  |  |
| Model 1 | **-1.775 (-3.334, -0.216)** | **0.026** | reference | -1.599 (-4.320, 1.122) | 0.249 | **-3.587 (-6.708, -0.467)** | **0.024** |
| Model 3 | -0.226 (-0.784, 0.333) | 0.429 | reference | -0.218 (-1.185, 0.750) | 0.659 | -0.544 (-1.660, 0.571) | 0.339 |
| BMI, kg/m^2^ |  |  |  |  |  |  |  |
| Model 1 | **-0.670 (-1.300, -0.040)** | **0.037** | reference | -0.597 (-1.696, 0.502) | 0.287 | **-1.315 (-2.575, -0.054)** | **0.041** |
| Model 2 | 0.000 (-0.226, 0.227) | 0.997 | reference | 0.007 (-0.384, 0.398) | 0.971 | 0.040 (-0.411, 0.491) | 0.862 |
| Sitting height, cm |  |  |  |  |  |  |  |
| Model 1 | -0.485 (-1.165, 0.195) | 0.162 | reference | -0.740 (-1.928, 0.449) | 0.222 | -1.071 (-2.434, 0.292) | 0.124 |
| Model 2 | -0.006 (-0.545, 0.533) | 0.982 | reference | -0.306 (-1.239, 0.628) | 0.521 | -0.097 (-1.175, 0.980) | 0.860 |
| Model 3 | -0.197 (-0.825, 0.431) | 0.539 | reference | -0.479 (-1.569, 0.610) | 0.389 | -0.497 (-1.753, 0.759) | 0.438 |
| Model 4 | -0.006 (-0.418, 0.407) | 0.979 | reference | -0.295 (-1.014, 0.424) | 0.422 | -0.036 (-0.866, 0.794) | 0.932 |
| Stature-sitting height index |  |  |  |  |  |  |  |
| Model 1 | 0.089 (-0.193, 0.372) | 0.536 | reference | 0.061 (-0.432, 0.554) | 0.808 | 0.191 (-0.374. 0.756) | 0.509 |
| Model 2 | 0.113 (-0.171, 0.397) | 0.436 | reference | 0.082 (-0.410, 0.575) | 0.744 | 0.238 (-0.331, 0.806) | 0.412 |
| Model 3 | 0.134 (-0.147, 0.415) | 0.351 | reference | 0.102 (-0.386, 0.590) | 0.683 | 0.280 (-0.283, 0.842) | 0.330 |
| Model 4 | 0.113 (-0.164, 0.389) | 0.425 | reference | 0.080 (-0.399, 0.559) | 0.743 | 0.226 (-0.327, 0.778) | 0.423 |
| Body fat rate, % |  |  |  |  |  |  |  |
| Model 1 | **-1.857 (-3.170, -0.543)** | **0.006** | reference | -2.153 (-4.418, 0.113) | 0.063 | **-3.531 (-6.129, -0.932)** | **0.008** |
| Model 2 | -0.851 (-1.831, 0.130) | 0.089 | reference | -1.256 (-2.934, 0.422) | 0.142 | -1.518 (-3.455, 0.419) | 0.124 |
| Model 3 | -0.801 (-1.667, 0.066) | 0.070 | reference | -1.220 (-2.699, 0.260) | 0.106 | -1.475 (-3.181, 0.230) | 0.090 |
| Model 4 | -0.852 (-1.710, 0.006) | 0.052 | reference | -1.271 (-2.733, 0.192) | 0.089 | -1.603 (-3.291, 0.085) | 0.063 |
| Fat weight, kg |  |  |  |  |  |  |  |
| Model 1 | **-1.008 (-1.738, -0.278)** | **0.007** | reference | -0.994 (-2.257, 0.269) | 0.123 | **-1.866 (-3.315, -0.418)** | **0.012** |
| Model 2 | -0.331 (-0.758, 0.096) | 0.129 | reference | -0.387 (-1.118, 0.343) | 0.299 | -0.506 (-1.350, 0.337) | 0.239 |
| Model 3 | -0.360 (-0.766, 0.045) | 0.082 | reference | -0.421 (-1.118, 0.276) | 0.236 | -0.605 (-1.409, 0.198) | 0.140 |
| Model 4 | -0.331 (-0.731, 0.068) | 0.104 | reference | -0.392 (-1.077, 0.293) | 0.262 | -0.532 (-1.323, 0.258) | 0.187 |
| Waist, cm |  |  |  |  |  |  |  |
| Model 1 | **-2.269 (-3.801, -0.737)** | **0.004** | reference | **-3.050 (-5.715, -0.385)** | **0.025** | **-4.498 (-7.555, -1.442)** | **0.004** |
| Model 2 | **-0.707 (-1.395, -0.020)** | **0.044** | reference | **-1.643 (-2.816, -0.471)** | **0.006** | **-1.341 (-2.695, 0.012)** | **0.052** |
| Model 3 | **-0.805 (-1.481, -0.128)** | **0.020** | reference | **-1.749 (-2.918, -0.581)** | **0.003** | **-1.632 (-2.980, -0.285)** | **0.018** |
| Model 4 | -0.708 (-1.340, -0.075) | 0.028 | reference | -1.651 (-2.736, -0.566) | 0.003 | -1.387 (-2.639, -0.135) | 0.030 |
| Chest, cm |  |  |  |  |  |  |  |
| Model 1 | **-1.662 (-2.884, -0.440)** | **0.008** | reference | -1.900 (-4.031, 0.231) | 0.080 | **-3.291 (-5.735, -0.847)** | **0.008** |
| Model 2 | -0.411 (-0.951, 0.128) | 0.135 | reference | -0.774 (-1.710, 0.161) | 0.105 | -0.765 (-1.845, 0.314) | 0.165 |
| Model 3 | -0.518 (-1.101, 0.065) | 0.082 | reference | -0.884 (-1.905, 0.137) | 0.090 | -1.052 (-2.229, 0.124) | 0.080 |
| Model 4 | -0.412 (-0.932, 0.109) | 0.121 | reference | -0.779 (-1.686, 0.129) | 0.093 | -0.789 (-1.836, 0.258) | 0.140 |
| Standing long jump, cm |  |  |  |  |  |  |  |
| Model 1 | -0.089 (-3.667, 3.489) | 0.961 | reference | 0.232 (-5.867, 6.332) | 0.940 | -1.156 (-8.151, 5.839) | 0.746 |
| Model 2 | -1.020 (-4.534, 2.494) | 0.569 | reference | -0.598 (-6.545, 5.350) | 0.844 | -3.018 (-9.883, 3.848) | 0.389 |
| Model 3 | -1.315 (-4.728, 2.098) | 0.450 | reference | -0.836 (-6.621, 4.949) | 0.777 | -3.509 (-10.179, 3.160) | 0.302 |
| Model 4 | -1.017 (-4.354, 2.319) | 0.550 | reference | -0.564 (-6.224, 5.096) | 0.845 | -2.830 (-9.364, 3.703) | 0.396 |
| Handgrip strength, kg |  |  |  |  |  |  |  |
| Left hand |  |  |  |  |  |  |  |
| Model 1 | -0.289 (-0.867, 0.289) | 0.327 | reference | -0.302 (-1.286, 0.682) | 0.547 | -0.656 (-1.784, 0.473) | 0.255 |
| Model 2 | -0.009 (-0.536, 0.519) | 0.974 | reference | -0.059 (-0.954, 0.836) | 0.897 | -0.110 (-1.143, 0.923) | 0.834 |
| Model 3 | -0.112 (-0.670, 0.446) | 0.693 | reference | -0.150 (-1.095, 0.795) | 0.756 | -0.320 (-1.409, 0.770) | 0.565 |
| Model 4 | -0.008 (-0.504, 0.487) | 0.974 | reference | -0.054 (-0.897, 0.790) | 0.901 | -0.080 (-1.053, 0.894) | 0.873 |
| Right hand |  |  |  |  |  |  |  |
| Model 1 | -0.568 (-1.179, 0.043) | 0.069 | reference | -0.664 (-1.708, 0.379) | 0.212 | \| **-1.209 (-2.406, -0.013)** \| \| --- \| | **0.048** |
| Model 2 | -0.256 (-0.808, 0.295) | 0.362 | reference | -0.392 (-1.329, 0.545) | 0.412 | -0.599 (-1.681, 0.483) | 0.278 |
| Model 3 | -0.367 (-0.952, 0.219) | 0.220 | reference | -0.490 (-1.485, 0.505) | 0.334 | -0.826 (-1.973, 0.321) | 0.158 |
| Model 4 | -0.256 (-0.774, 0.262) | 0.333 | reference | -0.386 (-1.268, 0.496) | 0.391 | -0.566 (-1.584, 0.452) | 0.276 |

Model 1 adjusted for age, caregivers’ smoking, breakfast, weekly nocturnal sleep time, physical activity time and dietary intake (vegetable, fruit, meat, milk, egg, fish and coarse grain).

Model 2 adjusted for age, caregivers’ smoking, breakfast, weekly nocturnal sleep time, physical activity time, weight and dietary intake (vegetable, fruit, meat, milk, egg, fish and coarse grain) (not applicable to weight).

Model 3 adjusted for age, caregivers’ smoking, breakfast, weekly nocturnal sleep time, physical activity time, BMI and dietary intake (vegetable, fruit, meat, milk, egg, fish and coarse grain) (not applicable to BMI).

Model 4 adjusted for age, caregivers’ smoking, breakfast, weekly nocturnal sleep time, physical activity time, weight, BMI and dietary intake (vegetable, fruit, meat, milk, egg, fish and coarse grain) (not applicable to weight and BMI).

**Table S16. Association between soybean intake and growth and development among girls (n = 307)**

| **Growth and development** | **Soybean intake, times/week** | | | | | | |
| --- | --- | --- | --- | --- | --- | --- | --- |
|  | **Total (*n* = 307)** | | **< 1 (*n* = 71)** | **1** - **2 (*n* = 125)** | | **> 2 (*n* = 111)** | |
|  | ***β* (95% *CI*)** | ***P*-Value** |  | ***β* (95% *CI*)** | ***P*-Value** | ***β* (95% *CI*)** | ***P*-Value** |
| Body height, cm |  |  |  |  |  |  |  |
| Model 1 | -0.021 (-1.064, 1.021) | 0.968 | reference | -0.055 (-1.966, 1.856) | 0.955 | -0.031 (-2.125, 2.063) | 0.977 |
| Model 2 | 0.452 (-0.340, 1.243) | 0.264 | reference | 0.531 (-0.924, 1.987) | 0.474 | 0.894 (-0.703, 2.492) | 0.272 |
| Model 3 | 0.293 (-0.699, 1.285) | 0.563 | reference | 0.359 (-1.458, 2.177) | 0.699 | 0.584 (-1.413, 2.580) | 0.567 |
| Model 4 | -0.078 (-0.412, 0.256) | 0.646 | reference | -0.286 (-0.902, 0.330) | 0.363 | -0.184 (-0.860, 0.493) | 0.594 |
| Weight, kg |  |  |  |  |  |  |  |
| Model 1 | -0.850 (-2.075, 0.374) | 0.173 | reference | -1.062 (-3.311, 1.187) | 0.355 | -1.677 (-4.141, 0.788) | 0.182 |
| Model 3 | 0.196 (-0.297, 0.689) | 0.436 | reference | 0.342 (-0.564, 1.248) | 0.460 | 0.407 (-0.589, 1.402) | 0.423 |
| BMI, kg/m^2^ |  |  |  |  |  |  |  |
| Model 1 | -0.436 (-0.903, 0.032) | 0.068 | reference | -0.586 (-1.445, 0.274) | 0.182 | -0.869 (-1.811, 0.072) | 0.070 |
| Model 2 | -0.138 (-0.326, 0.049) | 0.148 | reference | -0.214 (-0.560, 0.132) | 0.225 | -0.283 (-0.662, 0.097) | 0.144 |
| Sitting height, cm |  |  |  |  |  |  |  |
| Model 1 | -0.010 (-0.627, 0.608) | 0.976 | reference | -0.045 (-1.187, 1.097) | 0.939 | -0.029 (-1.281, 1.222) | 0.963 |
| Model 2 | 0.270 (-0.200, 0.740) | 0.260 | reference | 0.306 (-0.563, 1.174) | 0.490 | 0.524 (-0.429, 1.478) | 0.281 |
| Model 3 | 0.227 (-0.339, 0.793) | 0.432 | reference | 0.048 (-0.415, 0.511) | 0.838 | 0.086 (-0.422, 0.595) | 0.739 |
| Model 4 | 0.057 (-0.315, 0.429) | 0.763 | reference | -0.022 (-0.713, 0.668) | 0.950 | 0.092 (-0.667, 0.851) | 0.812 |
| Stature-sitting height index |  |  |  |  |  |  |  |
| Model 1 | -0.001 (-0.255, 0.252) | 0.991 | reference | -0.018 (-0.491, 0.454) | 0.939 | -0.013 (-0.530, 0.505) | 0.961 |
| Model 2 | 0.016 (-0.237, 0.269) | 0.903 | reference | 0.006 (-0.464, 0.476) | 0.980 | 0.026 (-0.490, 0.542) | 0.922 |
| Model 3 | 0.045 (-0.205, 0.295) | 0.724 | reference | 0.048 (-0.415, 0.511) | 0.838 | 0.086 (-0.422, 0.595) | 0.739 |
| Model 4 | 0.066 (-0.179, 0.310) | 0.597 | reference | 0.084 (-0.370, 0.538) | 0.717 | 0.129 (-0.370, 0.627) | 0.613 |
| Body fat rate, % |  |  |  |  |  |  |  |
| Model 1 | **-1.380 (-2.673, -0.087)** | **0.036** | reference | **-3.086 (-5.476, -0.696)** | **0.011** | **-3.054 (-5.673, -0.435)** | **0.022** |
| Model 2 | -0.664 (-1.446, 0.119) | 0.097 | reference | **-2.175 (-3.585, -0.764)** | **0.003** | **-1.614 (-3.162, -0.067)** | **0.041** |
| Model 3 | -0.304 (-0.891, 0.283) | 0.310 | reference | **-1.620 (-2.666, -0.575)** | **0.002** | -0.878 (-2.027, 0.270) | 0.134 |
| Model 4 | -0.278 (-0.863, 0.306) | 0.351 | reference | -1.584 (-2.626, -0.542) | 0.003 | -0.835 (-1.979, 0.309) | 0.153 |
| Fat weight, kg |  |  |  |  |  |  |  |
| Model 1 | -0.658 (-1.363, 0.047) | 0.067 | reference | -1.308 (-2.614, -0.003) | 0.050 | \| -1.430 (-2.861, 0.000) \| \| --- \| | 0.050 |
| Model 2 | -0.223 (-0.548, 0.101) | 0.178 | reference | **-0.757 (-1.344, -0.171)** | **0.011** | -0.560 (-1.204, 0.083) | 0.088 |
| Model 3 | -0.067 (-0.377, 0.243) | 0.672 | reference | -0.506 (-1.072, 0.060) | 0.080 | -0.239 (-0.861, 0.382) | 0.450 |
| Model 4 | -0.112 (-0.401, 0.176) | 0.445 | reference | -0.589 (-1.111, -0.068) | 0.027 | -0.339 (-0.911, 0.234) | 0.246 |
| Waist, cm |  |  |  |  |  |  |  |
| Model 1 | **-1.405 (-2.500, -0.309)** | **0.012** | reference | **-2.153 (-4.171, -0.134)** | **0.037** | **-2.792 (-5.003, -0.580)** | **0.013** |
| Model 2 | **-0.773 (-1.386, -0.160)** | **0.013** | reference | **-1.357 (-2.470, 0.244)** | **0.017** | **-1.535 (-2.756, -0.314)** | **0.014** |
| Model 3 | -0.579 (-1.228, 0.070) | 0.081 | reference | -1.039 (-2.227, 0.149) | 0.087 | -1.139 (-2.444, 0.166) | 0.087 |
| Model 4 | -0.677 (-1.278, -0.076) | 0.027 | reference | -1.217 (-2.309, -0.125) | 0.029 | -1.350 (-2.550, -0.151) | 0.027 |
| Chest, cm |  |  |  |  |  |  |  |
| Model 1 | -0.766 (-1.825, 0.292) | 0.156 | reference | -1.291 (-3.241, 0.660) | 0.195 | -1.512 (-3.649, 0.625) | 0.166 |
| Model 2 | -0.098 (-0.539, 0.344) | 0.665 | reference | -0.452 (-1.261, 0.356) | 0.273 | -0.188 (-1.075, 0.699) | 0.677 |
| Model 3 | 0.077 (-0.475, 0.630) | 0.784 | reference | -0.156 (-1.176, 0.863) | 0.764 | 0.172 (-0.948, 1.291) | 0.764 |
| Model 4 | -0.056 (-0.496, 0.383) | 0.802 | reference | -0.393 (-1.197, 0.412) | 0.339 | -0.110 (-0.994, 0.774) | 0.808 |
| Standing long jump, cm |  |  |  |  |  |  |  |
| Model 1 | 1.040 (-1.800, 3.880) | 0.473 | reference | -0.055 (-5.210, 5.100) | 0.983 | 1.076 (-4.572, 6.724) | 0.709 |
| Model 2 | 1.014 (-1.835, 3.863) | 0.485 | reference | -0.105 (-5.266, 5.056) | 0.968 | 0.977 (-4.667, 6.660) | 0.730 |
| Model 3 | 0.776 (-2.065, 3.618) | 0.592 | reference | -0.441 (-5.579, 4.697) | 0.866 | 0.503 (-5.141, 6.147) | 0.861 |
| Model 4 | 0.555 (-2.234, 3.344) | 0.152 | reference | -0.830 (-5.868, 4.208) | 0.747 | 0.04 (-5.495, 5.575) | 0.989 |
| Handgrip strength, kg |  |  |  |  |  |  |  |
| Left hand |  |  |  |  |  |  |  |
| Model 1 | 0.287 (-0.227, 0.801) | 0.274 | reference | -0.150 (-1.088, 0.788) | 0.754 | 0.500 (-0.527, 1.528) | 0.340 |
| Model 2 | 0.451 (-0.007, 0.909) | 0.054 | reference | 0.055 (-0.778, 0.888) | 0.897 | 0.824 (-0.090, 1.737) | 0.077 |
| Model 3 | 0.445 (-0.043, 0.933) | 0.074 | reference | 0.062 (-0.825, 0.949) | 0.891 | 0.815 (-0.159, 1.790) | 0.101 |
| Model 4 | 0.364 (-0.008, 0.809) | 0.108 | reference | -0.078 (-0.884, 0.728) | 0.850 | 0.648 (-0.237, 1.534) | 0.151 |
| Right hand |  |  |  |  |  |  |  |
| Model 1 | 0.149 (-0.354, 0.651) | 0.563 | reference | -0.374 (-1.282, 0.535) | 0.420 | 0.065 (-0.931, 1.061) | 0.898 |
| Model 2 | 0.323 (-0.113, 0.760) | 0.147 | reference | -0.156 (-0.941, 0.628) | 0.696 | 0.408 (-0.452, 1.269) | 0.352 |
| Model 3 | 0.318 (-0.153, 0.789) | 0.186 | reference | -0.147 (-0.995, 0.701) | 0.734 | 0.402 (-0.530, 1.333) | 0.398 |
| Model 4 | 0.233 (-0.187, 0.654 ) | 0.277 | reference | -0.294 (-1.048,0.461 ) | 0.445 | 0.227 (-0.601, 1.056) | 0.591 |

Model 1 adjusted for age, caregivers’ smoking, breakfast, weekly nocturnal sleep time, physical activity time and dietary intake (vegetable, fruit, meat, milk, egg, fish and coarse grain).

Model 2 adjusted for age, caregivers’ smoking, breakfast, weekly nocturnal sleep time, physical activity time, weight and dietary intake (vegetable, fruit, meat, milk, egg, fish and coarse grain) (not applicable to weight).

Model 3 adjusted for age, caregivers’ smoking, breakfast, weekly nocturnal sleep time, physical activity time, BMI and dietary intake (vegetable, fruit, meat, milk, egg, fish and coarse grain) (not applicable to BMI).

Model 4 adjusted for age, caregivers’ smoking, breakfast, weekly nocturnal sleep time, physical activity time, weight, BMI and dietary intake (vegetable, fruit, meat, milk, egg, fish and coarse grain) (not applicable to weight and BMI).

**Table S17. Association between vegetable intake and growth and development among boys (n = 285)**

| **Growth and development** | **Vegetable intake, times/week** | | | | | | |
| --- | --- | --- | --- | --- | --- | --- | --- |
|  | **Total (*n* = 285)** | | **< 3 (*n* = 66)** | **3** - **5 (*n* = 72)** | | **> 5 (*n* = 147)** | |
|  | ***β* (95% *CI*)** | ***P*-Value** |  | ***β* (95% *CI*)** | ***P*-Value** | ***β* (95% *CI*)** | ***P*-Value** |
| Body height, cm |  |  |  |  |  |  |  |
| Model 1 | 0.320 (-0.638, 1.279) | 0.513 | reference | -0.573 (-2.774, 1.628) | 0.610 | 0.568 (-1.394, 2.529) | 0.570 |
| Model 2 | -0.128 (-0.863, 0.607) | 0.732 | reference | -1.334 (-3.014, 0.345) | 0.119 | -0.473(-1.974, 1.027) | 0.537 |
| Model 3 | 0.122 (-0.778, 1.021) | 0.791 | reference | -1.119 (-3.183, 0.945) | 0.288 | 0.056 (-1.784, 1.896) | 0.952 |
| Model 4 | -0.296 (-0.575, -0.017) | 0.037 | reference | -0.463 (-1.095, 0.168) | 0.150 | -0.597 (-1.160, -0.033) | 0.038 |
| Weight, kg |  |  |  |  |  |  |  |
| Model 1 | 0.964 (-0.365, 2.294) | 0.155 | reference | 1.624 (-1.418, 4.667) | 0.295 | 2.220 (-0.491, 4.931) | 0.108 |
| Model 3 | 0.231 (-0.243, 0.705) | 0.339 | reference | -0.361 (-1.444, 0.722) | 0.513 | 0.360 (-0.606, 1.325) | 0.465 |
| BMI, kg/m^2^ |  |  |  |  |  |  |  |
| Model 1 | 0.317 (-0.221, 0.854) | 0.248 | reference | 0.858 (-0.371, 2.087) | 0.171 | 0.804 (-0.291, 1.899) | 0.150 |
| Model 2 | -0.047 (-0.239, 0.144) | 0.629 | reference | 0.244 (-0.193, 0.681) | 0.273 | -0.035 (-0.425, 0.356) | 0.862 |
| Sitting height, cm |  |  |  |  |  |  |  |
| Model 1 | 0.468 (-0.112, 1.047) | 0.114 | reference | 0.051 (-1.278, 1.380) | 0.940 | 0.870 (-0.314, 2.074) | 0.150 |
| Model 2 | 0.208 (-0.250, 0.665) | 0.373 | reference | -0.390 (-1.433, 0.653) | 0.464 | 0.267 (-0.665, 1.199) | 0.574 |
| Model 3 | 0.331 (-0.202, 0.865) | 0.223 | reference | -0.324 (-1.544, 0.896) | 0.603 | 0.519 (-0.568, 1.606) | 0.349 |
| Model 4 | 0.135 (-0.215, 0.485) | 0.450 | reference | -0.018 (-0.824, 0.788) | 0.965 | 0.215 (-0.504, 0.933) | 0.558 |
| Stature-sitting height index |  |  |  |  |  |  |  |
| Model 1 | 0.219 (-0.022, 0.460) | 0.074 | reference | 0.275 (-0.276, 0.826) | 0.328 | 0.419 (-0.072, 0.910) | 0.094 |
| Model 2 | 0.206 (-0.035, 0.447) | 0.093 | reference | 0.254 (-0.297, 0.804) | 0.367 | 0.390 (-0.102, 0.882) | 0.120 |
| Model 3 | 0.198 (-0.041, 0.437) | 0.104 | reference | 0.217 (-0.330, 0.763) | 0.437 | 0.365 (-0.122, 0.852) | 0.142 |
| Model 4 | 0.200 (-0.015, 0.455) | 0.067 | reference | 0.181 (-0.355, 0.717) | 0.509 | 0.400 (-0.077, 0.878) | 0.101 |
| Body fat rate, % |  |  |  |  |  |  |  |
| Model 1 | 0.762 (-0.358, 1.882) | 0.182 | reference | 0.767 (-1.766, 3.300) | 0.553 | 1.570 (-0.687, 3.827) | 0.173 |
| Model 2 | 0.216 (-0.616 1.048) | 0.611 | reference | -0.144 (-2.020, 1.732) | 0.880 | 0.325 (-1.351, 2.000) | 0.704 |
| Model 3 | 0.263 (-0.472, 0.998) | 0.484 | reference | -0.574 (-2.231, 1.082) | 0.497 | 0.313 (-1.163, 1.790) | 0.678 |
| Model 4 | 0.315 (-0.413, 1.043) | 0.396 | reference | -0.659 (-2.297, 0.980) | 0.431 | 0.398 (-1.063, 1.858) | 0.594 |
| Fat weight, kg |  |  |  |  |  |  |  |
| Model 1 | 0.501 (-0.121, 1.124) | 0.115 | reference | 0.788 (-0.625, 2.200) | 0.274 | 1.038 (-0.221, 2.296) | 0.106 |
| Model 2 | 0.133 (-0.229, 0.496) | 0.471 | reference | 0.172 (-0.645, 0.988) | 0.680 | 0.196 (-0.534, 0.925) | 0.599 |
| Model 3 | 0.195 (-0.149, 0.539) | 0.267 | reference | -0.035 (-0.816, 0.745) | 0.929 | 0.266 (-0.429, 0.962) | 0.453 |
| Model 4 | 0.165 (-0.174, 0.504) | 0.340 | reference | 0.013 (-0.754, 0.780) | 0.974 | 0.218 (-0.466, 0.902) | 0.531 |
| Waist, cm |  |  |  |  |  |  |  |
| Model 1 | 1.073 (-0.234, 2.379) | 0.108 | reference | 2.656 (-0.324, 5.636) | 0.081 | 2.627 (-0.028, 5.283) | 0.052 |
| Model 2 | 0.224 (-0.359, 0.808) | 0.451 | reference | 1.227 (-0.084, 2.537) | 0.067 | 0.673 (-0.497, 1.844) | 0.260 |
| Model 3 | 0.380 (-0.193, 0.954) | 0.194 | reference | 0.786 (-0.523, 2.095) | 0.239 | 0.875 (-0.292, 2.041) | 0.142 |
| Model 4 | 0.281 (-0.256, 0.818) | 0.306 | reference | 0.949 (-0.266, 2.164) | 0.126 | 0.713 (-0.371, 1.796) | 0.197 |
| Chest, cm |  |  |  |  |  |  |  |
| Model 1 | 0.675 (-0.367, 1.717) | 0.204 | reference | 1.899 (-0.483, 4.282) | 0.118 | 1.675 (-0.448, 3.798) | 0.122 |
| Model 2 | -0.005 (-0.462, 0.453) | 0.984 | reference | 0.756 (-0.290, 1.801) | 0.157 | 0.112 (-0.822, 1.046) | 0.815 |
| Model 3 | 0.134 (-0.361, 0.628) | 0.596 | reference | 0.439 (-0.704, 1.582) | 0.452 | 0.306 (-0.713, 1.325) | 0.556 |
| Model 4 | 0.025 (-0.417, 0.467) | 0.913 | reference | 0.613 (-0.403, 1.630) | 0.237 | 0.132 (-0.774, 1.038) | 0.775 |
| Standing long jump, cm |  |  |  |  |  |  |  |
| Model 1 | 1.954 (-1.098, 5.006) | 0.209 | reference | **-9.413 (-16.234, -2.592)** | **0.007** | 1.708 (-4.370, 7.786) | 0.582 |
| Model 2 | 2.460 (-0.522, 5.442) | 0.106 | reference | **-8.570 (-15.219, -1.922)** | **0.012** | 2.860 (-3.079, 8.800) | 0.345 |
| Model 3 | 2.534 (-0.362, 5.430) | 0.086 | reference | **-7.878 (-14.355, -1.400)** | **0.017** | 3.147 (-2.627, 8.921) | 0.285 |
| Model 4 | 2.229 (-0.603, 5.061) | 0.123 | reference | -7.427 (-13.767, -1.087) | 0.022 | 2.698 (-2.954, 8.350) | 0.349 |
| Handgrip strength, kg |  |  |  |  |  |  |  |
| Left hand |  |  |  |  |  |  |  |
| Model 1 | -0.013 (-0.506, 0.480) | 0.958 | reference | -0.488 (-1.588, 0.612) | 0.385 | -0.111 (-1.091, 0.870) | 0.825 |
| Model 2 | -0.165 (-0.613, 0.282) | 0.469 | reference | -0.735 (-1.735, 0.265) | 0.150 | -0.448 (-1.342, 0.445) | 0.325 |
| Model 3 | -0.097 (-0.570, 0.377) | 0.689 | reference | -0.707 (-1.765, 0.351) | 0.190 | -0.316 (-1.259, 0.627) | 0.511 |
| Model 4 | -0.203 (-0.624, 0.217) | 0.344 | reference | -0.548 (-1.493, 0.397) | 0.256 | -0.475 (-1.317, 0.367) | 0.269 |
| Right hand |  |  |  |  |  |  |  |
| Model 1 | 0.389 (-0.132, 0.910) | 0.144 | reference | 0.029 (-1.138, 1.196) | 0.962 | 0.742 (-0.;298, 1.781) | 0.162 |
| Model 2 | 0.220 (-0.248, 0.687) | 0.357 | reference | -0.248 (-1.296, 0.800) | 0.643 | 0.363 (-0.573, 1.299) | 0.447 |
| Model 3 | 0.294 (-0.203, 0.790) | 0.247 | reference | -0.222 (-1.336, 0.893) | 0.697 | 0.507 (-0.486, 1.500) | 0.317 |
| Model 4 | 0.180 (-0.260, 0.620) | 0.422 | reference | -0.049 (-1.037, 0.939) | 0.922 | 0.335 (-0.545, 1.216) | 0.455 |

Model 1 adjusted for age, caregivers’ smoking, breakfast, weekly nocturnal sleep time, physical activity time and dietary intake (vegetable, fruit, meat, milk, egg, fish and coarse grain).

Model 2 adjusted for age, caregivers’ smoking, breakfast, weekly nocturnal sleep time, physical activity time, weight and dietary intake (fruit, meat, coarse grain, milk, egg, fish and soybean) (not applicable to weight).

Model 3 adjusted for age, caregivers’ smoking, breakfast, weekly nocturnal sleep time, physical activity time, BMI and dietary intake (fruit, meat, coarse grain, milk, egg, fish and soybean) (not applicable to BMI).

Model 4 adjusted for age, caregivers’ smoking, breakfast, weekly nocturnal sleep time, physical activity time, weight, BMI and dietary intake (fruit, meat, coarse grain, milk, egg, fish and soybean) (not applicable to weight and BMI).

**Table S18. Association between vegetable intake and growth and development among girls (n = 307)**

| **Growth and development** | **Vegetable intake, times/week** | | | | | | |
| --- | --- | --- | --- | --- | --- | --- | --- |
|  | **Total (*n* = 307)** | | **< 3 (*n* = 63)** | **3** - **5 (*n* = 97)** | | **> 5 (*n* = 147)** | |
|  | ***β* (95% *CI*)** | ***P*-Value** |  | ***β* (95% *CI*)** | ***P*-Value** | ***β* (95% *CI*)** | ***P*-Value** |
| Body height, cm |  |  |  |  |  |  |  |
| Model 1 | -0.228 (-1.173, 0.717) | 0.636 | reference | -0.042 (-2.031, 1.946) | 0.967 | -0.603 (-2.526, 1.321) | 0.539 |
| Model 2 | -0.118 (-0.834, 0.598) | 0.747 | reference | 0.604 (-0.911, 2.118) | 0.435 | -0.220 (-1.684, 1.243) | 0.768 |
| Model 3 | -0.139 (-1.034, 0.756) | 0.761 | reference | 0.379 (-1.511, 2.269) | 0.694 | -0.353 (-2.179, 1.473) | 0.705 |
| Model 4 | -0.325 (-0.626, -0.024) | 0.034 | reference | -0.108 (-0.749, 0.532) | 0.740 | -0.644 (-1.262, -0.025) | 0.041 |
| Weight, kg |  |  |  |  |  |  |  |
| Model 1 | -0.198 (-1.309, 0.912) | 0.726 | reference | -1.171 (-3.510, 1.169) | 0.327 | -0.693 (-2.956, 1.571) | 0.549 |
| Model 3 | 0.099 (-0.346, 0.543) | 0.665 | reference | 0.258 (-0.684, 1.201) | 0.591 | 0.154 (-0.756, 1.064) | 0.740 |
| BMI, kg/m^2^ |  |  |  |  |  |  |  |
| Model 1 | -0.124 (-0.547, 0.300) | 0.568 | reference | -0.596 (-1.490, 0.298) | 0.191 | -0.353 (-1.218, 0.512) | 0.423 |
| Model 2 | -0.054 (-0.224, 0.115) | 0.531 | reference | -0.187 (-0.546, 0.173) | 0.309 | -0.111 (-0.459, 0.237) | 0.532 |
| Sitting height, cm |  |  |  |  |  |  |  |
| Model 1 | 0.146 (-0.414, 0.707) | 0.608 | reference | 0.177 (-1.011, 1.365) | 0.770 | 0.208 (-0.941, 1.357) | 0.723 |
| Model 2 | 0.212 (-0.213, 0.636) | 0.329 | reference | 0.564 (-0.340, 1.468) | 0.221 | 0.437 (-0.437, 1.310) | 0.327 |
| Model 3 | 0.213 (-0.297, 0.724) | 0.413 | reference | 0.502 (-0.585, 1.588) | 0.365 | 0.400 (-0.649, 1.450) | 0.455 |
| Model 4 | 0.128 (-0.207, 0.463) | 0.454 | reference | 0.278 (-0.440, 0.996) | 0.447 | 0.267 (-0.426, 0.960) | 0.450 |
| Stature-sitting height index |  |  |  |  |  |  |  |
| Model 1 | 0.210 (-0.019, 3.219) | 0.073 | reference | 0.181 (-0.311, 0.672) | 0.471 | 0.420 (-0.056, 0.895) | 0.084 |
| Model 2 | 0.214 (-0.014, 0.443) | 0.066 | reference | 0.208 (-0.281, 0.696) | 0.405 | 0.436 (-0.037, 0.908) | 0.071 |
| Model 3 | 0.224 (-0.002, 0.449) | 0.052 | reference | 0.249 (-0.233, 0.730) | 0.312 | 0.460 (-0.005, 0.925) | 0.053 |
| Model 4 | 0.234 (0.014, 0.455) | 0.038 | reference | 0.276 (-0.196, 0.747) | 0.252 | 0.476 (0.020, 0.932) | 0.041 |
| Body fat rate, % |  |  |  |  |  |  |  |
| Model 1 | -0.066 (-1.238, 1.107) | 0.913 | reference | -0.940 (-3.426, 1.547) | 0.459 | -0.182 (-2.588, 2.223) | 0.882 |
| Model 2 | 0.101 (-0.606, 0.809) | 0.779 | reference | 0.065 (-1.402, 1.533) | 0.931 | 0.413 (-1.006, 1.831) | 0.569 |
| Model 3 | 0.239 (-0.291, 0.769) | 0.376 | reference | 0.552 (-0.535, 1.640) | 0.319 | 0.702 (-0.348, 1.752) | 0.190 |
| Model 4 | 0.252 (-0.275, 0.779) | 0.348 | reference | 0.580 (-0.503, 1.663) | 0.294 | 0.719 (-0.327, 1.764) | 0.178 |
| Fat weight, kg |  |  |  |  |  |  |  |
| Model 1 | 0.141 (-0.498, 0.781) | 0.665 | reference | -0.275 (-1.633, 1.083) | 0.691 | 0.242 (-1.072, 1.556) | 0.718 |
| Model 2 | 0.243 (-0.051, 0.536) | 0.105 | reference | 0.322 (-0.278, 0.942) | 0.286 | **0.602 (0.12, 1.191)** | **0.046** |
| Model 3 | **0.309 (0.029, 0.589)** | **0.030** | reference | 0.541 (-0.047, 1.130) | 0.071 | **0.726 (0.158, 1.295)** | **0.012** |
| Model 4 | 0.286 (0.026, 0.546) | 0.031 | reference | 0.478 (-0.064, 1.020) | 0.084 | 0.689 (0.165, 1.212) | 0.010 |
| Waist, cm |  |  |  |  |  |  |  |
| Model 1 | 0.390 (-0.604, 1.384) | 0.442 | reference | -0.990 (-3.090, 1.110) | 0.356 | 0.461 (-1.751, 2.493) | 0.657 |
| Model 2 | 0.537 (-0.017, 1.092) | 0.057 | reference | -0.113 (-1.270, 1.045) | 0.849 | 0.980 (-0.139, 2.099) | 0.086 |
| Model 3 | **0.624 (0.038, 1.210)** | **0.037** | reference | 0.144 (-1.092, 1.380) | 0.820 | 1.133 (-0.061, 2.326) | 0.063 |
| Model 4 | 0.575 (0.033, 1.117) | 0.038 | reference | 0.010 (-1.126, 1.145) | 0.987 | 1.053 (-0.043, 2.149) | 0.060 |
| Chest, cm |  |  |  |  |  |  |  |
| Model 1 | -0.089 (-1.049, 0.871) | 0.856 | reference | -0.490 (-2.519, 1.539) | 0.636 | -0.308 (-2.727, 1.655) | 0.758 |
| Model 2 | 0.067 (-0.333, 0.466) | 0.743 | reference | 0.434 (-0.407, 1.275) | 0.312 | 0.239 (-0.574, 1.051) | 0.565 |
| Model 3 | 0.150 (-0.349, 0.649) | 0.556 | reference | 0.685 (-0.396, 1.725) | 0.219 | 0.376 (-0.648, 1.400) | 0.472 |
| Model 4 | 0.083 (-0.313, 0.479) | 0.682 | reference | 0.486 (-0.351, 1.322) | 0.255 | 0.269 (-0.538, 1.077) | 0.513 |
| Standing long jump, cm |  |  |  |  |  |  |  |
| Model 1 | -0.383 (-2.959, 2.192) | 0.770 | reference | -3.276 (-8.639, 2.086) | 0.231 | -1.011 (-6.199, 4.177) | 0.702 |
| Model 2 | -0.389 (-2.966, 2.187) | 0.767 | reference | -3.332 (-8.701, 2.038) | 0.224 | -1.044 (-6.234, 4.146) | 0.693 |
| Model 3 | -0.458 (-3.023, 2.106) | 0.726 | reference | -3.669 (-9.014, 1.675) | 0.178 | -1.244 (-6.406, 3.918) | 0.637 |
| Model 4 | -0.569 (-3.085, 1.946) | 0.657 | reference | -3.963 (-9.202, 1.275) | 0.138 | -1.420 (-6.478, 3.638) | 0.582 |
| Handgrip strength, kg |  |  |  |  |  |  |  |
| Left hand |  |  |  |  |  |  |  |
| Model 1 | -0.226 (-0.692, 0.240) | 0.342 | reference | -0.252 (-1.228, 0.724) | 0.613 | -0.467 (-1.411, 0.477) | 0.332 |
| Model 2 | -0.188 (-0.602, 0.226) | 0.374 | reference | -0.026 (-0.892, 0.840) | 0.953 | -0.333 (-1.171, 0.504) | 0.435 |
| Model 3 | -0.181 (-0.622, 0.259) | 0.420 | reference | -0.036 (-0.958, 0.887) | 0.939 | -0.339 (-1.230, 0.552) | 0.456 |
| Model 4 | -0.222 (-0.622, 0.179) | 0.278 | reference | -0.142 (-0.980, 0.696) | 0.740 | -0.402 (-1.212, 0.407) | 0.330 |
| Right hand |  |  |  |  |  |  |  |
| Model 1 | -0.237 (-0.693, 0.219) | 0.308 | reference | -0.578 (-1.523, 0.368) | 0.231 | -0.456 (-1.371, 0.459) | 0.328 |
| Model 2 | -0.197 (-0.591, 0.198) | 0.329 | reference | -0.338 (-1.154, 0.478) | 0.417 | 0.314 (-1.103, 0.475) | 0.435 |
| Model 3 | -0.189 (-0.615, 0.236) | 0.383 | reference | -0.347 (-1.229, 0.535) | 0.441 | -0.319 (-1.171, 0.533) | 0.463 |
| Model 4 | -0.232 (-0.611, 0.148) | 0.231 | reference | -0.458 (-1.242, 0.326) | 0.253 | -0.385 (-1.142, 0.372) | 0.319 |

Model 1 adjusted for age, caregivers’ smoking, breakfast, weekly nocturnal sleep time, physical activity time and dietary intake (vegetable, fruit, meat, milk, egg, fish and coarse grain).

Model 2 adjusted for age, caregivers’ smoking, breakfast, weekly nocturnal sleep time, physical activity time, weight and dietary intake (fruit, meat, coarse grain, milk, egg, fish and soybean) (not applicable to weight).

Model 3 adjusted for age, caregivers’ smoking, breakfast, weekly nocturnal sleep time, physical activity time, BMI and dietary intake (fruit, meat, coarse grain, milk, egg, fish and soybean) (not applicable to BMI).

Model 4 adjusted for age, caregivers’ smoking, breakfast, weekly nocturnal sleep time, physical activity time, weight, BMI and dietary intake (fruit, meat, coarse grain, milk, egg, fish and soybean) (not applicable to weight and BMI).
